# Do legislated carbon reduction targets influence pro-environmental behaviours in public hospital pharmacy departments? Using mixed methods to compare Australia and the UK

**DOI:** 10.1371/journal.pone.0255445

**Published:** 2021-08-18

**Authors:** Judith A. Singleton, Esther T-L. Lau, Lisa M. Nissen

**Affiliations:** 1 School of Clinical Sciences, Faculty of Health, Queensland University of Technology (QUT), Brisbane, Queensland, Australia; 2 Institute of Health and Biomedical Research (IHBI), Queensland University of Technology (QUT), Brisbane, Queensland, Australia; University of Brescia, ITALY

## Abstract

Pharmaceuticals and their packaging have a significant negative impact on the environment providing a very strong argument for action on the part of pharmacists and pharmacy technicians to engage with pro-environmental behaviours (PEBs) in their workplaces. The aims of this research were therefore to investigate in hospital pharmacists and pharmacy technicians, 1) factors affecting engagement with workplace PEBs, and 2) determine if legislated carbon reduction targets in the UK influenced workplace PEBs in the UK compared with Australia which does not have legislated carbon reduction targets. The environmentally responsible disposal of pharmaceutical waste was the PEB of interest in this study. A mixed methods research design was utilised and a conceptual model (key variables: environmental attitude, concern, and knowledge, and organisational factors) was developed to identify factors influencing workplace PEBs. Participants were from five hospitals in Queensland, Australia and five NHS hospitals in England, UK. There was no statistically significant difference in environmental attitude or concern between the two groups—most had a mid-environmental attitude score and low levels of environmental concern. Participants lacked knowledge of the issue and the link between the environment and public health. Both Australian and UK participants reported recycling packaging waste was not a priority in the hospital pharmacy workplace (even in hospitals with recycling capability) as hospitals focused on compliance with clinical (contaminated) and confidential waste streams. Environmental attitude, knowledge, and concern therefore appeared to be weak influences on intention to perform workplace PEBs with workplace social norms (compliance due to audits) appearing to be a significant mediator of action. The key difference between the cohorts was that UK pharmacists felt waste was not in the scope of their role, and therefore not their responsibility. This study identified that legislated carbon reduction targets did not influence hospital pharmacy workplace PEBs–neither cohort reported engaging significantly in workplace PEBs. UK Government and NHS sustainability policy did not appear to have disseminated to pharmacy department level of UK public hospitals to any great extent.

## Introduction

Healthcare has a significant carbon footprint. For example, healthcare’s carbon footprint comprised approximately 3% of the UK’s total CO_2_ equivalent (CO_2_e) emissions in 2010 [1], 10% of the USA’s total CO_2_e emissions in 2016 [2], and 7% of Australia’s total CO_2_e emissions in 2014–15 [3]. The National Health Service (NHS) Sustainable Development Unit (SDU) was established in 2008 [4] to develop a strategy for the NHS England to meet its carbon reduction targets under the Climate Change Act of 2008 [5]. In January 2009 the NHS SDU published a Carbon Reduction Strategy for the NHS—a global first, which included a carbon footprint of the NHS [6]. In contrast, despite being one of the world’s largest emitters, Australia has no mitigation policy in place (or even being developed) nor climate change legislation [7, 8]. Unsurprisingly, sustainable development objectives do not appear in Australian State Health Departments’ strategic plans.

A large contributor to healthcare’s carbon footprint is pharmaceuticals. For example, pharmaceuticals comprised 22% of NHS England’s total carbon footprint in 2010 [1], and 19% of Australia’s healthcare carbon footprint in 2014–15 [3]. Pharmaceuticals have a large negative impact on the environment. This negative impact is due to the embedded carbon in their manufacture and distribution, and through the waste generated in their manufacture, consumption, and disposal [9]. There are both negative impacts on the health of humans [10–12] and on the health of wildlife [13–15] originating from three sources [16]: 1) the carbon footprint of pharmaceuticals through the embedded carbon in their manufacture and distribution and the incineration of unwanted pharmaceuticals and original containers, 2) the chemical effects of the pharmaceuticals themselves, and, 3) their packaging waste. Packaging waste contributes significantly to the carbon footprint of pharmaceuticals. The NHS Sustainable Development Unit identified that reducing pharmaceutical wastage was one of eight interventions that would generate cost savings to the NHS and reduce its carbon footprint [17]. Thus, the argument for pharmacists and pharmacy technicians to adopt sustainable workplace practices is a strong one since, paradoxically, the delivery of pharmaceutical care has such a large impact on the health of ecosystems and therefore on the health of humans.

This research compared the workplace pro-environmental behaviours (PEBs) of two cohorts of hospital pharmacists and pharmacy technicians–one from a country with legislated carbon reduction targets (UK) and one from a country with none (Australia). For the purposes of this research and based upon the definitions of Stern [18], Kollmuss and Agyeman [19], and Ones and Dilchert [20, 21], a PEB in this research is defined as ‘actions and behaviours that employees consciously engage in to minimise the negative impact of the organisation’s activities on the natural and built world.’ The environmentally responsible disposal of pharmaceutical waste was selected as the PEB of interest in this study since pharmaceuticals and their packaging have a significant negative impact on the environment. Pharmaceutical waste comprises the pharmaceuticals themselves, original containers, any product inserts such as plastic spoons or measures, and paper or cardboard packaging waste. It is deemed either contaminated (unwanted pharmaceuticals) or non-contaminated (original containers and product inserts such as plastic spoons or measures, and paper or cardboard packaging waste). The aims of this research were therefore to, 1) investigate factors affecting the engagement of hospital pharmacists and pharmacy technicians with the environmentally responsible handling of pharmaceutical waste and, 2) determine if legislated carbon reduction targets influenced workplace PEBs in hospital pharmacists and pharmacy technicians in Australia compared with the UK.

## Methods

### Theoretical framework and research design

**Fig 1:**
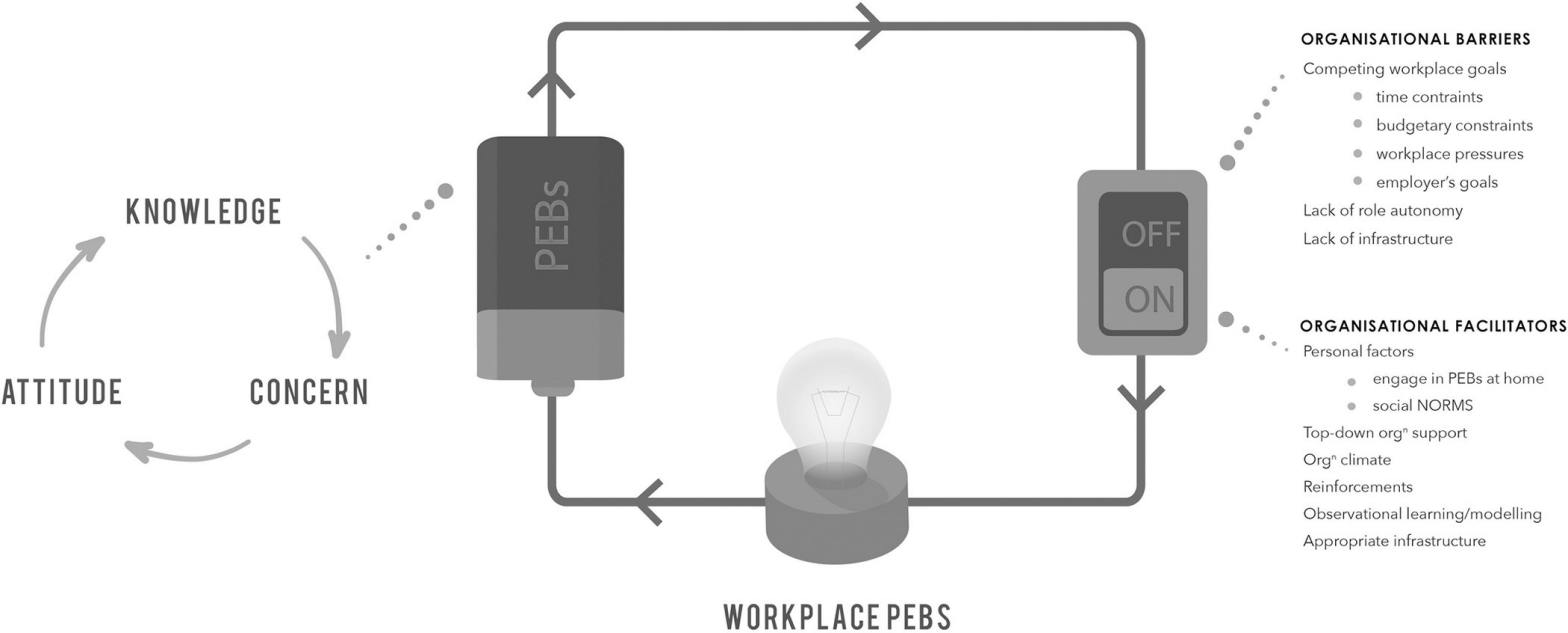
Conceptual framework showing predictor variables of interest for workplace PEBs—environmental attitude, environmental knowledge, environmental concern, and organisational facilitators and barriers.

The theoretical framework underpinning this research was developed from the Theory of Planned Behaviour [22, 23], The Value-Belief-Norm theory [24] which linked value theory to the New Ecological Paradigm (NEP) scale [25, 26] of attitudinal and affective antecedents of PEBs, Social Cognitive Theory [27], Locus of Control theory [28], Model of Responsible Environmental Behaviour [29], Self-Determination Theory and Motivation [30], the Reasonable Person Model [31, 32] Bamberg and Moser’s Theoretical Framework [33], Goal Framing Theory [34–36], and organisational factors influencing PEBs in the workplace [37–41]. Across all the theories and models reviewed, the most common variables were ‘social norms’, ‘knowledge of issue (problem awareness)’, ‘knowledge of action strategies’, ‘environmental attitude’, ‘locus of control’, and ‘self-interest’. Kolmuss and Agyeman found that situational factors reduced the predictability of these variables [19]. Since organisational factors such as availability and convenience of infrastructure will mediate the ability of ‘self-interest’ to predict workplace PEBs, and organisational factors such as workplace policies and procedures will mediate the ability of ‘locus of control’ and ‘social norms’ to predict workplace PEBs (and in the workplace ‘social norms’ may also mediate ‘self-interest’), these three variables were not selected as variables in their own right. Hence, ‘environmental knowledge’ (encompassing knowledge of the environmental issue and knowledge of an action strategy i.e. what to do and how to do it), ‘environmental concern’, and ‘environmental attitude’ were selected as three variables of interest for this research along with ‘organisational facilitators and barriers’ (Fig 1).

The research variables are represented as a circuit (Fig 1) to depict the research hypothesis that an individual’s knowledge of an issue and knowledge of action strategy, combined with a sufficient level of environmental concern, and a pro-environmental attitude influences their intention to perform a PEB. A circular relationship between these three variables has been identified [42]–as depicted in Fig 1. As one variable increases, the other variables are also likely to increase thereby increasing the likelihood of the individual engaging with PEBs. However, regardless of the strength of this intention to behave, if organisational barriers are in place, for example, lack of appropriate infrastructure such as recycling bins, the PEB cannot occur (is switched off). If an individual’s intention to perform a PEB (as influenced by environmental knowledge, attitude, and concern) and organisational facilitators such as a pro-environmental organisational culture and climate, and appropriate infrastructure are present, then the switch is turned on and PEBs are more likely to occur.

Four research objectives were developed to address the research aim. These were: explore the, 1) environmental attitudes, 2) level of environmental knowledge (knowledge of issue or problem awareness–the impact of pharmaceuticals on the environment), and knowledge of action strategies–how to dispose of pharmaceutical waste appropriately), and 3) level of environmental concern of hospital pharmacists and pharmacy technicians as well as, 4) organisational facilitators and barriers to PEBs in their workplaces. Achieving these four objectives required a mixed methods research design with combined sequential and concurrent phases [43]. There were two phases (Phase I: Australia and Phase II: UK) which utilised the same methods.

### Participants

Participants in both phases were hospital pharmacists and pharmacy technicians from all hierarchical levels across five hospitals in regional and metropolitan areas in Queensland, Australia (Phase I; n = 66) and five NHS hospitals in four regional UK (England) cities (Phase II; n = 40). Once hospital sites were selected using purposive recruitment, the directors of the pharmacy departments in these hospitals were emailed information about the research project and disseminated information on the research to their staff. Interested staff were invited to contact the chief researcher directly if they were happy to participate in the study. Participants were provided with an information sheet on the research beforehand and signed an Informed Consent document prior to the interview. Confidentiality was ensured through the creation of a unique identifier code for each participant. The allocation of the identifier code enabled participants’ NEP score to be matched to their demographic information and interview responses.

### Data collection instruments and process

Ethical clearance was obtained from Queensland Health (HREC/14/QPAH/491), Ramsay Health (Protocol 15/34) and Queensland University of Technology (QUT Approval Number 160000080) prior to data collection. Ethical clearance was not required by NHS England for the UK arm of the study (Phase II as ethical clearance had been provided by QUT).

Attitudinal antecedents of workplace PEBs in participants were explored using the New Ecological Paradigm (NEP). The NEP Scale is a multiple-item scale designed to determine an individual’s environmental beliefs and attitudes [26]. It has been used for over thirty years in many different participant groups across multiple countries with demonstrated reliability and validity [26]. This research used the 15-item NEP scale employing a 5-point Likert scale response format for each item (SA-A-N-D-SD) (S1 File). The eight odd-numbered items on the scale were worded in such a way that agreement demonstrates a pro-ecological worldview. The seven even-numbered items on the scale were worded in such a way that disagreement demonstrates a pro-ecological worldview [44]. An endorsement of the NEP (i.e. a high score) is taken to mean a pro-environmental worldview or orientation [44]. Scores for the eight odd-numbered items were allocated as SA–5 points, A-4 points, N-3 points, D-2 points, and SD-1 point. The seven even-numbered items were scored as SA-1 point, A-2 points, N-3 points, D-4 points, and SD-5 points. Scores were allocated into the categories of pro-environmental (59–75), mid-environmental (39–58), and anti-environmental (0–38) [44].

After completing the NEP Scale questionnaire participants were then asked a series of questions (S2 File) to explore their environmental knowledge, environmental concern, and organisational facilitators and barriers to PEBs in their hospital workplaces. Participants were interviewed in a semi-structured interview format (approximately 15 minutes) with interviews audio recorded. Interview transcripts were transcribed using *intelligent verbatim*.

### Data analysis

Quantitative data (NEP scores and interview questions that yielded quantitative data (e.g. Yes/No responses)), were analysed using statistical testing with the computer software programme IBM SPSS Version 23. For the statistical tests NEP scores were treated as continuous variables. Internal reliability of the 15-item NEP scale instrument was tested in each cohort using Cronbach’s alpha (Australian cohort: 0.815; UK cohort: 0.734; combined cohort: 0.787) demonstrating a reasonably high internal consistency.

Qualitative interview transcript data were analysed by two different methods. First, the data were analysed using the text analytics tool, Leximancer®, to provide a global context to the data, and second, the data were coded manually following a three pass coding methodology described by Saldaña [45]. Using two methods provided triangulation of analysis methods. Each method contributed insight to the data in different ways.

Leximancer® converts lexical co-occurrence information in text from natural language into semantic patterns in an automated manner and displays the extracted information visually as a concept map. Concepts in Leximancer® are collections of words that generally appear together throughout the text. One output function of Leximancer® is the ‘Insight Dashboard’ report (including a quadrant graphic) to compare different categories, which was ideal for comparing Australian participants’ responses with UK participants’ responses for identical questions (S3 File). The quadrant coordinates are each concept’s ‘Prominence’ score. Prominence scores are absolute measures of the correlation between a concept and the Category (in this case Australia and the UK) [46].

For the manual coding process, interview transcripts were analysed in three passes or cycles. The first cycle employed structural (or holistic) coding. During the second cycle some codes were merged into a single category as they were conceptually similar. In the third coding cycle, these reorganised categories were progressed up to more general, higher-level themes; their collective meaning derived from the ways they systematically interrelated. Inter-rater reliability was addressed by having a second researcher also code a sample of the same data. Final codes were derived through discussion.

## Results

The Australian cohort comprised 41 pharmacists and 25 pharmacy technicians (n = 66) working at one private hospital and four public hospitals operated by Queensland Health. Purposive sampling techniques were used to ensure a mix of participants across all operational levels. Demographic information is presented in Table 1.

**Table 1:** Demographic information for Australian cohort.

Gender			Region		Total
			Regional	Metropolitan	
Male	Operational Level	Director/Assistant Directors	1	2	3
		Senior pharmacists	3	2	5
		Early career pharmacists	2	6	8
		Pharmacy Interns	0	1	1
		Pharmacy students	0	0	0
		Senior Pharmacy Technicians	0	0	0
		Pharmacy Technicians	0	1	1
Total			6	12	18
Female	Operational Level	Director/Assistant Directors	1	2 (1)	3 (2)
		Senior pharmacists	2	4 (3)	6 (5)
		Early career pharmacists	8	6	14
		Pharmacy Interns	0	3	3
		Pharmacy students	0	1	1
		Senior Pharmacy Technicians	0	2	2
		Pharmacy Technicians	7	12	19
Total			18	30	48 (46)
Overall Totals			24	42	66 (64)^a^

^a^ The figures in brackets correct for the two female pharmacists who completed the NEP questionnaire but were unable to be interviewed due to work time constraints.

The UK cohort comprised 23 pharmacists and 17 pharmacy technicians (n = 40) from six NHS England hospitals in the UK cities of Cambridge, Leeds, Sheffield and York. Purposive sampling techniques were again used to ensure a mix of participants across all operational levels. In total, ten senior pharmacists, eight mid-band pharmacists, five junior pharmacists, 10 senior pharmacy technicians and seven base-level pharmacy technicians were interviewed. Participants comprised 18 males (45%) and 22 (55%) females (Table 2).

**Table 2:** Demographic information for UK cohort.

Gender			City				Total
			Cambridge	Leeds	Sheffield	York	
**Male**	**Operational Level**	Senior Pharmacists	0	2	3	2	7
		Mid-band Pharmacists	2	0	0	1	3
		Junior Pharmacists	0	2	1	0	3
		Senior Pharmacy Technicians	0	2	0	0	2
		Pharmacy Technicians	1	0	1	1	3
**Total Male**			3	6	5	4	18
**Female**	**Operational Level**	Senior Pharmacists	1	1	0	1	3
		Mid-band Pharmacists	0	3	2	0	5
		Junior Pharmacists	1	0	1	0	2
		Senior Pharmacy Technicians	1	3	1	3	8
		Pharmacy Technicians	0	4	0	0	4
**Total Female**			3	11	4	4	22
**Total**	**Operational Level**	Senior Pharmacists	1	3	3	3	10
		Mid-band Pharmacists	2	3	2	1	8
		Junior Pharmacists	1	2	2	0	5
		Senior Pharmacy Technicians	1	5	1	3	10
		Pharmacy Technicians	1	4	1	1	7
	**Total**		**6**	**17**	**9**	**8**	**40**

### Environmental attitude—NEP scores

Statistical testing was performed using IBM SPSS Version 23. The statistical analytical plans are provided in supporting information (S4 File). Three research questions were tested:

1. Is there a difference in mean NEP scores between Australian pharmacists and pharmacy technicians?
2. Is there a difference in mean NEP scores between UK pharmacists and pharmacy technicians?
3. Is there a difference in mean NEP scores between Australian and UK participants?

#### Comparison of mean NEP scores between Australian pharmacists and pharmacy technicians

In the sample of 66 Australian participants, 65.2% (43/66) were pharmacists and 34.8% (23/66) were pharmacy technicians. The mean NEP score for the sample was 54.58 (sd = 6.906). The mean NEP score for pharmacists was 54.65 (sd = 7.33; 95% CI: 52.39–56.91) and mean NEP score for pharmacy technicians was 54.54 (sd = 6.19; 95% CI: 51.76–57.11). An independent two-sample t-test was conducted to compare mean NEP scores of each group. This test has three assumptions each of which was checked. The first assumption is that data are normally distributed–this was checked and affirmed. The second assumption is that the two samples are independent–the study design ensured this. For the third assumption (equal variances) Levene’s test was conducted (F = 2.941, p = 0.089) and equal variances was assumed. Therefore, all three assumptions of this test were met. There was no statistically significant difference in mean NEP scores between Australian pharmacists and pharmacy technicians in the sample (t_104_ = -0.796, p = 0.428). On average, Australian pharmacy technicians had a mean NEP score 1.04 points higher than pharmacists (95% CI: -3.63–1.55).

#### Comparison of mean NEP scores between UK pharmacists and pharmacy technicians

In the sample of 40 UK participants, 57.5% (23/40) were pharmacists and 42.5% (17/40) were pharmacy technicians. The mean NEP score for the sample was 54.10 (sd = 6.04). The mean NEP score for pharmacists was 52.87 (sd = 6.884; 95% CI: 49.89–55.85) and the mean NEP score for pharmacy technicians was 55.76 (sd = 4.323; 95% CI: 53.54–57.99). An independent two-sample t-test was conducted to compare the mean NEP scores of each group. This test has three assumptions each of which was checked. The first assumption is that data are normally distributed–this was checked and affirmed. The second assumption is that the two samples are independent–the study design ensured this. For the third assumption (equal variances) Levene’s test was conducted, and equal variances were assumed for pharmacists and pharmacy technicians (F = 3.895, p = 0.056). There was no statistically significant difference in mean NEP scores between pharmacists and pharmacy technicians in this sample (t_38_ = -1.523, p = 0.136). On average, UK pharmacy technicians had a mean NEP score 2.895 points higher than pharmacists (95% CI: -6.74–0.95).

#### Comparison of mean NEP scores between Australian and UK participants

Of the combined sample of 106 participants, 62.5% (66/106) were Australian and 37.7% (40/106) were from the UK. The mean NEP score for this sample was 54.4 (sd = 6.57). The mean NEP score of the Australian cohort was 54.58 (sd = 6.91; 95% CI: 52.88–56.27) and the mean NEP score of the UK cohort was 54.10 (sd = 6.04; 95% CI: 52.17–56.03). An independent sample t-test was then undertaken to determine if there was a difference in mean NEP scores between Australian participants and UK participants, with p<0.05 used to indicate statistical significance. Equal variances in NEP scores were checked using Levene’s test and equal variances were assumed for Australian and UK participants (F = 0.135, p = 0.714). There was no statistically significant difference in mean NEP scores between the two groups in this sample (t_104_ = 0.360, p = 0.720). On average Australian participants had a mean NEP score 0.476 point higher than the UK cohort (95% CI: -2.15–3.10).

Of the 106 participants in the study, 15 Australians (22.73% of the Australian sample) and eight UK (20% of the UK sample) scored in the pro-environmental range (NEP score > 59). The remainder scored in the mid-environmental range (NEP score 39–58).

### Environmental knowledge

#### Knowledge of issue

Knowledge of the issue was explored by asking participants, “What do you think are the real concerns regarding pharmaceuticals entering the environment?”. Participants’ responses were uploaded into Leximancer® and an Insight Dashboard report including Quadrant graphic (S1 Fig) was generated. Prominent concepts for each cohort are listed in Table 3.

**Table 3:** Comparison of environmental knowledge between Australian and UK cohorts–knowledge of issue.

*What do you think are the real concerns with pharmaceuticals entering the natural environment*?	
Australian Cohort	UK Cohort
***Leximancer® Insight Dashboard—Quadrant Prominent Concepts***	
Landfill	Water
Plants	Resistance
Sink	Wildlife
People	Animals
***Manual Coding Themes***	
Impacts on ecological systems	Impacts on ecological systems
Public health concerns	Public health concerns
Lack of environmental knowledge/problem awareness	Lack of environmental knowledge/problem awareness
	Intrinsic motivation

The most prominent concepts in the Australian dataset were ‘landfill’, ‘plants’, ‘sink’ and ‘people’. They referred to inappropriate disposal of pharmaceuticals e.g. via sinks, and pharmaceuticals appearing in landfill, waterways, and soil thus impacting plants and people.

“*Even throwing it out in landfill it’s going to be leached out and end up back in waterways…*..*” [D084]*

“*So if we’re putting anything down a sink we’re going to destroy our rivers*, *if we’re putting stuff in landfill it’s just going to sit there forever and ever and ever*.*” [S211]*

The concepts ‘water’ and ‘animals’, and ‘wildlife’ were much less prominent in the Australian dataset compared with the UK dataset. The most prominent concepts in the UK dataset were ‘water’, ‘resistance’, ‘wildlife’, and ‘animals’. UK participants mentioned pharmaceuticals entering waterways and thereby reaching flora, fauna, and humans as highlighted by the following examples from the interviews.

“*Like animals and obviously crops and things like that and soil and the water and things like that*.*”* [J142]

“*Hazardous to the soil which can affect plants and stop their growth*. *…… I guess if there’s remnants of any kind of drug left and they’re put into the soil then they can harm water that you drink et cetera and the soil*.*”* [P061]

Of note was the low prominence of the concept ‘people’ (relative to other prominent concepts) in the Australian dataset and its absence in the UK dataset. This indicates that the connection between pharmaceuticals’ entry into the environment (with subsequent entry into food chains and water cycles), and ultimately into humans was not made very often with either cohort. Australian participants were more concerned about inappropriate disposal down sinks and in general rubbish destined for landfill compared with their UK counterparts as seen in this response from an Australian participant:

“*……*.. *harm to natural flora and fauna*, *bacteria and a lot of these agents are toxic–well they’re designed for people–just by tossing out antibiotics whether it’s down the sink or in general waste you run the risk of developing resistance*. *If medicines end up in landfill–we all know that birds and rats and everything will be eating the waste*.*”* [R223]

The Leximancer® analysis therefore highlighted a lack of understanding of the connection between the environment/ecosystems and human health in both cohorts and this was seen in the manual coding process with the emergence of the theme, ‘lack of environmental knowledge/problem awareness’. The manual coding trees for the Australian and UK participants’ responses to this question are provided in the supplementary materials (S2 and S3 Figs respectively).

In the manual coding process, a category unique to the UK cohort emerged—‘barriers to PEBs’. This category was synthesised from the codes ‘issue denial’, ‘nature will adapt’, ‘unconcerned enough to act’, and ‘unconcerned because never thought of issue before’ and was one of the categories merged into the theme ‘lack of environmental knowledge/problem awareness.

“*I think in this country it’s okay*. *Because I don’t think we just go around dumping it in landfill …”* [R141]

“*However*, *I do believe that nature itself will adapt to excipients going into the natural environment*.*”* [C301]

**“***I’m not going to lie*, *it doesn’t take up a lot of my daily thought processes*, *not a lot*. *Because I’m sure everyone has got their own role in the world*. *I’m sure there’s somebody dealing with that side of things as well*.*”* [R101]

#### Knowledge of action strategy

This was explored by asking participants six questions (Table 4).

**Table 4:** Comparison of environmental knowledge between Australian and UK cohorts–knowledge of action strategies.

Australian Cohort			UK Cohort	
*How do you dispose of unwanted pharmaceuticals*?				
***Leximancer® Themes***				
Organisational factors			Organisational factors	
Environmental regulations			Responsibility	
			Education and training	
***Manual Coding Themes***				
Impacts on ecological systems			Impacts on ecological systems	
Public health concerns			Public health concerns	
Environmental knowledge/prob. awareness			Environmental knowledge/prob. awareness	
			Intrinsic motivation	
*How do you dispose of original containers and non-contaminated packaging waste*?				
***Leximancer® Themes***				
Bin			Recycling	
General			Confidential	
Confidential			General	
Packaging			Cardboard	
Recyclable			Paper	
			Packaging	
***Manual Coding Themes***				
Organisational factors			Trust responsibility	
			Individual responsibility	
***The following questions generated quantitative data*:**				
**Response**	**Australian Participants (n = 64)**	**UK Participants (n = 40)**		**Total (n = 104)**
*Do you know what happens to pharmaceutical waste after it leaves the pharmacy department*?				
**Yes**	12 (18.75%)	5 (12.5%)		16.35%)
*Do you know what happens to pharmaceutical waste after it leaves the hospital*?				
**Yes**	19 (29.69%)	1 (2.5%)		19.23%)
*Do you know what constitutes best practice for the environmentally responsible disposal of pharmaceutical waste*?				
**Yes**	8 (12.5%)	2 (5%)		9.62%)
*Do you know where to find best practice guidelines for the environmentally responsible handling of pharmaceutical waste in this department*?				
**Yes**	5 (7.81%)	15 (37.5%)		20 (19.23%)

Firstly, participants were asked how they disposed of unwanted pharmaceuticals. The Australian pharmacists and pharmacy technicians all reported disposing of this waste stream in the yellow, clinical waste bins (Leximancer® concept map–S4 Fig). Interestingly, the UK pharmacists reported that as they did not dispense pharmaceutical waste was not their responsibility and was handled by the pharmacy technicians. The UK pharmacy technicians reported disposing in the clinical waste bin (Leximancer® concept map–S5 Fig). Similar themes emerged for both cohorts with the key common theme being that participants reported using the appropriate bins to comply with departmental and hospital/Trust policy. Environmental waste management bin audits, with results reported back to departmental heads, reinforced compliance in both cohorts rather than environmental knowledge, environmental concern, or pro-environmental attitude.

Next, participants were asked how they disposed of non-contaminated packaging waste. The Leximancer® Quadrant graphic of the responses to this question (S6 Fig) clearly indicates Australian participants reported disposing of non-contaminated paper and packaging waste in general waste bins (landfill). Across the five Australian hospitals there were different policies for handling non-contaminated waste with patient identifiers on it (confidential waste). Some hospitals blacked out patient details on packaging waste which was then disposed of in general waste bins for landfill whereas others had a shredding bin for confidential waste (either shredded on-site and sold to external contractors or external contractor paid to remove for shredding off-site).

“*…*.. *We don’t really have any recycling bins around*.*”* [C174]

“*Packaging waste just goes in the normal rubbish bin*, *if it doesn’t have a patient’s label on it*.*”* [L032]

The most prominent concepts in the UK data set were about recycling (S6 Fig). This encompassed comments about the particular trust offering recycling, or no recycling bins being available, or recycling not being done very well. In those hospitals with no recycling bins, this waste stream went into general waste destined for landfill.

“*I don’t think we’re very good with packaging*, *putting those in the recycling boxes*. *I think they tend just to go in a black [general waste] bin*.*”* [A231]

“*Well I would like to recycle it but there’s not the option…*.. *So*, *it just goes in the normal bin …and general landfill*.*”* [C071]

Manual coding produced four themes for the Australian data (Manual coding tree–S7 Fig) and three themes for the UK data (Manual coding tree–S8 Fig) (Table 4). Both cohorts reported either a lack of recycling bins or inconvenient locations as a barrier to recycling non-contaminated recyclable waste. Australian legislation covering a levy imposed on organisations for waste disposal either by incineration or landfill [47], incentivised recycling of original containers and packaging waste in locations with recycling bins. In Australian and UK hospitals where recycling bins were available, many staff still reported disposing of recyclable non-contaminated packaging waste in general bins destined for landfill. Therefore, very similar behaviours were evident across both cohorts. Again, all the UK pharmacists who worked predominantly on the wards commented that because they did not dispense, pharmaceutical waste was not their responsibility. When asked about the bins on the wards they were able to report that nursing staff disposed of pharmaceutical waste in yellow, clinical waste bins if it could not be returned to pharmacy. Some of them were quite vague as to the appropriate disposal of contaminated pharmaceutical waste.

“*We don’t really have that much involvement with the waste side of things…”* [R101]

A lack of recycling bins in the main dispensary work areas and a lack of recycling policies and procedures regarding recycling non-contaminated, recyclable packaging waste were the key organisational factors highlighted by both Australian and UK participants. UK participants predominantly believed it was the Trust’s responsibility to provide appropriate infrastructure with a couple reporting that individuals took responsibility for obtaining appropriate recycling bins for their departments.

“*…*. *we chuck it in the general bin but all of the non-contaminated pharmaceutical waste is probably recyclable*.*”* [R223]

“*We’re not provided with … recycling bins*. *There’s a lot of paper that doesn’t need to go in the confidential Shreddex bin that goes into the general waste bin because we’re told not to overload the Shreddex bin with unnecessary things because we pay for it*.*”* [T221]

“*It’s reasonably easy*, *but I think that’s because some people have taken action to make it easier*. *So*, *we do have recycling bins in our office but that’s because somebody requested that we have recycling bins in our office*.*”* [T232]

Participants were next asked four questions which elicited Yes/No responses (Table 4). Results indicated the majority of participants did not know the fate of pharmaceutical waste once it left their department, nor best practice disposal methods (although most thought it was high temperature incineration which is the appropriate disposal method).

#### Environmental concern

The levels of concern regarding the impact of pharmaceuticals entering the environment were then compared between Australian and UK participants (Table 5) (S4 File).

**Table 5:** The distribution of participants’ responses to the question, “How concerned are you personally about pharmaceuticals entering the natural environment?”–environmental concern.

Response	Australia (n = 64*)	UK (n = 40)	Total Combined (n = 104)
Concerned	36 (56.25%)	16 (40%)	52 (50%)
Not concerned	28 (43.75%)	24 (60%)	52 (50%)

*Data missing for two Australian participants.

Next, the research question, “Is the proportion of people reporting environmental concern the same or different for Australian and UK participants?” was examined. The sample comprised 61.54% (64/104) Australian participants and 38.46% (40/104) UK participants of whom 49.1% (52/104) expressed concern for pharmaceuticals entering the natural environment. A higher percentage of Australian participants (56.25%; 36/64) compared with UK participants (40%; 16/40) reported feeling concerned about the impact of pharmaceuticals entering the environment (Table 5). Fisher’s Exact Test was used to determine if there was a statistically significant difference in environmental concern between Australian and UK participants and no difference was found (Fisher’s Exact Test, p = 0.158). For both cohorts, there was no statistically significant association between a participant’s level of environment concern (‘concerned’ versus ‘not concerned’) and their role (pharmacist or pharmacy technician), operational level, gender, or the region/city in which they worked (refer to S4 File for details of statistical tests).

Next, binary logistic regression was performed to determine if environmental attitude (NEP score) was a predictor of environmental concern in each cohort (S4 File). Environmental attitude was only a statistically significant predictor of environmental concern in the Australian cohort ($\chi_{1}^{2}$ = 4.530, p = 0.033, OR = 0.92). The model explained between 6.6% (Cox and Snell R Square) and 8.9% (Nagelkerke R Square) of variance in environmental concern.

## Discussion

The principal finding of this research was that regardless of the presence or absence of carbon reduction legislation there was very little difference in pharmaceutical waste disposal practices between Australian and UK hospital pharmacy staff. The UK has carbon emissions reduction legislation in place, a government committed to reducing the country’s carbon emissions, and a public health system with a specialised unit dedicated to developing and implementing a carbon reduction strategy. Unfortunately, this was not evident from the responses of the UK pharmacists and pharmacy technicians in this research. UK Government and NHS sustainability policy did not appear to have disseminated to the pharmacy department level of UK public hospitals to any great extent.

Many researchers have found the link between environmental attitudes and behaviours to be weak [48–50], including Blake who proposed the ‘Value-Action-Gap’ (the gap between environmental attitudes and environmental action) [51]. However, other researchers have found environmental attitudes to have a significant effect on PEBs [52–55]. Several models of PEBs argue that environmental knowledge/awareness is a predictor of environmental attitude and PEBs [29, 33, 56–58]. However, others argue that whilst increased environmental knowledge can lead to awareness of environmental issues and raised levels of environmental concern, it still may not lead to PEBs [33, 59, 60].

This study supports Blake’s findings–in each cohort approximately 20% of participants were pro-environmental; however, a pro-environmental attitude did not lead to increased workplace PEBs. Despite a pro-environmental attitude, these participants had low levels of environmental knowledge regarding the impact of pharmaceuticals on the environment and most reported low levels of environmental concern. Both Australian and UK pharmacy staff reported pharmaceutical waste disposal as not being a priority in the hospital pharmacy workplace; competing workplace pressures contributed to a gap between environmental values and actual workplace PEBs. This highlights an organisational barrier and provides insight into why pro-environmental staff might lack motivation to overcome another identified organisational barrier (lack of recycling bins) to workplace PEBs.

Through the use of mixed methods, qualitative and quantitative data could be compared for each participant and the circular relationship between environmental attitude, knowledge, and concern explored. Regardless of environmental attitude (NEP score), all participants demonstrated a lack of knowledge of the impacts of pharmaceutical waste on the environment (direct chemical effects and carbon footprint) and this carried through into low levels of environmental concern. Only in the Australian cohort was environmental attitude a predictor of environmental concern. Those who expressed concern had not sought further to increase their environmental knowledge. This lack of concern in the majority of participants could be due to a lack of understanding of the interconnectedness between the environment and human health and the environmental determinants of human health. In both cohorts, most participants reported they had never given much thought previously to the impact of pharmaceuticals on the environment and the subsequent negative impacts on human health. In the UK cohort, issue denial or a belief that nature can adapt to pharmaceuticals entering the environment was also linked to a lack of environmental concern. For the majority of participants who had a mid-environmental attitude, low environmental concern, and low environmental knowledge, there was minimal engagement with PEBs as predicted by the conceptual model (Fig 1). The findings appear to indicate that environmental attitude, knowledge, and concern are weaker influences and workplace social norms a stronger influence on workplace PEBs than the model predicted. Workplace social norms as antecedents of workplace PEBs warrant future exploration since compliance with hospital policies for audited clinical waste streams indicates that workplace norms are at play in this context.

Barriers to workplace PEBs as listed in the model (Fig 1), such as lack of infrastructure, were seen in this study with a key barrier identified by both Australian and UK participants as a lack of recycling bins in the main dispensary work areas and a lack of a policy and procedure for recycling non-contaminated, recyclable packaging waste. Interestingly, another barrier identified was abnegation of responsibility. UK pharmacists in this study did not believe waste to be in the scope of their role and therefore not their responsibility. This corroborates the findings of Tudor *et al*. [61] in their research into waste management practices in the Cornwall Trust of the NHS England. This was an interesting difference between the Australian and UK pharmacists. However, according to FIP, pharmacists *do* have a responsibility for the environmental impact of medicines [62]. In 2015, FIP released its guideline ‘Green Pharmacy Practice’ to help pharmacists consider the environmental implications of their professional practice [62]. That pharmacists in Australia and UK continue to remain unaware or have never considered the environmental impact of pharmaceuticals on the environment would appear to indicate that policies and undertakings of the profession’s international body are not being taken up by national pharmacy organisations or being disseminated to grassroots members. For the profession going forward, this lack of awareness will need to be addressed to help combat current global public health challenges such as antimicrobial resistance [63] and reduced human fertility [64]. The International Pharmaceutical Federation (FIP) has identified that integral to pharmacists playing a role in these global public health challenges is the adoption of the ‘One Health’ approach [65] “which recognises the interconnectedness of the health of humans, animals, and ecosystems. This should be integrated into pharmacy degree curricula and included in continuing professional development activities for registered pharmacists. If the pharmacy profession is to contribute meaningfully in the future to reducing the carbon footprint of healthcare delivery these identified policy failures warrant further research.

The authors acknowledge that a limitation with this research is the small number of hospitals recruited in each country. A larger number of hospitals across multiple Australian cities and across the UK including NHS Trusts in Scotland, Wales, and Northern Ireland would have strengthened the findings; however, this was outside the scope of the research both financially and in time available.

## Supporting information

S1 Fig Leximancer® insight dashboard quadrant graphic interview question 8. (PDF)

S2 Fig Manual coding tree Australian cohort interview question 8. (JPG)

S3 Fig Manual coding tree UK cohort interview question 8. (JPG)

S4 Fig Leximancer® concept map Australian cohort interview question 2. (JPEG)

S5 Fig Leximancer® concept map UK cohort interview question 2. (PNG)

S6 Fig Leximancer® insight dashboard quadrant graphic interview question 3. (PDF)

S7 Fig Manual coding tree Australian cohort interview question 3. (JPG)

S8 Fig Manual coding tree UK cohort interview question 3. (JPG)

S1 File New Ecological Paradigm (NEP) Scale. (PDF)

S2 File Interview questions. (PDF)

S3 File Leximancer® information. (PDF)

S4 File Statistical testing analytical plans. (PDF)

S5 File Raw data file (Excel) exported from SPSS. (XLSX)

S6 File SPSS coding manual. (DOCX)
