# Supplementary figures and images for: Do legislated carbon reduction targets influence pro-environmental behaviours in public hospital pharmacy departments? Using mixed methods to compare Australia and the UK
Source: PLoS One. 2021 Aug 18;16(8):e0255445. doi: 10.1371/journal.pone.0255445 (PMC8372918; doi:10.1371/journal.pone.0255445)

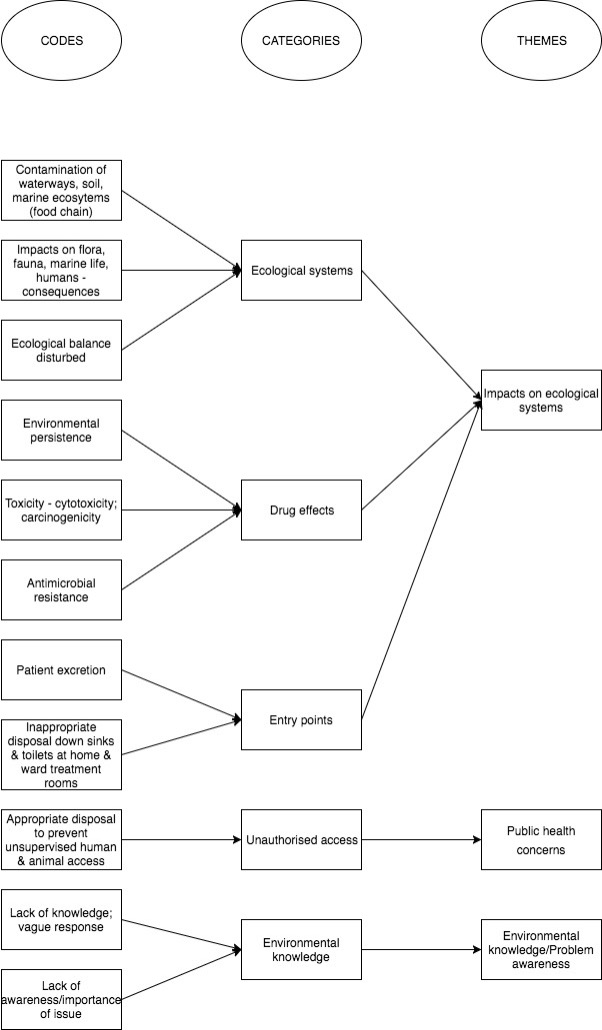

Supplement: S2 Fig — (JPG) [file pone.0255445.s002.jpg]

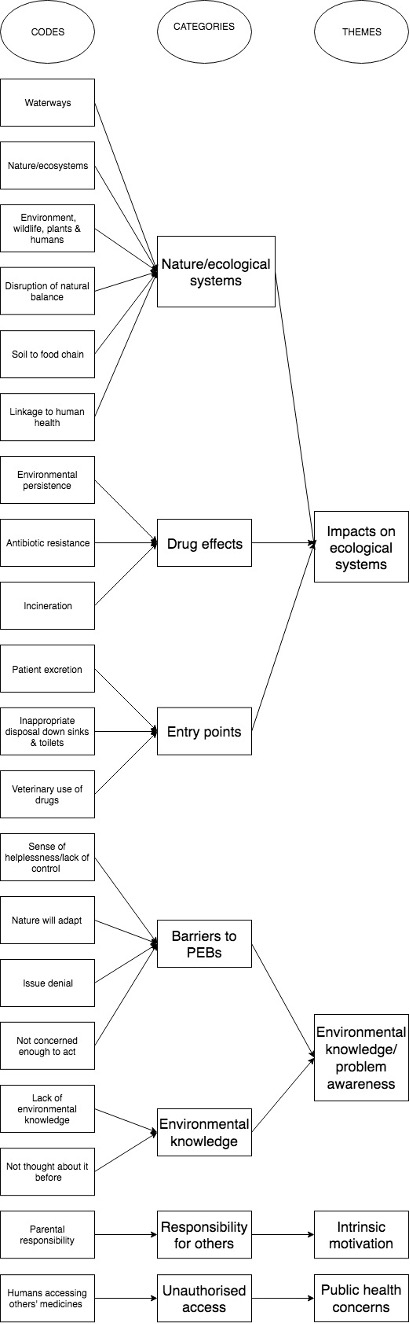

Supplement: S3 Fig — (JPG) [file pone.0255445.s003.jpg]

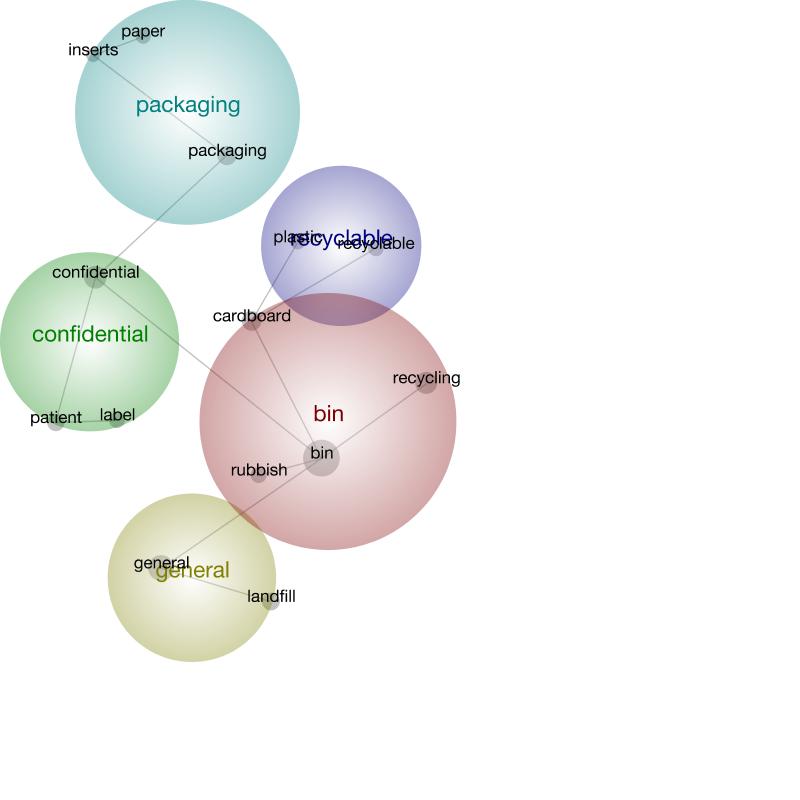

Supplement: S4 Fig — (JPEG) [file pone.0255445.s004.jpeg]

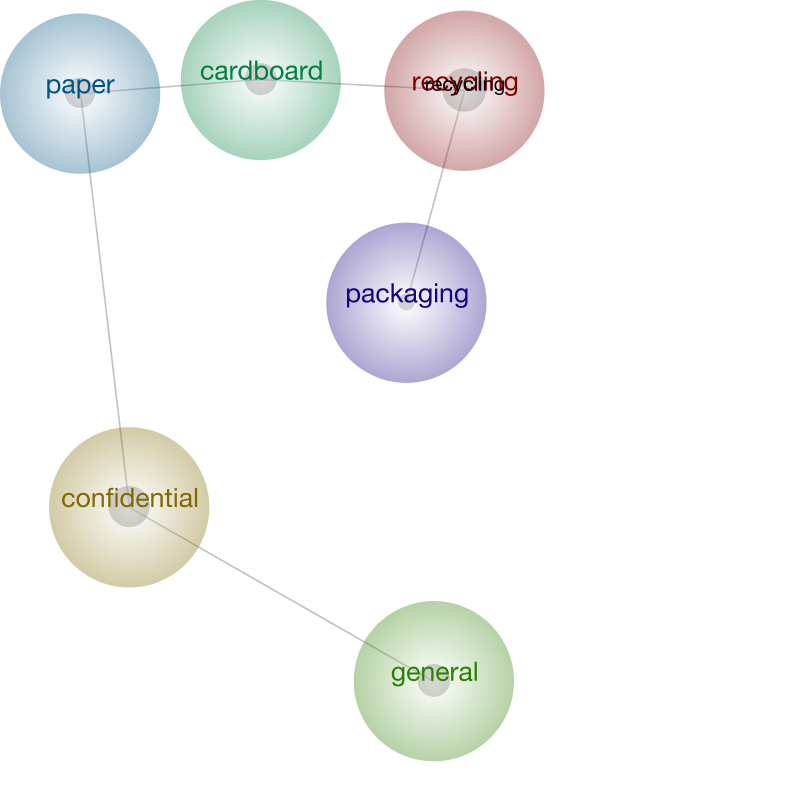

Supplement: S5 Fig — (PNG) [file pone.0255445.s005.png]

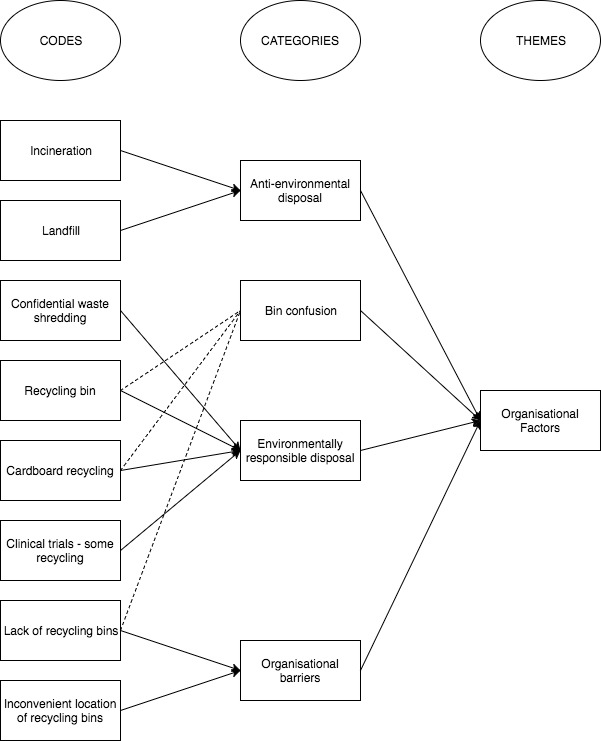

Supplement: S7 Fig — (JPG) [file pone.0255445.s007.jpg]

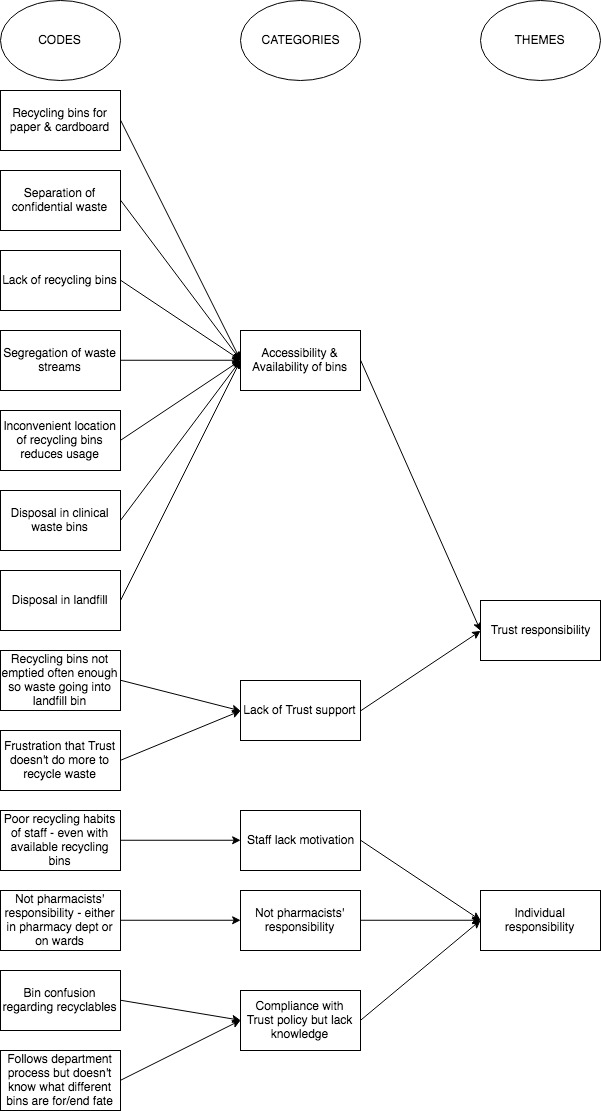

Supplement: S8 Fig — (JPG) [file pone.0255445.s008.jpg]
