# Supplementary material for: Do legislated carbon reduction targets influence pro-environmental behaviours in public hospital pharmacy departments? Using mixed methods to compare Australia and the UK
Source: PLoS One. 2021 Aug 18;16(8):e0255445. doi: 10.1371/journal.pone.0255445 (PMC8372918; doi:10.1371/journal.pone.0255445)
Supplement: S6 Fig — (PDF) [file pone.0255445.s006.pdf]

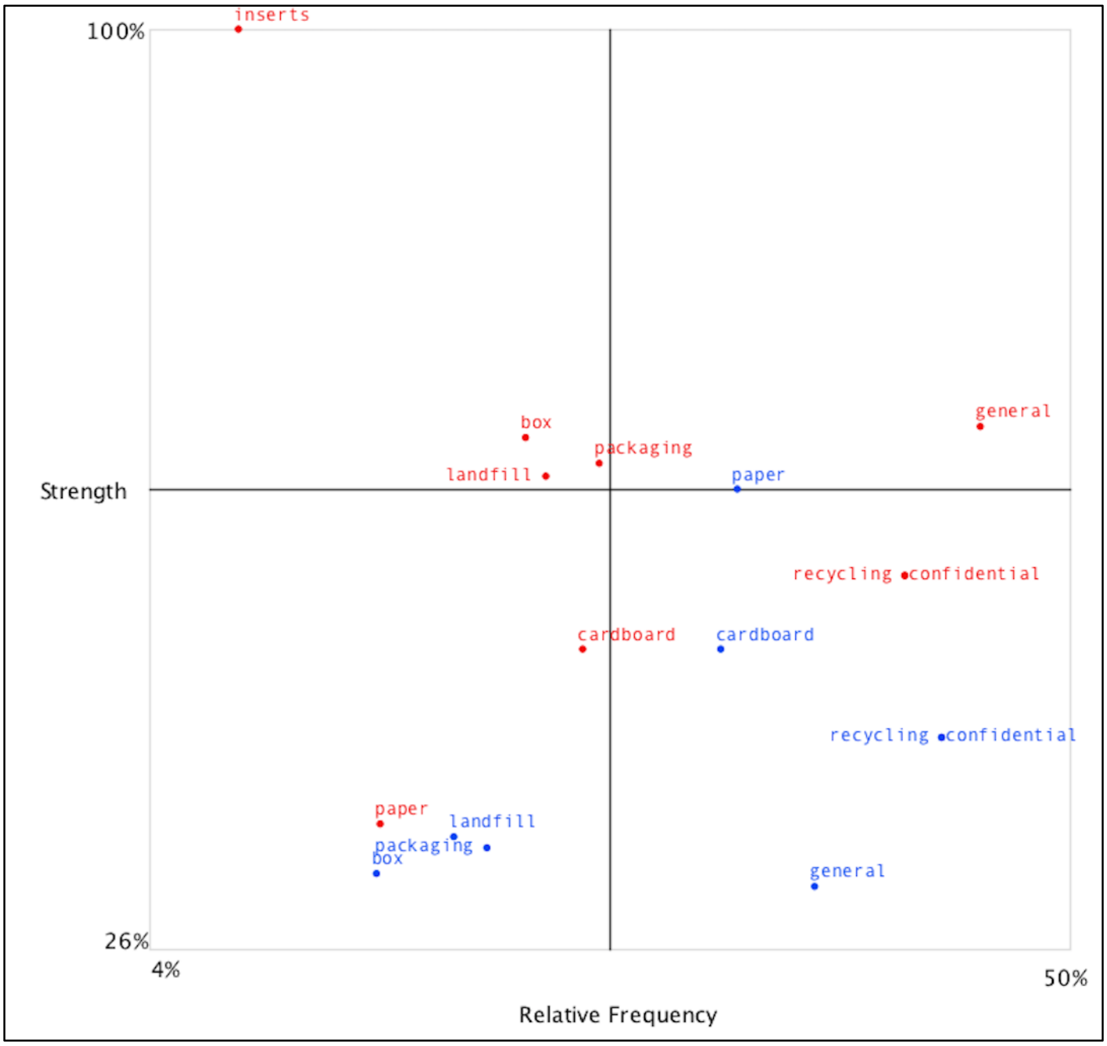

S6 Fig. Leximancer® Quadrant Overview of Australian (red) and English (blue) Participants' Disposal of Original Containers and Non-Contaminated Packaging Waste
