# Supplementary material for: Do legislated carbon reduction targets influence pro-environmental behaviours in public hospital pharmacy departments? Using mixed methods to compare Australia and the UK
Source: PLoS One. 2021 Aug 18;16(8):e0255445. doi: 10.1371/journal.pone.0255445 (PMC8372918; doi:10.1371/journal.pone.0255445)
Supplement: S1 File — (PDF) [file pone.0255445.s009.pdf]

New Ecological Paradigm (NEP) Scale Items (Dunlap, Van Liere, Mertig, & Jones, 2000)

I would like you to place a tick in the box for the response you mostly closely align with for each of the following statements.
SA – strongly agree
A – agree
N – neutral
D – disagree
SD – strongly disagree

**Statement**	SA	A	N	D	SD
1. We are approaching the limit of the number of people the earth can support.					
2. Humans have the right to modify the natural environment to suit their needs.					
3. When humans interfere with nature it often produces disastrous consequences.					
4. Human ingenuity will ensure that we do NOT make the earth unliveable.					
5. Humans are severely abusing the environment.					
6. The earth has plenty of natural resources if we just learn how to develop them.					
7. Plants and animals have as much right as humans to exist.					
8. The balance of nature is strong enough to cope with the impacts of modern industrial nations.					
9. Despite our special abilities, humans are still subject to the laws of nature.					
10. The so-called 'ecological crisis' facing humankind has been greatly exaggerated.					
11. The earth is like a spaceship with very limited room and resources.					
12. Humans were meant to rule over the rest of nature.					
13. The balance of nature is very delicate and easily upset.					
14. Humans will eventually learn enough about how nature works to be able to control it.					
15. If things continue on their present course we will soon experience a major ecological catastrophe.					

Scoring

Dunlap and Van Liere [182] described the design of the 15-item NEP Scale as follows, "Three items were designed to tap each of the five hypothesized facets of an ecological worldview: the reality of limits to growth (1,6,11), anti-anthropocentrism (2,7,12), the fragility of nature's balance (3,8,13), rejection of exemptionalism (4,9,14), and the possibility of an ecocrisis (5,10,15)."[182, p. 432]

The eight odd-numbered items on the scale were worded in such a way that agreement demonstrates a pro-ecological worldview. The seven even-numbered items on the scale were worded in such a way that disagreement demonstrates a pro-ecological worldview.[182] For this research, the NEP Scale was scored as follows:

The eight odd-numbered items:

Strongly Agree	Agree	Neutral	Disagree	Strongly Disagree
5 points	4 points	3 points	2 points	1 point

The seven even-numbered items:

Strongly Agree	Agree	Neutral	Disagree	Strongly Disagree
1 point	2 points	3 points	4 points	5 points

Scores were allocated into the categories of pro-environmental, mid-environmental and anti-environmental.

Anti-environmental: Scores in the range 0 - 38 which are equivalent to percentage scores in the range 0% - 51%

Mid-environmental: Scores in the range 39 - 58 which are equivalent to percentage scores in the range 52% - 77%

Pro-environmental: Scores in the range 59 -75 which are equivalent to a percentage score ≥ 78%

Reference

Dunlap, R. E., Van Liere, K. D., Mertig, A. G., & Jones, R. E. (2000). New Trends in Measuring Environmental Attitudes: Measuring Endorsement of the New Ecological Paradigm: A Revised NEP Scale. *Journal of Social Issues, 56*(3), 425-442. doi: 10.1111/0022-4537.00176
