# Supplementary material for: Do legislated carbon reduction targets influence pro-environmental behaviours in public hospital pharmacy departments? Using mixed methods to compare Australia and the UK
Source: PLoS One. 2021 Aug 18;16(8):e0255445. doi: 10.1371/journal.pone.0255445 (PMC8372918; doi:10.1371/journal.pone.0255445)
Supplement: S2 File — (PDF) [file pone.0255445.s010.pdf]

Phase IA Questions

Definitions for the purposes of this research

Workplace Pro-environmental Behaviours: *'actions and behaviours that employees consciously engage in to minimise the negative impact on the natural and built world'*

Pharmaceutical Waste: *'Pharmaceutical waste refers to pharmaceuticals and their original containers (contaminated waste component) and the packaging waste (non-contaminated component)'*

Questions

1. In your workplace are there systems or departmental procedures for the disposal of pharmaceutical waste? In the dispensing area? In the distribution/supply area?

2. How do you dispose of unwanted pharmaceuticals?

3. How do you dispose of original containers and non-contaminated pharmaceutical packaging waste?

4. Do you know what happens to pharmaceutical waste after it leaves the pharmacy department?

5. Do you know what happens to pharmaceutical waste after it leaves the hospital?

6. Do you know what constitutes best practice for the environmentally responsible disposal of pharmaceutical waste?

7. Do you know where to find best practice guidelines for the environmentally responsible handling of pharmaceutical waste? [If they respond with Qld Health policies, ask if they know what these guidelines are referenced against.]

8. What do you think are the real concerns around pharmaceuticals entering the environment?

9. How concerned are you personally about pharmaceuticals entering the natural environment?

10. I would now like to ask you a question about green suppliers. By this term I mean drug companies who are members of the Australian Packaging Covenant and have undertaken to reduce the amount of packaging waste produced and ensure that the packaging waste that is produced is recyclable. I also include drug companies who are actively seeking to reduce the carbon footprint of their manufacturing and distribution operations.
If this department were able to control all pharmaceutical purchases for the hospital, would you recommend the department change to greener suppliers (drug

companies) a) if there was no extra cost? b) if the greener suppliers were more expensive?

11. Do you engage in pro-environmental behaviours at home? If yes: “Can you list some of these behaviours?” [Hint: recycling, energy and/or water conservation]

12. Many people obtain a great deal of personal satisfaction from engaging in pro-environmental behaviours in the home and in the workplace. How satisfied do you feel when you are able to engage in pro-environmental behaviours in your workplace? Rate your answer on a scale of 1 to 5 where 1 is very dissatisfied, 3 is neutral and 5 is very satisfied.

13. Organisational structures like hospitals aren’t always designed in a way that easily supports workplace pro-environmental behaviours. In this department how easy do you feel it is to treat pharmaceutical waste (both contaminated and non-contaminated components) in an environmentally responsible manner?
