# Supplementary material for: Do legislated carbon reduction targets influence pro-environmental behaviours in public hospital pharmacy departments? Using mixed methods to compare Australia and the UK
Source: PLoS One. 2021 Aug 18;16(8):e0255445. doi: 10.1371/journal.pone.0255445 (PMC8372918; doi:10.1371/journal.pone.0255445)
Supplement: S3 File — (PDF) [file pone.0255445.s011.pdf]

Leximancer Information

Leximancer® analyses text by converting lexical co-occurrence information from natural language into semantic patterns in an automated manner. "It employs two stages of co-occurrence information extraction – semantic and relational – using a different algorithm for each stage. The algorithms used are statistical, but they employ non-linear dynamics and machine learning".[1, 2 p. 262] Leximancer® was used to analyse the content of the text and display the extracted information visually as a concept map. Thus, Leximancer® provided a means of quantifying and displaying the conceptual structure of the text, enabling an exploration of the relationships between the identified concepts. An automated text analysis tool such as Leximancer® eliminates the 'human error' component whereby human decision makers may be affected by influences they are unable to report.[2] However, running texts through the Leximancer® software involves an editing process in which the researcher may eliminate words such as articles and pronouns (due to their high occurrence without adding to semantic content). This arguably does allow some subjectivity on the part of the researcher to enter the data analysis process. However, qualitative research acknowledges that the subjectivity of the researcher's analysis of the data is in itself an important part of the qualitative research process.

Concepts in Leximancer® are collections of words that generally appear together throughout the text. The concept map provides a bird's eye view of the data, depicting the main concepts and how they interrelate. Concepts that appear often together in the text are clustered together into themes that are heat mapped - the hotter the colour, the more important the theme with red being the hottest, then orange, and so on as per the colour wheel. For each question, the concept map derived from Leximancer® was examined to determine the main themes. The entire process was run several times to check that the relative positioning of the concepts was similar between runs. This confirms that the concept map is representative of the data set.

Another function of Leximancer® is the Insight Dashboard report which can be produced inside a project.[3] This report is designed for some type of comparison or differential analysis and therefore was ideal for comparing Australian participants' responses with the English participants' responses for identical questions. The Insight Dashboard report is used to investigate the Attributes (independent variables) associated with certain Categories (dependent variables).[3] It "adopts a more quantitative focus than the concept map, and is designed to provide a quick understanding of the project results." [3, p.100] The Australian participants' responses for each question were tagged as one category and the English participants' responses to the same question were tagged as a second category. The Dashboard quadrant overview map displays the concepts on a quadrant with the upper right-hand quadrant described as the 'magic' quadrant – concepts in this quadrant are those talked about most frequently and are the most characteristic for the text.[3]

The Y-axis or 'Relative Frequency' on the Quadrant graphic represents the conditional probability that an Attribute (concept) is coded in this particular dataset. It measures the frequency with which a concept is mentioned in the text and is affected by the distribution of comments across the categories. The frequency score is in fact a log scale to enable it to be plotted on the quadrant.[3] The X-axis or Strength score is the reciprocal conditional probability. Given that an Attribute or concept is present in the text, this score represents the probability

that the particular text belongs to a particular Category.[3] In this research the two Categories are the Australian interview data and the English interview data.

The Relative Frequency scores and the Strength scores for each concept are then combined to produce a 'Prominence' score – the Quadrant coordinates. Prominence scores are absolute measures of the correlation between an Attribute (concept) and the Category. Leximancer® also generates a 'Prominence' score for concept pairs (two concepts that frequently co-occur in text) and categories. This score combines the 'Strength' and 'Frequency' scores using Bayesian statistics and is an absolute measure of correlation between category (Australian or English data sets) and concept pairs.[3]

References

1. Smith AE and Humphreys MS. Evaluation of unsupervised semantic mapping of natural language with Leximancer concept mapping. *Behavior Research Methods* 2006; 38: 262-279. DOI: 10.3758/bf03192778.
2. Leximancer. Leximancer Manual Version 4.5. University of Queensland, Brisbane, 2017.
