# Supplementary material for: Do legislated carbon reduction targets influence pro-environmental behaviours in public hospital pharmacy departments? Using mixed methods to compare Australia and the UK
Source: PLoS One. 2021 Aug 18;16(8):e0255445. doi: 10.1371/journal.pone.0255445 (PMC8372918; doi:10.1371/journal.pone.0255445)
Supplement: S4 File — (PDF) [file pone.0255445.s012.pdf]

S4_Doc Analytical Plans for SPSS Analyses

Title: 'Do legislated carbon reduction targets influence pro-environmental behaviours in public hospital pharmacy departments? Using mixed methods to compare Australia and the UK.'

Table of Contents

Reliability of NEP Scale for Australian Sample 3

Reliability of NEP Scale for UK Sample 4

Research Question: "Is there a difference in mean NEP score between Australian pharmacists and pharmacy technicians?" 5

1- Analytical Plan 5

2 – Output of analysis 6

3 – Summary and Interpretation of Analysis Results 11

Research Question: "Is there a difference in mean NEP score between UK pharmacists and pharmacy technicians?" 12

1- Analytical Plan 12

2 – Output of Analysis 13

3 – Summary and Interpretation of Analysis Results 17

Research Question: Is there a difference in mean NEP scores between Australian and UK participants? 18

1- Analytical Plan 18

2 – Output of Analysis 19

3 – Summary and Interpretation of Analysis Results 23

Research Question: Is there a difference in level of environmental concern between Australian and UK participants? 24

1- Analytical Plan 24

2 – Output of Analysis 25

3 – Summary and Interpretation of Analysis Results 28

Research Question: Is there an association between Australian participants' role (pharmacist or pharmacy technician) and their level of environmental concern? 29

1- Analytical Plan 29

2 – Output of Analysis 30

3 – Summary and Interpretation of Analysis Results 34

Research Question: Is there an association between Australian participants' operational level and their level of environmental concern? 35

1- Analytical Plan 35

2- Output of Analysis 36

3 – Summary and Interpretation of Analysis Results 38

Research Question: Is there an association between Australian participants' gender and their level of environmental concern? 40

1- Analytical Plan 40

2 – Output of Analysis 41
3 – Summary and Interpretation of Analysis Results 44

Research Question: Is there an association between Australian participants' region (metropolitan or regional) and their level of environmental concern? 45
1- Analytical Plan 45
2 – Output of Analysis 46
3 – Summary and Interpretation of Analysis Results 49

Research Question: Is there an association between UK participants' role (pharmacist or pharmacy technician) and their level of environmental concern? 50
1- Analytical Plan 50
2 – Output of Analysis 51
3 – Summary and Interpretation of Analysis Results 56

Research Question: Is there an association between UK participants' operational level and their level of environmental concern? 57
1- Analytical Plan 57
2- Output of Analysis 58
3 – Summary and Interpretation of Analysis Results 64

Research Question: Is there an association between UK participants' gender and their level of environmental concern? 65
1- Analytical Plan 65
2 – Output of Analysis 66
3 – Summary and Interpretation of Analysis Results 69

Research Question: Is there an association between UK participants' city and their level of environmental concern? 70
1- Analytical Plan 70
2 – Output of Analysis 71
3 – Summary and Interpretation of Analysis Results 74

Research Question: Is environmental attitude a predictor of level of environmental concern in Australian Participants? 75
1- Analytical Plan 75
2 - Output of Analysis 78
3 – Summary of Results 85

Research Question: Is environmental attitude a predictor of level of environmental concern in UK participants? 86
1- Analytical Plan 86
2 - Output of Analysis 89
3 – Summary of Results 94

Reliability of NEP Scale for Australian Sample

Each participant's response for each question was checked against their final score.

In SPSS, Analyse – Scale – Reliability Analysis. Under Item input each of the 15 questions. Model – select Alpha (Cronbach's Alpha), Scale label – select Nep_Tot.

Reliability

Scale: Nep_Tot

Case Processing Summary

		N	%
Cases	Valid	66	100.0
	Excluded[a]	0	.0
	Total	66	100.0

a. Listwise deletion based on all variables in the procedure.

Reliability Statistics

Cronbach's Alpha	N of Items
.815	15

Cronbach's alpha coefficient = 0.815 indicating that the NEP_Scale questionnaire has good internal consistency for this sample.

Reliability of NEP Scale for UK Sample

Each participant's response for each question was checked against their final score.

In SPSS, Analyse – Scale – Reliability Analysis. Under Item input each of the 15 questions. Model – select Alpha (Cronbach's Alpha), Scale label – select Nep_Tot.

Scale: NEP_Tot

Case Processing Summary

		N	%
Cases	Valid	40	100.0
	Excluded[a]	0	.0
	Total	40	100.0

a. Listwise deletion based on all variables in the procedure.

Reliability Statistics

Cronbach's Alpha	N of Items
.734	15

The Cronbach's Alpha is 0.734 indicating a reasonable internal consistency for the NEP scale in this sample. It is not as high as in the Australian sample.

Research Question: “Is there a difference in mean NEP score between Australian pharmacists and pharmacy technicians?”

1- Analytical Plan

Study Design: Observational – correlational – cross-section

Variables

DV: NEP score - continuous

IV: Role_Binary – categorical (dichotomous)

Hypotheses

H_0: There is no difference in mean NEP score between Australian pharmacists and pharmacy technicians

H_1: There is a difference in mean NEP score between Australian pharmacists and pharmacy technicians

Univariate Analysis

a) DV: NEP score

Numerical summary: mean and standard deviation if normally distributed and median and interquartile range if not (need to test for normality to decide which to use)

Graphical summary: Histogram

b) IV: Role_Binary

Numerical summary: Proportions (percentages)

Graphical summary: Bar chart

Bivariate Analysis

Numerical summary: Mean (sd) or median (IQR) by group depending on normality of distribution

Graphical summary: Side-by-side box and whisker plot

Statistical tests and assumptions

Test: Independent sample t-test

Assumptions for this test are:

1) distribution of responses within each group are normally distributed if sample size < 30 **OR** sample size > 30. If this assumption is not met then Mann-Whitney test will be used.

2) samples are independent – the study design suggests this assumption is met

3) Variances of BSA are the same males and females. This assumption will be checked using Levene's Test and appropriate corrections made if required.

Significance Levels

$p < 0.05$ will be used to indicate statistical significance

2 – Output of analysis

Univariate Analysis

a) DV: NEP score

Frequencies

Statistics

Nep_Tot

N	Valid	66
	Missing	0
Mean		54.58
Median		53.00
Std. Deviation		6.906
Variance		47.694
Skewness		.401
Std. Error of Skewness		.295
Kurtosis		.527
Std. Error of Kurtosis		.582
Range		34
Minimum		38
Maximum		72

Reliability Statistics

Cronbach's Alpha	Cronbach's Alpha Based on Standardized Items	N of Items
.810	.815	15

To check normality of data:

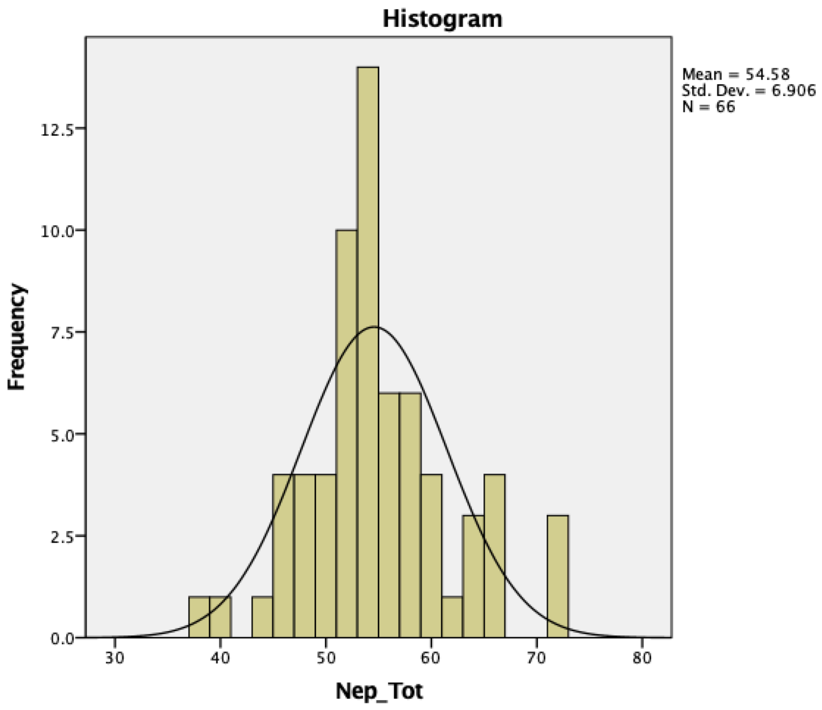

	NEP Score
n	66
Mean	54.58
Median	53
Median within 10% of mean?	Yes
sd	6.906
Mean +/- 3 sd	33.86-75.29
Minimum value	38
Maximum value	72
Skewness coefficient (Between -2 and 2)	0.582
Kurtosis coefficient (Between -2 and 2)	0.527
Are data normally distributed?	Yes

Data are normally distributed so report mean and standard deviation. The mean NEP score for the sample was 54.58 (sd=6.906).

b) IV: Role_Binary

Frequencies

Statistics

Role_Binary

N	Valid	66
	Missing	0

Role_Binary

		Frequency	Percent	Valid Percent	Cumulative Percent
Valid	Pharmacist	43	65.2	65.2	65.2
	Phamacy Technician	23	34.8	34.8	100.0
	Total	66	100.0	100.0	

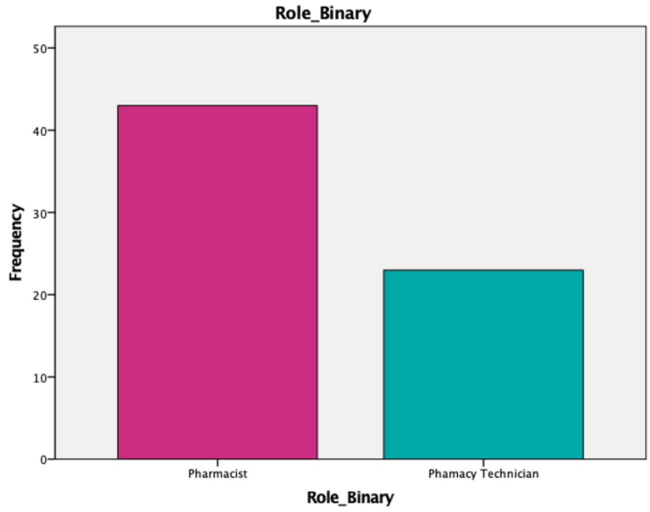

In the sample of 66 individuals, 65.2% (43/66) were pharmacists and 34.8% (23/66) were pharmacy technicians.

Bivariate Analysis

Need to explore the normality of NEP score in each group (pharmacists and pharmacy technicians to determine whether to report the mean (sd) or the median (IQR).

Explore

Role_Binary

Case Processing Summary

	Role_Binary	Cases Valid N	Valid Percent	Missing N	Missing Percent	Total N	Total Percent
Nep_Tot	Pharmacist	43	100.0%	0	0.0%	43	100.0%
	Phamacy Technician	23	100.0%	0	0.0%	23	100.0%

Descriptives

	Role_Binary			Statistic	Std. Error
Nep_Tot	Pharmacist	Mean		54.65	1.118
		95% Confidence Interval for Mean	Lower Bound	52.39	
			Upper Bound	56.91	
		5% Trimmed Mean		54.58	
		Median		54.00	
		Variance		53.756	
		Std. Deviation		7.332	
		Minimum		38	
		Maximum		72	
		Range		34	
		Interquartile Range		7	
		Skewness		.226	.361
		Kurtosis		.377	.709
	Phamacy Technician	Mean		54.43	1.290
		95% Confidence Interval for Mean	Lower Bound	51.76	
			Upper Bound	57.11	
		5% Trimmed Mean		54.11	
		Median		53.00	
		Variance		38.257	
		Std. Deviation		6.185	
		Minimum		44	
		Maximum		71	
		Range		27	
		Interquartile Range		7	
		Skewness		.966	.481
		Kurtosis		1.321	.935

	Pharmacists	Pharmacy Technicians
n	43	23
Mean	54.65	54.54
Median	54	53
Median within 10% of mean?	Yes	Yes
Mean +/- 3 sd	32.65-76.65	35.99-74.09
sd	7.332	6.185
Minimum value	38	44
Maximum value	72	71
Skewness coefficient (Between -2 and 2)	0.226 - yes	0.966 - yes
Kurtosis coefficient (Between -2 and 2)	0.377 - yes	1.321 - yes
Are data normally distributed?	Yes	Yes

Since data is normally distributed in each group mean and sd will be reported. The mean NEP score for pharmacists was 54.65 (sd=7.33; 95% CI: 52.39-56.91) and the mean NEP score for pharmacy technicians was 54.54 (sd=6.19; 95% CI: 51.76-57.11).

Statistical tests and assumptions

Test: Independent two-sample t-test

Assumptions for this test are:

1) distribution of responses within each group are normally distributed if sample size < 30 **OR** sample size > 30. This criterion was checked as one sample < 30 – data are normally distributed.

2) samples are independent – the study design suggests this assumption is met

3) Variances of NEP score are the same for pharmacists and pharmacy technicians. This assumption will be checked using Levene's Test and appropriate corrections made if required.

T-Test

Group Statistics

	Role_Binary	N	Mean	Std. Deviation	Std. Error Mean
NEP_Tot	Pharmacist	64	53.98	7.271	.909
	Pharmacy Technician	42	55.02	5.344	.825

Independent Samples Test

		Levene's Test for Equality of Variances		t-test for Equality of Means						
									95% Confidence Interval of the Difference	
		F	Sig.	t	df	Sig. (2-tailed)	Mean Difference	Std. Error Difference	Lower	Upper
NEP_Tot	Equal variances assumed	2.941	.089	-.796	104	.428	-1.039	1.307	-3.630	1.551
	Equal variances not assumed			-.847	102.592	.399	-1.039	1.227	-3.474	1.395

Levene's test: (F=2.941, p = 0.089) (> 0.05) and therefore accept H_0: variances of two groups are equal. Therefore, all assumptions of the Independent two-sample t-test are met. There is no statistically significant difference in mean NEP scores between pharmacists and pharmacy technicians in the sample (t_{104}= -0.796, p =0.428). On average, pharmacy technicians had a mean NEP score 1.04 higher than pharmacists (95% CI: -3.63-1.55).

3 – Summary and Interpretation of Analysis Results

In the sample of 66 individuals, 65.2% (43/66) were pharmacists and 34.8% (23/66) were pharmacy technicians. The mean NEP score for the sample was 54.58 (sd=6.906). An independent two-sample t-test was conducted. This test has three assumptions each of which was checked. The first assumption is that data are normally distributed – this was checked and affirmed. The second assumption is that the two samples are independent – the study design ensured this. For the third assumption (equal variances) Levene's test was conducted (F=2.941, p = 0.089) and equal variances was assumed. Therefore, all three assumptions of this test were met. There was no statistically significant difference in mean NEP scores between Australian pharmacists and pharmacy technicians in the sample (t_{104}= -0.796, p =0.428). On average, Australian pharmacy technicians had a mean NEP score 1.04 points higher than pharmacists (95% CI: -3.63-1.55).

Research Question: “Is there a difference in mean NEP score between UK pharmacists and pharmacy technicians?”

1- Analytical Plan

Study Design: Observational – correlational – cross-sectional

Variables:

DV: NEP score (continuous)

IV: Role (categorical – dichotomous) – Pharmacists or Pharmacy technicians

Hypotheses:

H_0: There is no difference in mean NEP scores for UK pharmacists and pharmacy technicians

H_1: There is a difference in mean NEP scores for UK pharmacists and pharmacy technicians

Univariate Analysis:

A) DV: NEP Score

Numerical summary: mean and standard deviation if normally distributed and median and interquartile range if not (need to test for normality to decide which to use)

Graphical summary: Histogram

b) IV: Role

Numerical summary: Frequencies (Count) and Proportion (Percentages)

Graphical summary: Bar graph

Bivariate Analysis:

Numerical summary: Mean (sd) or Median (IQR) by group depending on normality of distribution

Graphical summary: Side-by-side box and whisker plot

Statistical Tests and Assumptions

Test: two-sample t-test

Assumptions for this test are:

1) distribution of responses within each group are normally distributed if sample size < 30 OR sample size > 30, If this assumption is not met then a Mann-Whitney test will be used

Sample size > 30 so assumption is met.

2) Samples are independent – this study design suggests this assumption is met

3) Variances of NEP scores are the same for pharmacists and pharmacy technicians. This assumption will be checked using Levene’s test and appropriate corrections made if required.

Significance Levels

P < 0.05 will be used to indicate statistical significance.

2 – Output of Analysis

Univariate Analysis

a) DV: NEP Score

Numerical summary: mean and standard deviation if normally distributed and median and interquartile range if not (need to test for normality to decide which to use)

Graphical summary: Histogram

To test normality of distribution:

	NEP_Tot
n	40
Mean	54.10
Median	55.00
Median within 10% of mean?	yes
sd	6.042
Mean +/- 3 sd	35.974 - 72.226
Minimum value	40
Maximum value	66
Skewness coefficient between -2 and 2?	-0.327 -yes
Kurtosis coefficient between -2 and 2?	-0.267 -yes
Does histogram look approx. bell-shaped?	yes
Is data normally distributed?	yes

Statistics

NEP_Tot

N	Valid	40
	Missing	0
Mean		54.10
Median		55.00
Std. Deviation		6.042
Variance		36.503
Skewness		-.327
Std. Error of Skewness		.374
Kurtosis		-.267
Std. Error of Kurtosis		.733
Range		26
Minimum		40
Maximum		66

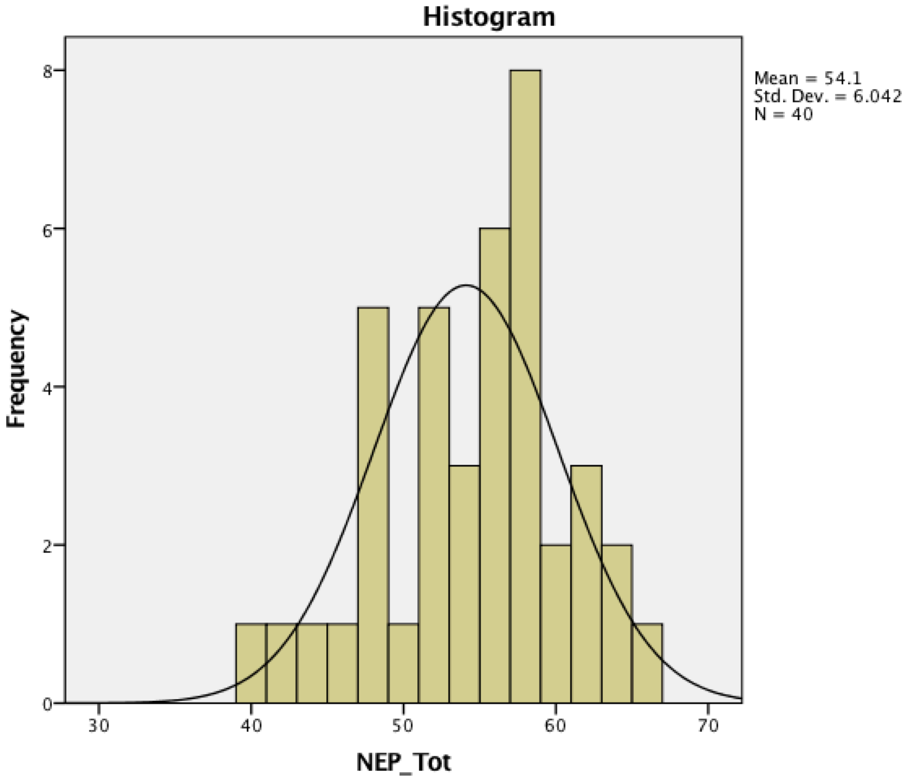

b) IV: Role

Numerical summary: Frequencies (Count) and Proportion (Percentages)

Graphical summary: Bar graph

Statistics

Role_Binary

N	Valid	40
	Missing	0

Role_Binary

		Frequency	Percent	Valid Percent	Cumulative Percent
Valid	Pharmacist	23	57.5	57.5	57.5
	Pharmacy Technician	17	42.5	42.5	100.0
	Total	40	100.0	100.0	

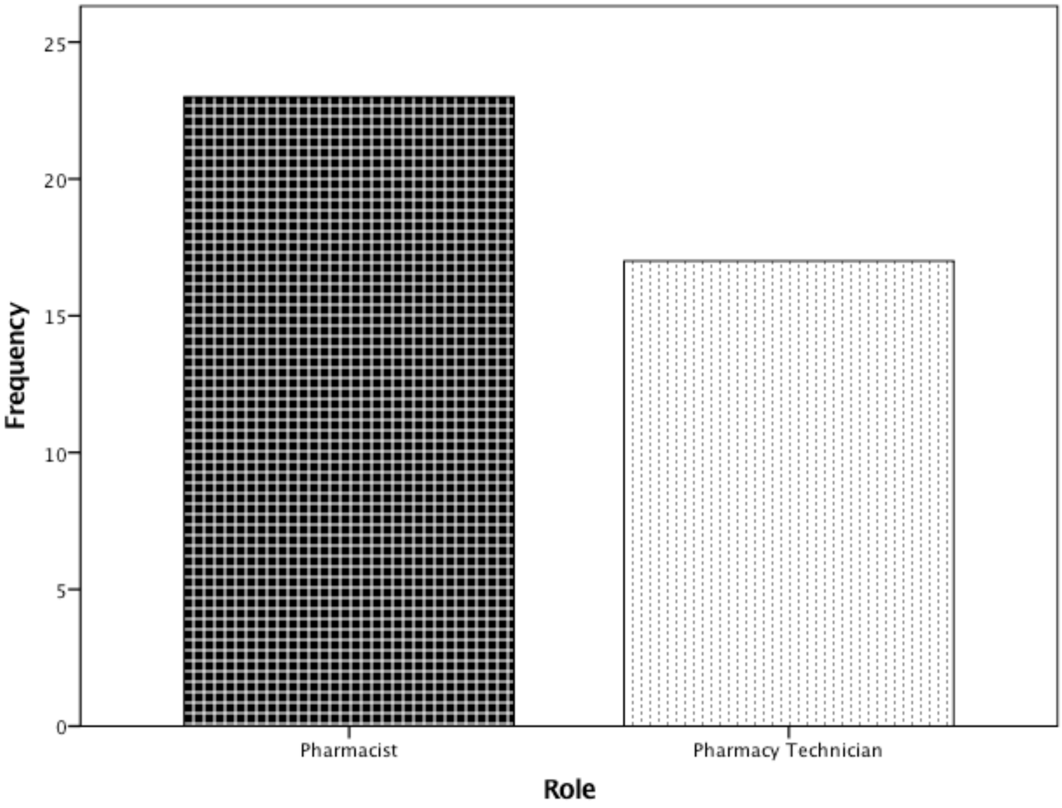

Bivariate Analysis

Case Processing Summary

	Role_Binary	Cases					
		Valid		Missing		Total	
		N	Percent	N	Percent	N	Percent
NEP_Tot	Pharmacist	23	100.0%	0	0.0%	23	100.0%
	Pharmacy Technician	17	100.0%	0	0.0%	17	100.0%

Descriptives

	Role_Binary			Statistic	Std. Error
NEP_Tot	Pharmacist	Mean		52.87	1.435
		95% Confidence Interval for Mean	Lower Bound	49.89	
			Upper Bound	55.85	
		5% Trimmed Mean		52.86	
		Median		53.00	
		Variance		47.391	
		Std. Deviation		6.884	
		Minimum		40	
		Maximum		66	
		Range		26	
		Interquartile Range		10	
		Skewness		.018	.481
		Kurtosis		-.560	.935
	Pharmacy Technician	Mean		55.76	1.049
		95% Confidence Interval for Mean	Lower Bound	53.54	
			Upper Bound	57.99	
		5% Trimmed Mean		55.79	
		Median		57.00	
		Variance		18.691	
		Std. Deviation		4.323	
		Minimum		48	
		Maximum		63	
		Range		15	
		Interquartile Range		6	
		Skewness		-.413	.550
		Kurtosis		-.314	1.063

Need to explore the normality of NEP score in each group (pharmacists and pharmacy technicians to determine whether to report the mean (sd) or the median (IQR).

	Pharmacists	Pharmacy Technicians
n	23	17
Mean	52.87	55.76
Median	53	57
Median within 10% of mean?	yes	yes
Mean +/- 3 sd	32.22-73.52	42.79-68.73
sd	6.844	4.323
Minimum value	40	48
Maximum value	66	63
Skewness coefficient (Between -2 and 2)	0.018 - yes	-0.413 - yes
Kurtosis coefficient (Between -2 and 2)	-0.560 - yes	-0.314 - yes
Are data normally distributed?	Yes	Yes

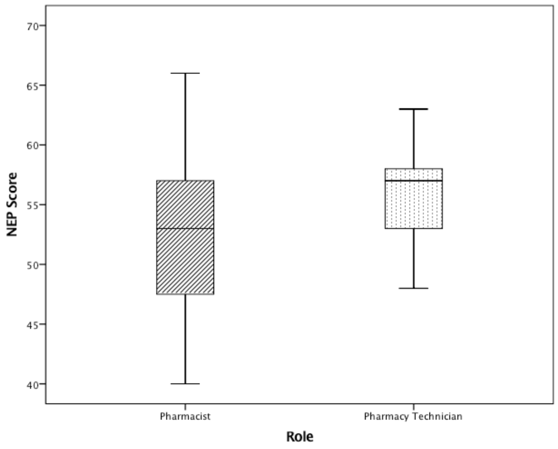

Statistical Tests and Assumptions

Test: Independent two-sample t-test

All assumptions for Independent 2-sample t-test were met.

T-Test

Group Statistics

	Role_Binary	N	Mean	Std. Deviation	Std. Error Mean
NEP_Tot	Pharmacist	23	52.87	6.884	1.435
	Pharmacy Technician	17	55.76	4.323	1.049

Independent Samples Test

		Levene's Test for Equality of Variances		t-test for Equality of Means						
									95% Confidence Interval of the Difference	
		F	Sig.	t	df	Sig. (2-tailed)	Mean Difference	Std. Error Difference	Lower	Upper
NEP_Tot	Equal variances assumed	3.895	.056	-1.523	38	.136	-2.895	1.901	-6.743	.952
	Equal variances not assumed			-1.629	37.185	.112	-2.895	1.778	-6.496	.706

3 – Summary and Interpretation of Analysis Results

In the sample of 40 participants, there were 23 pharmacists with a mean NEP score of 52.87 (sd = 6.884; 95% CI: 49.89 – 55.85) and 17 pharmacy technicians with a mean NEP score of 55.76 (sd = 4.323; 95% CI: 53.54 – 57.99).

Equal variances in NEP scores were checked using Levene's test and equal variances were assumed for pharmacists and pharmacy technicians (F = 3.895, p = 0.056). There was no statistically significant difference in mean NEP scores between pharmacists and pharmacy technicians in this sample (t_{38} = -1.523, p = 0.136). On average, UK pharmacy technicians had a mean NEP score 2.895 points higher than pharmacists (95% CI: -6.74-0.95).

Research Question: Is there a difference in mean NEP scores between Australian and UK participants?

1- Analytical Plan

Study Design: Observational – correlational – cross-sectional
Variables:
DV: NEP score (continuous)
IV: Country (Australia or UK)

Hypotheses:
H_0: There is no difference in mean NEP scores for Australian and UK participants
H_1: There is a difference in mean NEP scores for Australian and UK participants

Univariate Analysis:

a) DV: NEP Score

Numerical summary: mean and standard deviation if normally distributed and median and interquartile range if not (need to test for normality to decide which to use)

b) IV: Country

Numerical summary: Frequencies (Count) and Proportion (Percentages)
Graphical summary: Bar graph

Bivariate Analysis:
Numerical summary: Mean (sd) or Median (IQR) by group depending on normality of distribution
Graphical summary: Side-by-side box and whisker plot

Statistical Tests and Assumptions
Test: Independent two-sample t-test
Assumptions for this test are:
1) distribution of responses within each group are normally distributed if sample size < 30 OR sample size > 30, If this assumption is not met then a Mann-Whitney test will be used

Sample size > 30 so assumption is met.

2) Samples are independent – this study design suggests this assumption is met

3) Variances of NEP scores are the same for pharmacists and pharmacy technicians. This assumption will be checked using Levene's test and appropriate corrections made if required.

Significance Levels

P < 0.05 will be used to indicate statistical significance.

2 – Output of Analysis

Reliability

Scale: NEP_Tot

Case Processing Summary

		N	%
Cases	Valid	106	100.0
	Excluded[a]	0	.0
	Total	106	100.0

a. Listwise deletion based on all variables in the procedure.

Reliability Statistics

Cronbach's Alpha	N of Items
.787	15

Univariate Analysis

a) DV: NEP Score

Numerical summary: mean and standard deviation if normally distributed and median and interquartile range if not (need to test for normality to decide which to use)

Frequencies

Statistics

NEP_Tot

N	Valid	106
	Missing	0
Mean		54.40
Median		54.00
Std. Deviation		6.568
Variance		43.137
Skewness		.207
Std. Error of Skewness		.235
Kurtosis		.370
Std. Error of Kurtosis		.465
Range		34
Minimum		38
Maximum		72

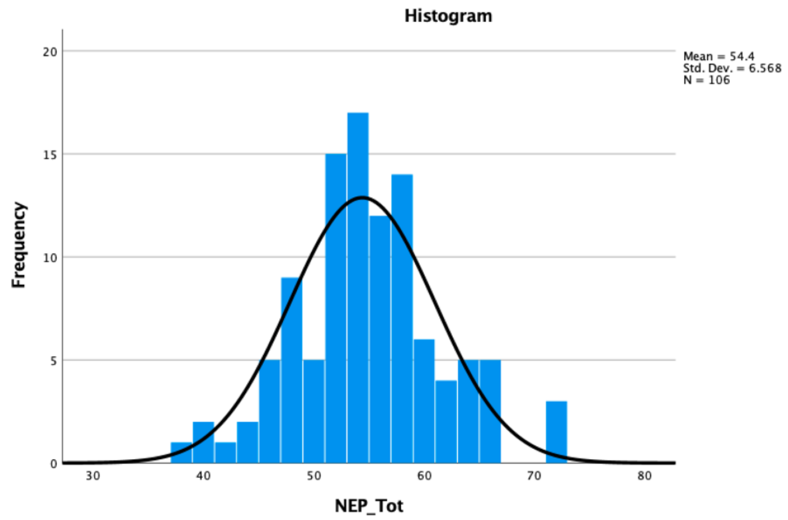

To test normality of data distribution:

	NEP Score
n	106
Mean	54.5
Median	54
Median within 10% of mean?	yes
sd	6.568
Mean +/- 3 sd	34.80-74.20
Minimum value	38
Maximum value	72
Skewness coefficient (Between -2 and 2)	0.207 - yes
Kurtosis coefficient (Between -2 and 2)	0.370 - yes
Are data normally distributed?	Yes

The mean NEP score for the sample of 106 participants was 54.5 (sd=6.57).

b) IV: Country

Numerical summary: Frequencies (Count) and Proportion (Percentages)

Graphical summary: Bar graph

Group Statistics

	Country	N	Mean	Std. Deviation	Std. Error Mean
NEP_Tot	Australia	66	54.58	6.906	.850
	England	40	54.10	6.042	.955

		Frequency	Percent	Valid Percent	Cumulative Percent
Valid	Australia	66	62.3	62.3	62.3
	England	40	37.7	37.7	100.0
	Total	106	100.0	100.0	

Country

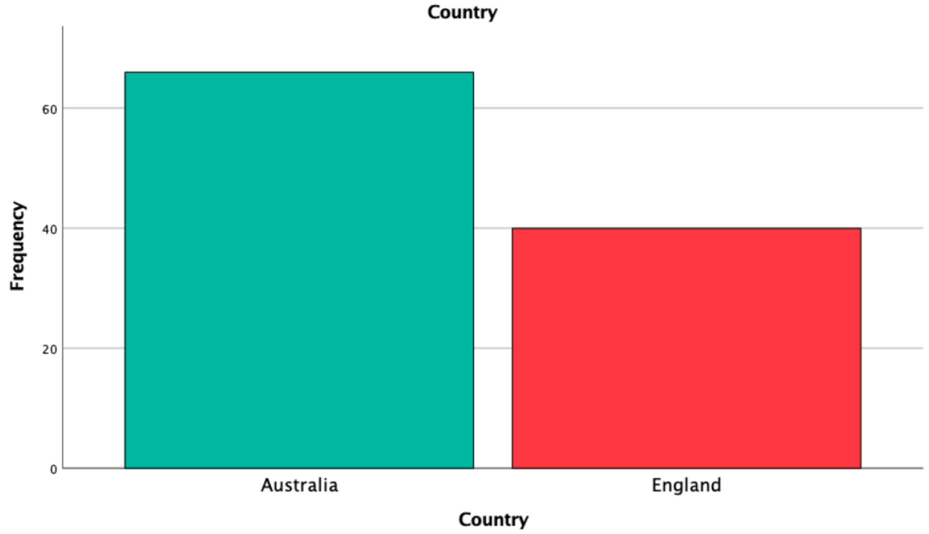

Bivariate Analysis

Numerical summary: Mean (sd) or Median (IQR) by group depending on normality of distribution
Graphical summary: Side-by-side box and whisker plot

To test normality of distribution:

	Australia	UK
n	66	40
Mean	54.58	54.10
Median	53	55.00
Median within 10% of mean?	Yes	yes
Mean +/- 3 sd	6.906	6.042
sd	33.86-75.29	35.974 - 72.226
Minimum value	38	40
Maximum value	72	66
Skewness coefficient (Between -2 and 2)	0.582	-0.327 -yes
Kurtosis coefficient (Between -2 and 2)	0.527	-0.267 -yes
Are data normally distributed?	Yes	yes

The mean NEP score for Australian participants was 54.58 (sd=6.91; 95%CI: 52.88-56.27).
The mean NEP score for the UK participants was 54.10 (sd=6.042; 95% CI: 52.17-56.03).

Explore

Country

Case Processing Summary

	Country	Cases					
		Valid		Missing		Total	
		N	Percent	N	Percent	N	Percent
NEP_Tot	Australia	66	100.0%	0	0.0%	66	100.0%
	England	40	100.0%	0	0.0%	40	100.0%

Descriptives

	Country			Statistic	Std. Error
NEP_Tot	Australia	Mean		54.58	.850
		95% Confidence Interval for Mean	Lower Bound	52.88	
			Upper Bound	56.27	
		5% Trimmed Mean		54.42	
		Median		53.00	
		Variance		47.694	
		Std. Deviation		6.906	
		Minimum		38	
		Maximum		72	
		Range		34	
		Interquartile Range		7	
		Skewness		.401	.295
		Kurtosis		.527	.582
	England	Mean		54.10	.955
		95% Confidence Interval for Mean	Lower Bound	52.17	
			Upper Bound	56.03	
		5% Trimmed Mean		54.22	
		Median		55.00	
		Variance		36.503	
		Std. Deviation		6.042	
		Minimum		40	
		Maximum		66	
		Range		26	
		Interquartile Range		9	
		Skewness		-.327	.374
		Kurtosis		-.267	.733

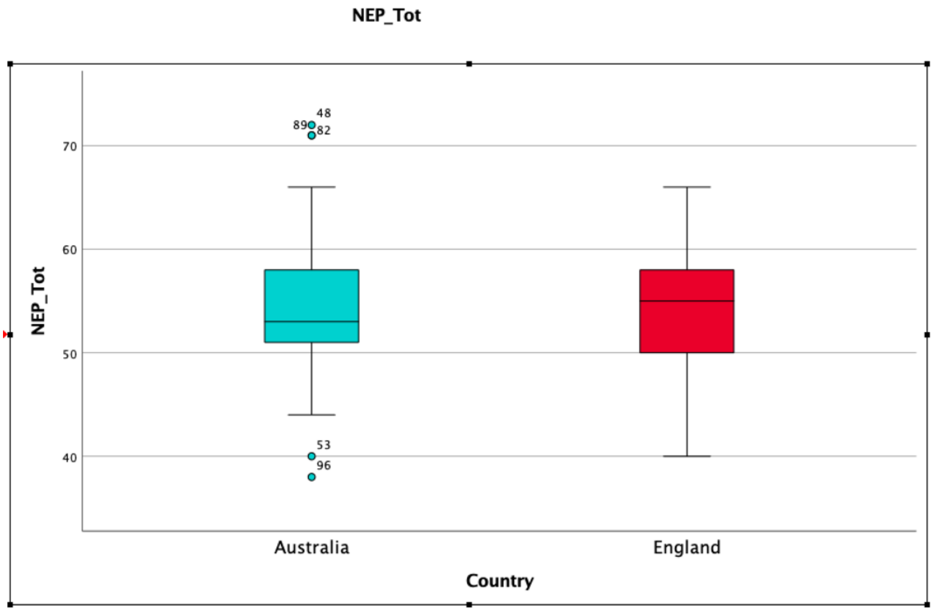

Statistical Tests and Assumptions

Test: Independent two-sample t-test

All assumptions for Independent 2-sample t-test were met.

T-Test

Group Statistics

	Country	N	Mean	Std. Deviation	Std. Error Mean
NEP_Tot	Australia	66	54.58	6.906	.850
	England	40	54.10	6.042	.955

Independent Samples Test

		Levene's Test for Equality of Variances		t-test for Equality of Means					95% Confidence Interval of the Difference	
		F	Sig.	t	df	Sig. (2-tailed)	Mean Difference	Std. Error Difference	Lower	Upper
NEP_Tot	Equal variances assumed	.135	.714	.360	104	.720	.476	1.322	-2.145	3.096
	Equal variances not assumed			.372	90.988	.711	.476	1.279	-2.064	3.016

3 – Summary and Interpretation of Analysis Results

In the sample of 106 participants, there were 66 Australians with a mean NEP score of 54.58 (sd=6.91; 95%CI: 52.88-56.27) and 40 UK participants with a mean NEP score of 54.10 (sd=6.042; 95% CI: 52.17-56.03).

Equal variances in NEP scores were checked using Levene's test and equal variances were assumed for pharmacists and pharmacy technicians (F = 0.135, p = 0.714). There was no statistically significant difference in mean NEP scores between Australian and UK participants in this sample (t_{104} = 0.36, p = 0.72). Australian participants' mean NEP score was 0.476 higher than UK participants.

Research Question: Is there a difference in level of environmental concern between Australian and UK participants?

1- Analytical Plan

Study Design: Observational – correlational – cross-sectional

Variables:

DV: Environmental concern – dichotomous categorical (concerned/not concerned)

IV: Country (Australia or UK) – dichotomous categorical

Hypotheses:

H_0: The proportion of participants showing environmental concern is similar for the Australian and UK cohorts

H_1: The proportion of participants showing environmental concern is different for the Australian and UK cohorts

Univariate Analysis:

a) DV: Environmental Concern

Numerical summary: Frequencies (Count) and Proportion (Percentages)

Graphical summary: Bar graph

b) IV: Country

Numerical summary: Frequencies (Count) and Proportion (Percentages)

Graphical summary: Bar graph

Bivariate Analysis:

Numerical summary: 2 x 2 contingency table

Graphical summary: Side-by-side bar chart

Statistical Tests and Assumptions

Test: Fisher's Exact Test

Assumptions for this test are:

1) observations are independent – research design covers this

Significance Levels

$P < 0.05$ will be used to indicate statistical significance.

2 – Output of Analysis

Univariate Analysis

a) DV: Environmental Concern

Numerical summary: Frequencies (Count) and Proportion (Percentages)

Graphical summary: Bar graph

Statistics

Env_Concern

N	Valid	104
	Missing	2

Env_Concern

		Frequency	Percent	Valid Percent	Cumulative Percent
Valid	Not concerned	52	49.1	50.0	50.0
	Concerned	52	49.1	50.0	100.0
	Total	104	98.1	100.0	
Missing	999	2	1.9		
Total		106	100.0		

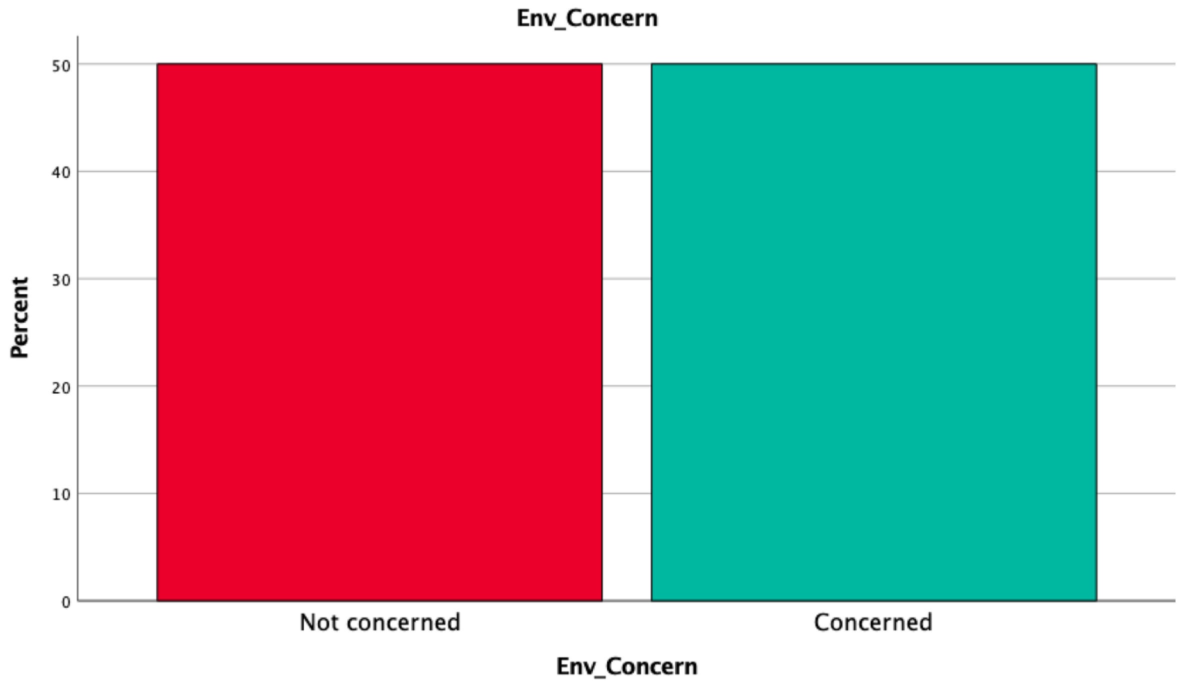

b) IV: Country

Numerical summary: Frequencies (Count) and Proportion (Percentages)

Graphical summary: Bar graph

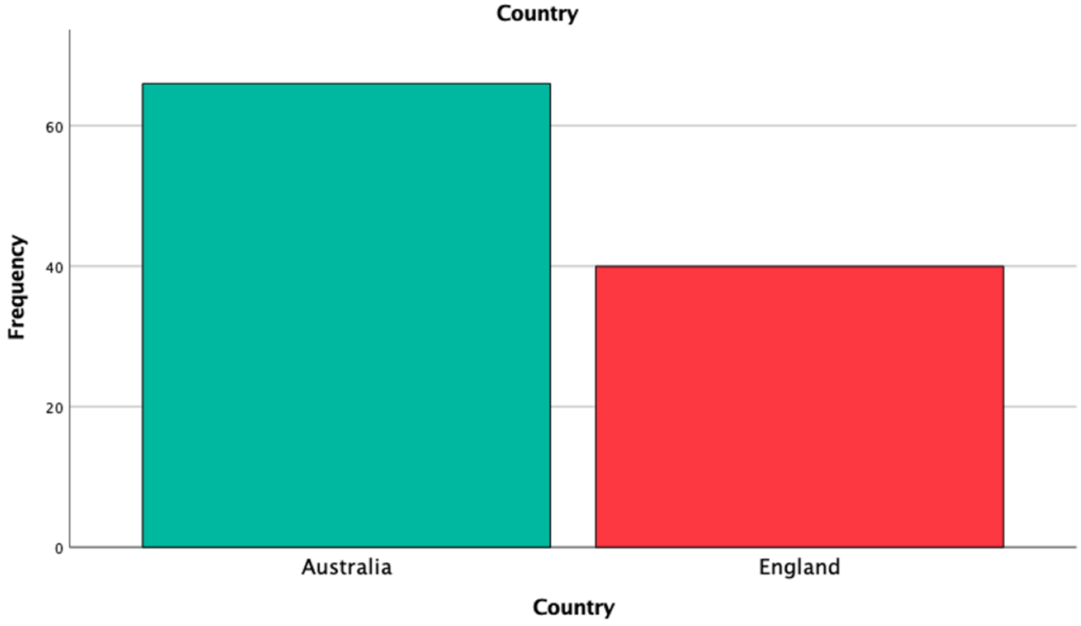

Bivariate Analysis

Numerical summary: 2 x 2 contingency table
Graphical summary: Side-by-side bar chart

Crosstabs

Case Processing Summary

	Cases					
	Valid		Missing		Total	
	N	Percent	N	Percent	N	Percent
Env_Concern * Country	104	98.1%	2	1.9%	106	100.0%

Env_Concern * Country Crosstabulation

Count

		Country		Total
		Australia	England	
Env_Concern	Not concerned	28	24	52
	Concerned	36	16	52
Total		64	40	104

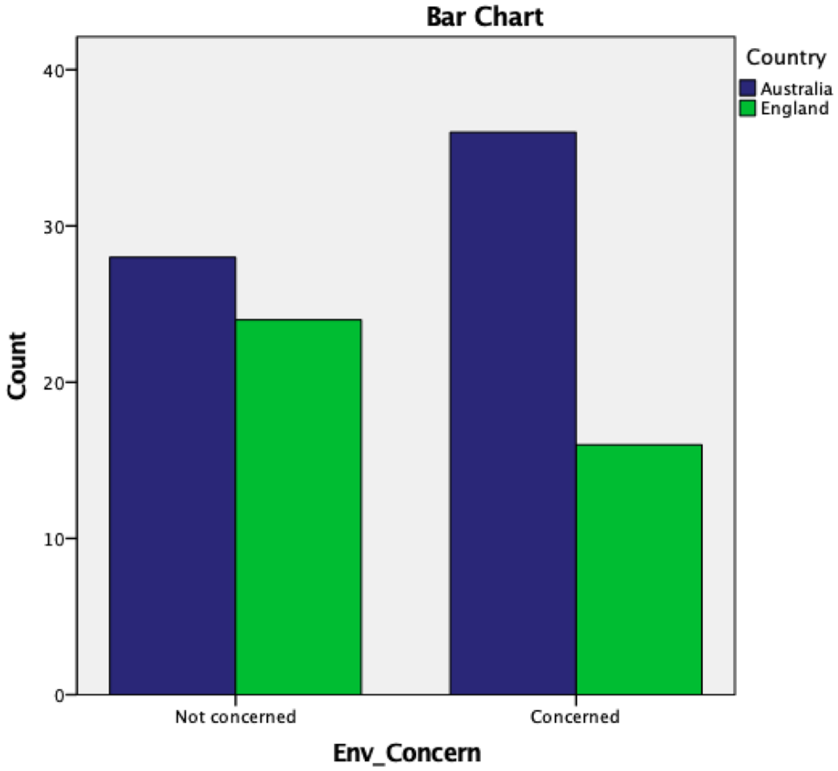

Statistical Tests and Assumptions

Test: Fisher's Exact Test

Assumption: observations are independent are satisfied by research design.

Env_Concern * Country Crosstabulation

Count

		Country		Total
		Australia	England	
Env_Concern	Not concerned	28	24	52
	Concerned	36	16	52
Total		64	40	104

Chi-Square Tests

	Value	df	Asymptotic Significance (2-sided)	Exact Sig. (2-sided)	Exact Sig. (1-sided)
Pearson Chi-Square	2.600[a]	1	.107		
Continuity Correction[b]	1.991	1	.158		
Likelihood Ratio	2.613	1	.106		
Fisher's Exact Test				.158	.079
Linear-by-Linear Association	2.575	1	.109		
N of Valid Cases	104				

a. 0 cells (.0%) have expected count less than 5. The minimum expected count is 20.00.

b. Computed only for a 2x2 table

3 – Summary and Interpretation of Analysis Results

Of the 106 participants, data were missing for two Australian participants. The sample comprised 61.54% (64/106) Australian participants and 38.46% (40/104) UK participants. Of the 104 participants for whom there were data, 49.1% (52/104) expressed concern for pharmaceuticals entering the natural environment. A higher percentage of Australian participants (56.25%; 36/64) compared with UK participants (40%; 16/40) reported feeling concerned about the impact of pharmaceuticals entering the environment (Table 5). Fisher's Exact Test was used to determine if there was a statistically significant difference in environmental concern between Australian and UK participants and no difference was found (Fisher's Exact Test, $p=0.158$).

Research Question: Is there an association between Australian participants' role (pharmacist or pharmacy technician) and their level of environmental concern?

1- Analytical Plan

Study Design: Observational – correlational – cross-sectional

Variables:

DV: Level of environmental concern – categorical - dichotomous

IV: Role – categorical - dichotomous

Hypotheses:

H_0: There is no association between a participant's role and their level of environmental concern

H_1: There is an association between a participant's role and their level of environmental concern

Univariate Analysis:

a) DV: Level of environmental concern

Numerical summary: Frequencies (Count) and Proportion (Percentages)

Graphical summary: Bar chart

b) IV: Role

Numerical summary: Frequencies (Count) and Proportion (Percentages)

Graphical summary: Bar graph

Bivariate Analysis:

Numerical summary: Mean (sd) or Median (IQR) by group depending on normality of distribution

Graphical summary: Side-by-side box and whisker plot

Statistical Tests and Assumptions

Test: Fisher's Exact Test

Assumptions for this test are:

- Observational units are independent – assume this is so because study is cross-sectional

Significance Levels

$p < 0.05$ will be used to indicate statistical significance.

2 – Output of Analysis

a) DV: Level of environmental concern

See page 34

b) IV: Role

Numerical summary: Frequencies (Count) and Proportion (Percentages)

Graphical summary: Bar graph

Statistics

Role_Binary

N	Valid	66
	Missing	0

Role_Binary

		Frequency	Percent	Valid Percent	Cumulative Percent
Valid	Pharmacist	43	65.2	65.2	65.2
	Phamacy Technician	23	34.8	34.8	100.0
	Total	66	100.0	100.0	

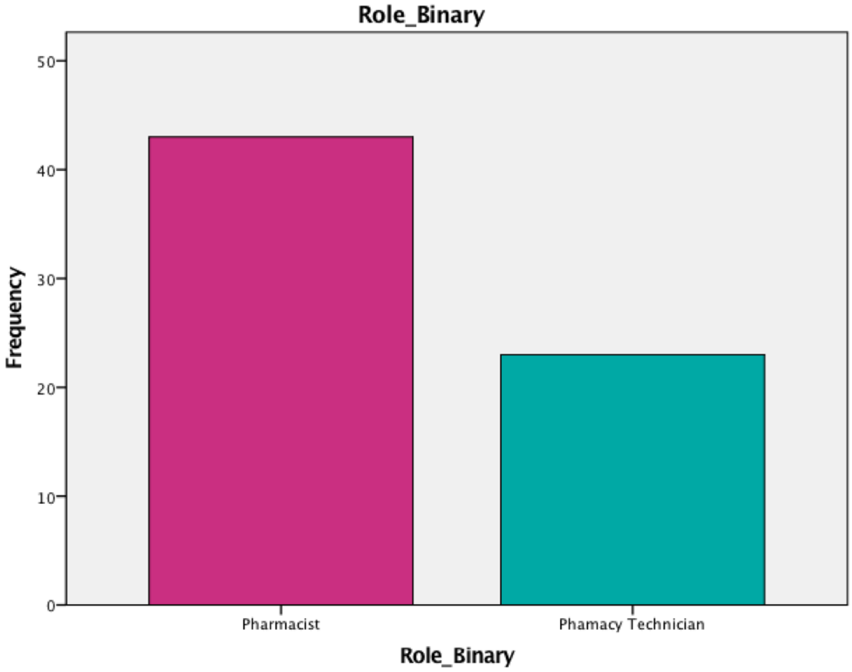

Bivariate Analysis:

Numerical summary: Mean (sd) or Median (IQR) by group depending on normality of distribution

Graphical summary: Side-by-side box and whisker plot

Case Processing Summary

	Cases					
	Valid		Missing		Total	
	N	Percent	N	Percent	N	Percent
Role_Binary * EnvironmentalConcern	66	100.0%	0	0.0%	66	100.0%

Role_Binary * EnvironmentalConcern Crosstabulation

Count

		EnvironmentalConcern		Total
		Concerned	Not concerned	
Role_Binary	Pharmacist	24	19	43
	Phamacy Technician	14	9	23
Total		38	28	66

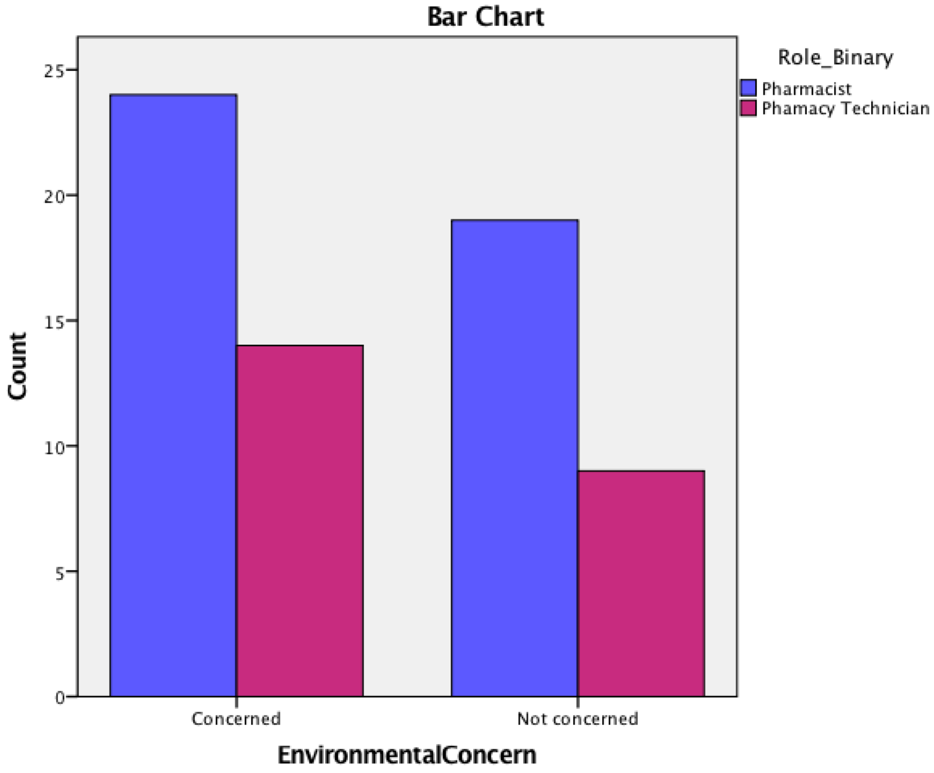

Statistical Testing

Chi-Square Tests

	Value	df	Asymptotic Significance (2-sided)	Exact Sig. (2-sided)	Exact Sig. (1-sided)
Pearson Chi-Square	.157[a]	1	.692		
Continuity Correction[b]	.018	1	.893		
Likelihood Ratio	.157	1	.692		
Fisher's Exact Test				.796	.448
Linear-by-Linear Association	.154	1	.694		
N of Valid Cases	66				

a. 0 cells (0.0%) have expected count less than 5. The minimum expected count is 9.76.

b. Computed only for a 2x2 table

3 – Summary and Interpretation of Analysis Results

Of the 66 participants, 43 were pharmacists and 23 were pharmacy technicians. Of the pharmacists, 24 (56%) were concerned, and of the pharmacy technicians 14 (60.87%) were concerned. There was no statistically significant difference in level of environmental concern between pharmacists and pharmacy technicians (Fisher's Exact test, $p = 0.796$).

Research Question: Is there an association between Australian participants' operational level and their level of environmental concern?

1- Analytical Plan

Study Design: Observational – correlational – cross-sectional

Variables:

DV: Level of environmental concern – categorical - dichotomous

IV: Operational level – categorical - ordinal

Hypotheses:

H_0: There is no association between a participant's operational level and their level of environmental concern

H_1: There is an association between a participant's operational level and their level of environmental concern

Univariate Analysis:

a) DV: Level of environmental concern

Numerical summary: Frequencies (Count) and Proportion (Percentages)

Graphical summary: Bar chart

b) IV: Operational Level

Numerical summary: Frequencies (Count) and Proportion (Percentages)

Graphical summary: Bar graph

Bivariate Analysis:

Numerical summary: Mean (sd) or Median (IQR) by group depending on normality of distribution

Graphical summary: Side-by-side box and whisker plot

Statistical Tests and Assumptions

Test: Pearson's chi-square test

Assumptions for this test are:

- Observational units are independent – assume this is so because study is cross-sectional
- Expected cell counts are adequate – all expected cell counts should be ≥ 1 and no more than 20% of cell counts should be ≤ 5

Significance Levels

$p < 0.05$ will be used to indicate statistical significance.

2- Output of Analysis

Univariate Analysis:

a) DV: Level of environmental concern

See page 34

b) IV: Operational Level

Numerical summary: Frequencies (Count) and Proportion (Percentages)

Graphical summary: Bar graph

Statistics

Level_2

		Statistic	Bootstrap[a] Bias	Std. Error	95% Confidence Interval Lower	Upper
N	Valid	66	0	0	66	66
	Missing	0	0	0	0	0

a. Unless otherwise noted, bootstrap results are based on 1000 bootstrap samples

Level_2

		Frequency	Percent	Valid Percent	Cumulative Percent	Bootstrap for Percent[a] Bias	Std. Error	95% Confidence Interval Lower	Upper
Valid	HP6 or above	6	9.1	9.1	9.1	.1	3.5	3.0	16.7
	HP5	11	16.7	16.7	25.8	.0	4.7	7.6	25.8
	HP4	11	16.7	16.7	42.4	-.2	4.6	7.6	27.3
	HP3 and HP2	15	22.7	22.7	65.2	-.1	5.1	12.1	33.3
	OO3 and above	23	34.8	34.8	100.0	.2	5.7	24.2	47.0
	Total	66	100.0	100.0		.0	.0	100.0	100.0

a. Unless otherwise noted, bootstrap results are based on 1000 bootstrap samples

To ensure all categories had adequate cell counts, the two categories HP3 and HP2 were merged as well as the OO4 and OO3 categories. Six (9.1%) participants were in the HP6 or above category, 11 (16.7%) were in the HP5 category, 11 (16.7%) were in the HP4 category, 15 (22.7%) were in the HP3/HP2 category and 23 (34.8%) were in the OO3 and above category.

Bivariate Analysis:

Numerical summary: Mean (sd) or Median (IQR) by group depending on normality of distribution

Graphical summary: Side-by-side box and whisker plot

Level_2 * EnvironmentalConcern Crosstabulation

Count

		EnvironmentalConcern		Total
		Concerned	Not concerned	
Level_2	HP6 or above	3	3	6
	HP5	7	4	11
	HP4	7	4	11
	HP3 and HP2	7	8	15
	OO3 and above	14	9	23
Total		38	28	66

Statistical Tests and Assumptions

Test: Pearson's chi-square test

All assumptions for this test were met.

Chi-Square Tests

	Value	df	Asymptotic Significance (2-sided)
Pearson Chi-Square	1.305[a]	4	.861
Likelihood Ratio	1.299	4	.862
Linear-by-Linear Association	.000	1	.982
N of Valid Cases	66		

a. 4 cells (40.0%) have expected count less than 5. The minimum expected count is 2.55.

3 – Summary and Interpretation of Analysis Results

To ensure all categories had adequate cell counts for statistical testing, the two categories HP3 and HP2 were merged as well as the OO4 and OO3 categories. Six (9.1%) participants were in the HP6 or above category, 11 (16.7%) were in the HP5 category, 11 (16.7%) were in the HP4 category, 15 (22.7%) were in the HP3/HP2 category and 23 (34.8%) were in the OO3 and above category. Of the six HP6 and above participants, there were equal numbers of concerned and not concerned. In the HP5 and HP4 categories, 64.64% (7/11) were concerned, in the HP3/HP2 category 46.67% (7/15) were concerned and in the OO3/OO4 category 60.87% (14/23) were concerned. There was no statistically significant difference in level of environmental concern between the five categories (λ_4^2 = 1.305, p = 0.861).

Research Question: Is there an association between Australian participants' gender and their level of environmental concern?

1- Analytical Plan

Study Design: Observational – correlational – cross-sectional

Variables:

DV: Level of environmental concern – categorical - dichotomous

IV: Gender – categorical - dichotomous

Hypotheses:

H_0: There is no association between a participant's gender and their level of environmental concern

H_1: There is an association between a participant's gender and their level of environmental concern

Univariate Analysis:

a) DV: Level of environmental concern

Numerical summary: Frequencies (Count) and Proportion (Percentages)

Graphical summary: Bar chart

b) IV: Gender

Numerical summary: Frequencies (Count) and Proportion (Percentages)

Graphical summary: Bar graph

Bivariate Analysis:

Numerical summary: Mean (sd) or Median (IQR) by group depending on normality of distribution

Graphical summary: Side-by-side box and whisker plot

Statistical Tests and Assumptions

Test: Fisher's Exact test

Assumptions for this test are:

- Observational units are independent – assume this is so because study is cross-sectional

Significance Levels

$p < 0.05$ will be used to indicate statistical significance.

2 – Output of Analysis

Univariate Analysis:

a) DV: Level of environmental concern

See page 34

b) IV: Gender

Numerical summary: Frequencies (Count) and Proportion (Percentages)

Graphical summary: Bar graph

Gender

						Bootstrap for Percent[a]			
								95% Confidence Interval	
		Frequency	Percent	Valid Percent	Cumulative Percent	Bias	Std. Error	Lower	Upper
Valid	Male	18	27.3	27.3	27.3	-.1	5.7	16.7	37.9
	Female	48	72.7	72.7	100.0	.1	5.7	62.1	83.3
	Total	66	100.0	100.0		.0	.0	100.0	100.0

a. Unless otherwise noted, bootstrap results are based on 1000 bootstrap samples

Bivariate Analysis:

Numerical summary: Mean (sd) or Median (IQR) by group depending on normality of distribution

Graphical summary: Side-by-side box and whisker plot

Gender * EnvironmentalConcern Crosstabulation

Count

		EnvironmentalConcern		Total
		Concerned	Not concerned	
Gender	Male	8	10	18
	Female	30	18	48
Total		38	28	66

Of the 66 participants, 38 (57.58%) expressed concern for environment. Of the 38 concerned participants, eight (44.44%) were male and 30 (62.5%) were female.

EnvironmentalConcern * Gender Crosstabulation

Count

		Gender		Total
		Male	Female	
EnvironmentalConcern	Concerned	8	30	38
	Not concerned	10	18	28
Total		18	48	66

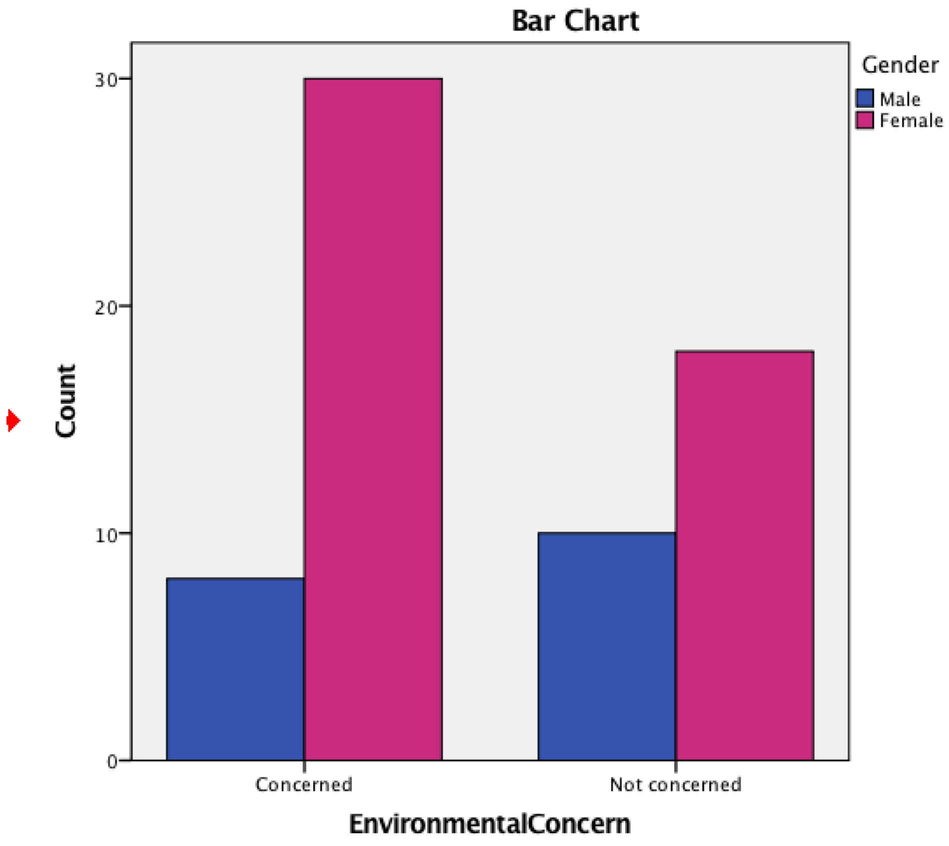

Statistical Tests and Assumptions

Chi-Square Tests

	Value	df	Asymptotic Significance (2-sided)	Exact Sig. (2-sided)	Exact Sig. (1-sided)
Pearson Chi-Square	1.747[a]	1	.186		
Continuity Correction[b]	1.086	1	.297		
Likelihood Ratio	1.734	1	.188		
Fisher's Exact Test				.264	.149
Linear-by-Linear Association	1.721	1	.190		
N of Valid Cases	66				

a. 0 cells (0.0%) have expected count less than 5. The minimum expected count is 7.64.

b. Computed only for a 2x2 table

3 – Summary and Interpretation of Analysis Results

Of the 66 participants, 18 (27.27%) were male and 38 (72.73%) were female, with 38 (57.58%) participants expressing concern for environment. Of the 38 concerned participants, eight (44.44%) were male and 30 (62.5%) were female. There was no statistically significant difference between male and female participants with regards to their levels of environmental concern (Fisher's exact test, p = 0.264).

Research Question: Is there an association between Australian participants' region (metropolitan or regional) and their level of environmental concern?

1- Analytical Plan

Study Design: Observational – correlational – cross-sectional

Variables:

DV: Level of environmental concern – categorical - dichotomous

IV: Region – categorical

Hypotheses:

H_0: There is no association between a participant's region and their level of environmental concern

H_1: There is an association between a participant's gender and their level of environmental concern

Univariate Analysis:

a) DV: Level of environmental concern

Numerical summary: Frequencies (Count) and Proportion (Percentages)

Graphical summary: Bar chart

b) IV: Region

Numerical summary: Frequencies (Count) and Proportion (Percentages)

Graphical summary: Bar graph

Bivariate Analysis:

Numerical summary: Mean (sd) or Median (IQR) by group depending on normality of distribution

Graphical summary: Side-by-side box and whisker plot

Statistical Tests and Assumptions

Test: Fisher's Exact test

Assumptions for this test are:

- Observational units are independent – assume this is so because study is cross-sectional

Significance Levels

$p < 0.05$ will be used to indicate statistical significance.

2 – Output of Analysis

Univariate Analysis:

a) DV: Level of environmental concern

See page 34

b) IV: Region

Numerical summary: Frequencies (Count) and Proportion (Percentages)

Graphical summary: Bar graph

Frequencies

Statistics

Region

N	Valid	66
	Missing	0

Region

		Frequency	Percent	Valid Percent	Cumulative Percent
Valid	Regional	24	36.4	36.4	36.4
	Metropolitan	42	63.6	63.6	100.0
	Total	66	100.0	100.0	

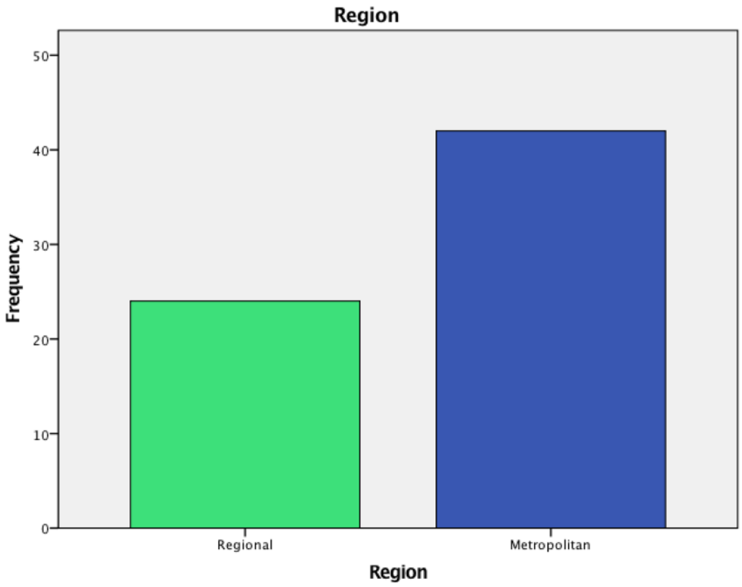

Bivariate Analysis:

Case Processing Summary

	Cases					
	Valid		Missing		Total	
	N	Percent	N	Percent	N	Percent
EnvironmentalConcern * Region	66	100.0%	0	0.0%	66	100.0%

EnvironmentalConcern * Region Crosstabulation

Count

		Region		Total
		Regional	Metropolitan	
EnvironmentalConcern	Concerned	15	23	38
	Not concerned	9	19	28
Total		24	42	66

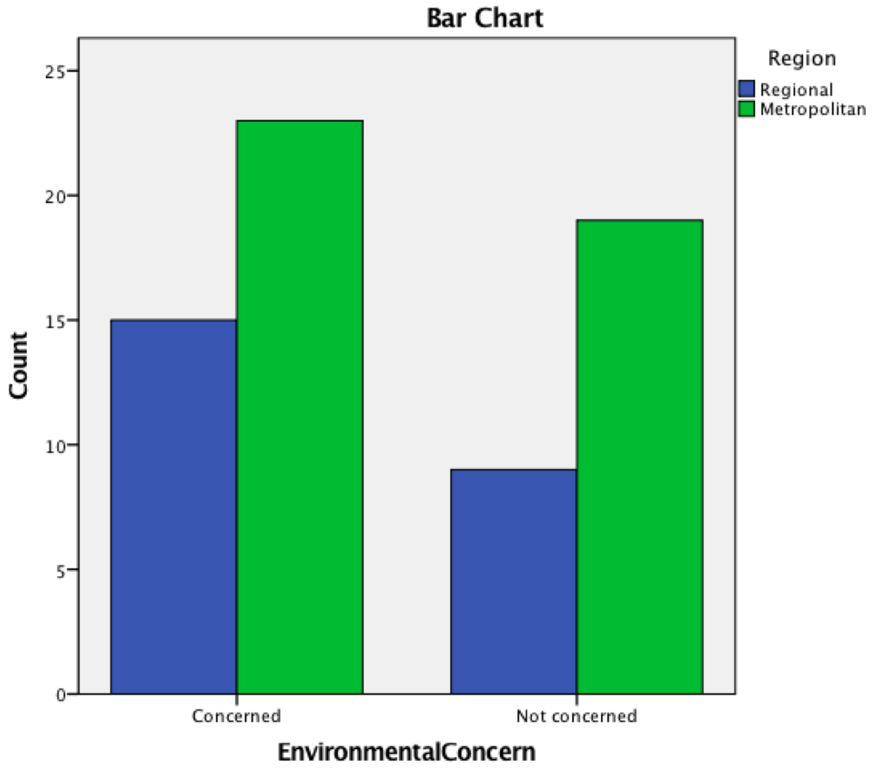

Statistical Tests and Assumptions

Test: Fisher's Exact test – all assumptions are met

Chi-Square Tests

	Value	df	Asymptotic Significance (2-sided)	Exact Sig. (2-sided)	Exact Sig. (1-sided)
Pearson Chi-Square	.374[a]	1	.541		
Continuity Correction[b]	.125	1	.724		
Likelihood Ratio	.377	1	.539		
Fisher's Exact Test				.611	.363
Linear-by-Linear Association	.369	1	.544		
N of Valid Cases	66				

a. 0 cells (0.0%) have expected count less than 5. The minimum expected count is 10.18.

b. Computed only for a 2x2 table

3 – Summary and Interpretation of Analysis Results

Of the 66 participants, 24 (36.4%) worked in regional hospitals and 42 (63.6%) worked in metropolitan hospitals. In this sample, of the 38 (57.58%) participants who expressed concern for the environment, 39.47% (15/38) were from regional hospitals and 60.53% (23/38) were from metropolitan hospitals. There was no statistically significant difference in levels of environmental concern between regional participants and metropolitan participants (Fisher's Exact Test, $p = 0.611$).

Research Question: Is there an association between UK participants' role (pharmacist or pharmacy technician) and their level of environmental concern?

1- Analytical Plan

Study Design: Observational – correlational – cross-sectional

Variables:

DV: Level of environmental concern – categorical - dichotomous

IV: Role – categorical - dichotomous

Hypotheses:

H_0: There is no association between a participant's role and their level of environmental concern

H_1: There is an association between a participant's role and their level of environmental concern

Univariate Analysis:

a) DV: Level of environmental concern

Numerical summary: Frequencies (Count) and Proportion (Percentages)

Graphical summary: Bar chart

b) IV: Role

Numerical summary: Frequencies (Count) and Proportion (Percentages)

Graphical summary: Bar graph

Bivariate Analysis:

Numerical summary: Mean (sd) or Median (IQR) by group depending on normality of distribution

Graphical summary: Side-by-side box and whisker plot

Statistical Tests and Assumptions

Test: Fisher's Exact Test

Assumptions for this test are:

- Observational units are independent – assume this is so because study is cross-sectional

Significance Levels

$p < 0.05$ will be used to indicate statistical significance.

2 – Output of Analysis

a) DV: Level of environmental concern

Statistics

Env_Concern

N	Valid	40
	Missing	0

Env_Concern

		Frequency	Percent	Valid Percent	Cumulative Percent
Valid	Not concerned	24	60.0	60.0	60.0
	Concerned	16	40.0	40.0	100.0
	Total	40	100.0	100.0	

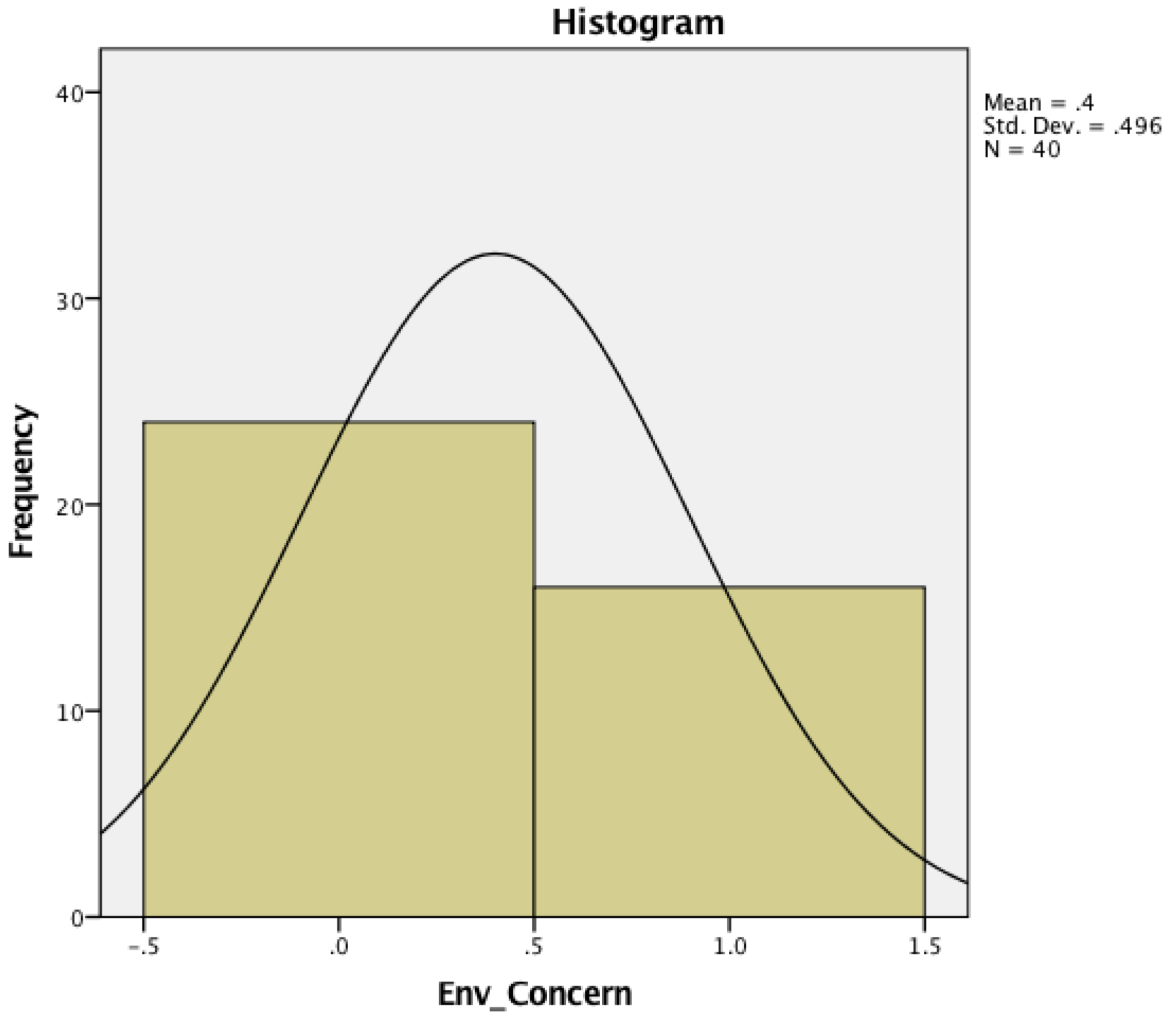

b) IV: Role

Numerical summary: Frequencies (Count) and Proportion (Percentages)

Graphical summary: Bar graph

Statistics

Role_Binary

N	Valid	40
	Missing	0

Role_Binary

		Frequency	Percent	Valid Percent	Cumulative Percent
Valid	Pharmacist	23	57.5	57.5	57.5
	Pharmacy Technician	17	42.5	42.5	100.0
	Total	40	100.0	100.0	

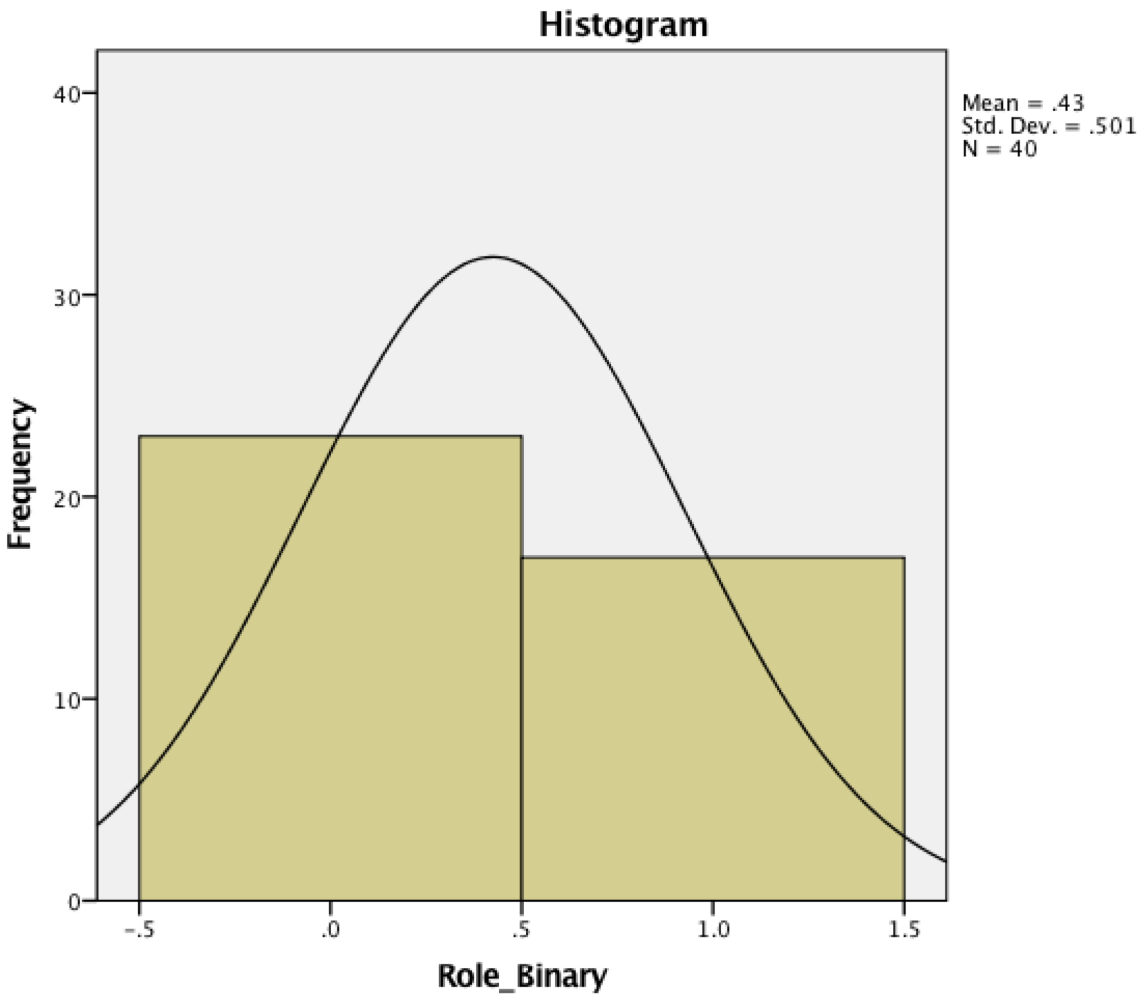

Bivariate Analysis:

Numerical summary: Mean (sd) or Median (IQR) by group depending on normality of distribution

Graphical summary: Side-by-side box and whisker plot

Descriptives

	Role_Binary			Statistic	Std. Error
Env_Concern	Pharmacist	Mean		.35	.102
		95% Confidence Interval for Mean	Lower Bound	.14	
			Upper Bound	.56	
		5% Trimmed Mean		.33	
		Median		.00	
		Variance		.237	
		Std. Deviation		.487	
		Minimum		0	
		Maximum		1	
		Range		1	
		Interquartile Range		1	
		Skewness		.684	.481
		Kurtosis		-1.687	.935
	Pharmacy Technician	Mean		.47	.125
		95% Confidence Interval for Mean	Lower Bound	.21	
			Upper Bound	.74	
		5% Trimmed Mean		.47	
		Median		.00	
		Variance		.265	
		Std. Deviation		.514	
		Minimum		0	
		Maximum		1	
		Range		1	
		Interquartile Range		1	
		Skewness		.130	.550
		Kurtosis		-2.267	1.063

Env_Concern * Role_Binary Crosstabulation

Count

		Role_Binary		
		Pharmacist	Pharmacy Technician	Total
Env_Concern	Not concerned	15	9	24
	Concerned	8	8	16
Total		23	17	40

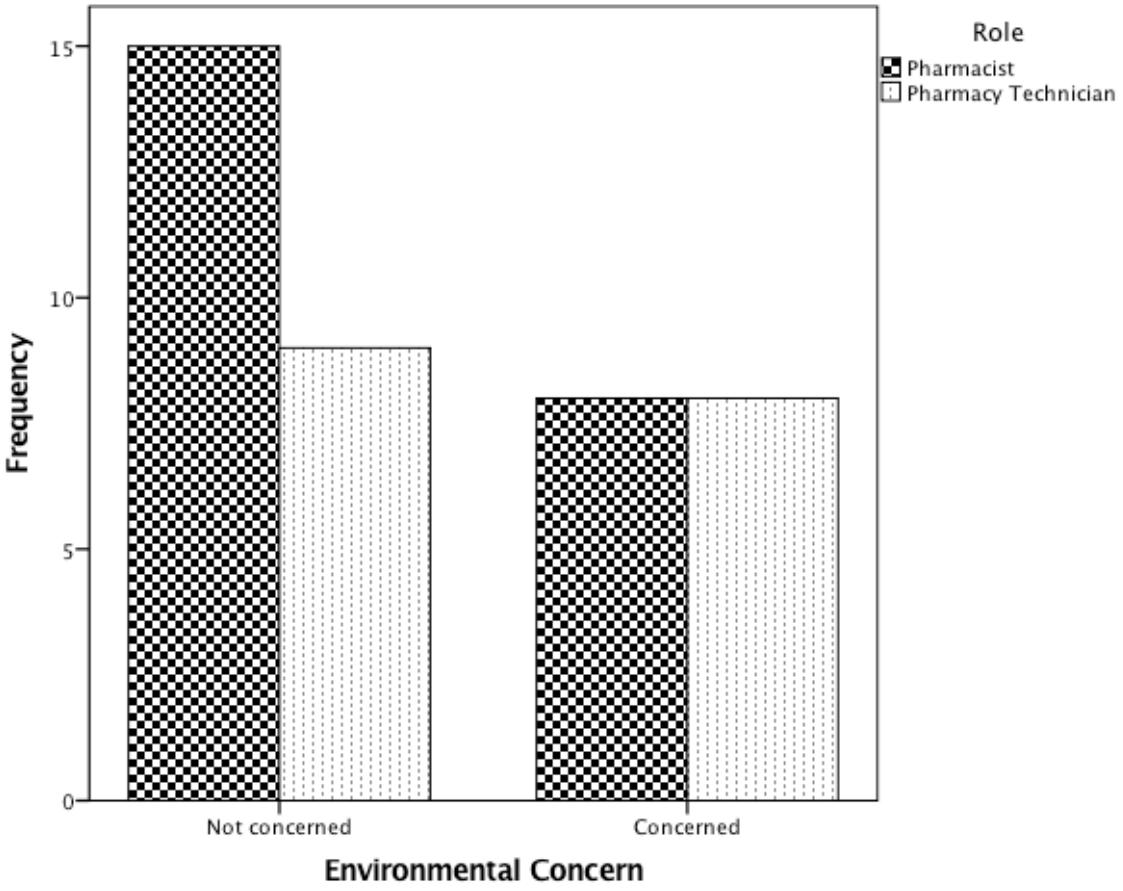

Statistical Tests and Assumptions

Test: Fisher's Exact Test

Assumptions for this test are:

- Observational units are independent – assume this is so because study is cross-sectional

Chi-Square Tests

	Value	df	Asymptotic Significance (2-sided)	Exact Sig. (2-sided)	Exact Sig. (1-sided)
Pearson Chi-Square	.614[a]	1	.433		
Continuity Correction[b]	.209	1	.648		
Likelihood Ratio	.613	1	.434		
Fisher's Exact Test				.522	.323
Linear-by-Linear Association	.598	1	.439		
N of Valid Cases	40				

a. 0 cells (0.0%) have expected count less than 5. The minimum expected count is 6.80.

b. Computed only for a 2x2 table

3 – Summary and Interpretation of Analysis Results

Of the 40 participants, 24 (60%) were not concerned about the impacts of pharmaceuticals on the environment and 16 (40%) were. Of the participants who expressed concern for the environment, 8 (50%) were pharmacists and 8 (50%) were pharmacy technicians. Of the 24 participants who were not concerned for the environment, 15 (62.5%) were pharmacists and 9 (37.5%) were pharmacy technicians. There was no statistically significant difference in concern for the environment between UK pharmacists and pharmacy technicians (Fisher's Exact test, p = 0.522).

Research Question: Is there an association between UK participants' operational level and their level of environmental concern?

1- Analytical Plan

Study Design: Observational – correlational – cross-sectional

Variables:

DV: Level of environmental concern – categorical - dichotomous

IV: Operational level – categorical - ordinal

Hypotheses:

H_0: There is no association between a participant's operational level and their level of environmental concern

H_1: There is an association between a participant's operational level and their level of environmental concern

Univariate Analysis:

a) DV: Level of environmental concern

Numerical summary: Frequencies (Count) and Proportion (Percentages)

Graphical summary: Bar chart

b) IV: Operational Level

Numerical summary: Frequencies (Count) and Proportion (Percentages)

Graphical summary: Bar graph

Bivariate Analysis:

Numerical summary: Cross-tabs

Graphical summary: Side-by-side bar chart

Statistical Tests and Assumptions

Test: Pearson's chi-square test

Assumptions for this test are:

- Observational units are independent – assume this is so because study is cross-sectional
- Expected cell counts are adequate – all expected cell counts should be $\geq$ 1 and no more than 20% of cell counts should be $\leq$ 5

Significance Levels

$p < 0.05$ will be used to indicate statistical significance.

2- Output of Analysis

Univariate Analysis:

a) DV: Level of environmental concern

Statistics

Env_Concern

N	Valid	40
	Missing	0

Env_Concern

		Frequency	Percent	Valid Percent	Cumulative Percent
Valid	Not concerned	24	60.0	60.0	60.0
	Concerned	16	40.0	40.0	100.0
	Total	40	100.0	100.0	

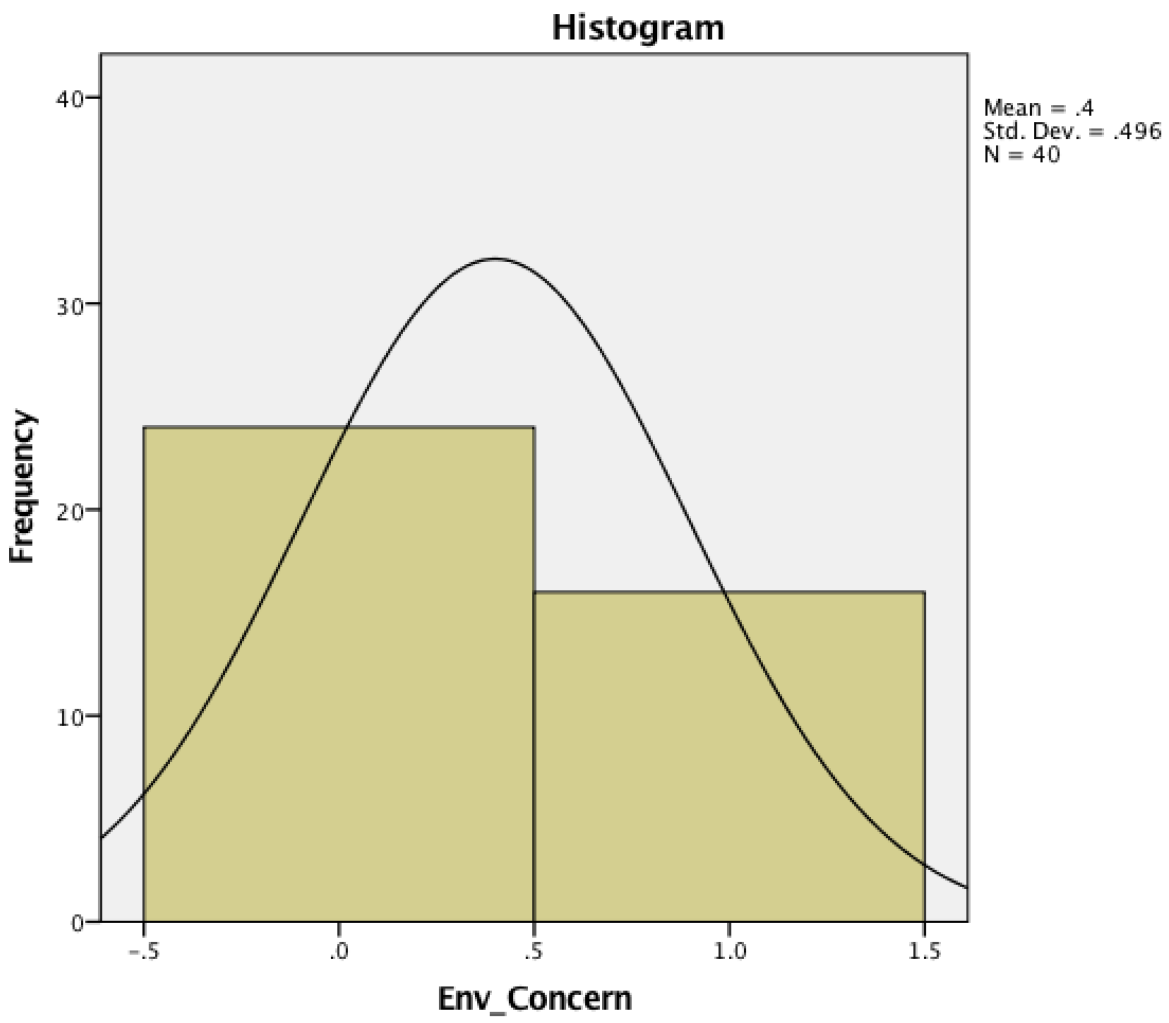

b) IV: Operational Level

Numerical summary: Frequencies (Count) and Proportion (Percentages)

Graphical summary: Bar graph

Statistics

Oper_Level

N	Valid	40
	Missing	0

Oper_Level

		Frequency	Percent	Valid Percent	Cumulative Percent
Valid	Senior Pharmacist	10	25.0	25.0	25.0
	Mid-band Pharmacist	8	20.0	20.0	45.0
	Junior Pharmacist	5	12.5	12.5	57.5
	Senior Pharmacy Technician	10	25.0	25.0	82.5
	Pharmacy Technician	7	17.5	17.5	100.0
	Total	40	100.0	100.0	

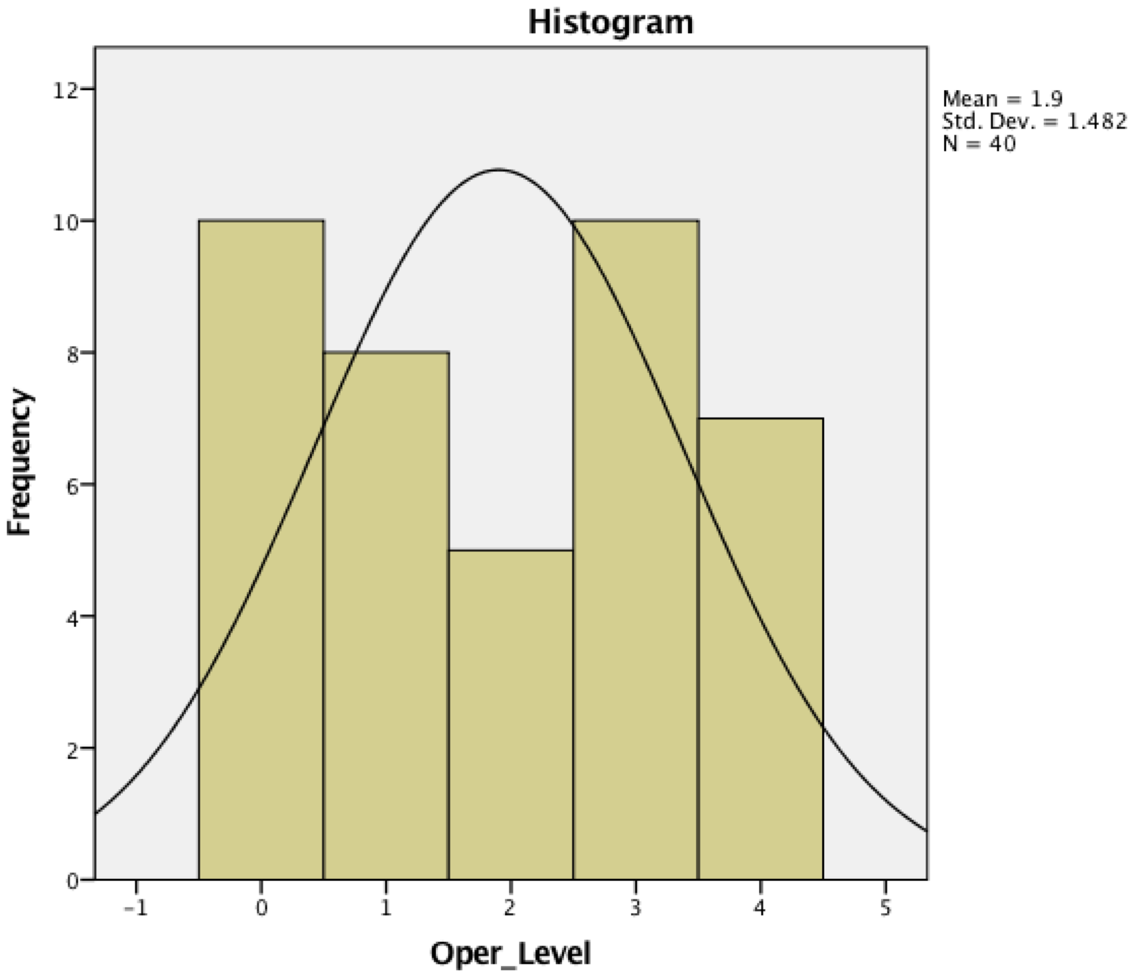

Bivariate Analysis:

Numerical summary: Mean (sd) or Median (IQR) by group depending on normality of distribution

Graphical summary: Side-by-side box and whisker plot

Env_Concern * Oper_Level Crosstabulation

Count

		Oper_Level					
		Senior Pharmacist	Mid-band Pharmacist	Junior Pharmacist	Senior Pharmacy Technician	Pharmacy Technician	Total
Env_Concern	Not concerned	7	5	3	6	3	24
	Concerned	3	3	2	4	4	16
Total		10	8	5	10	7	40

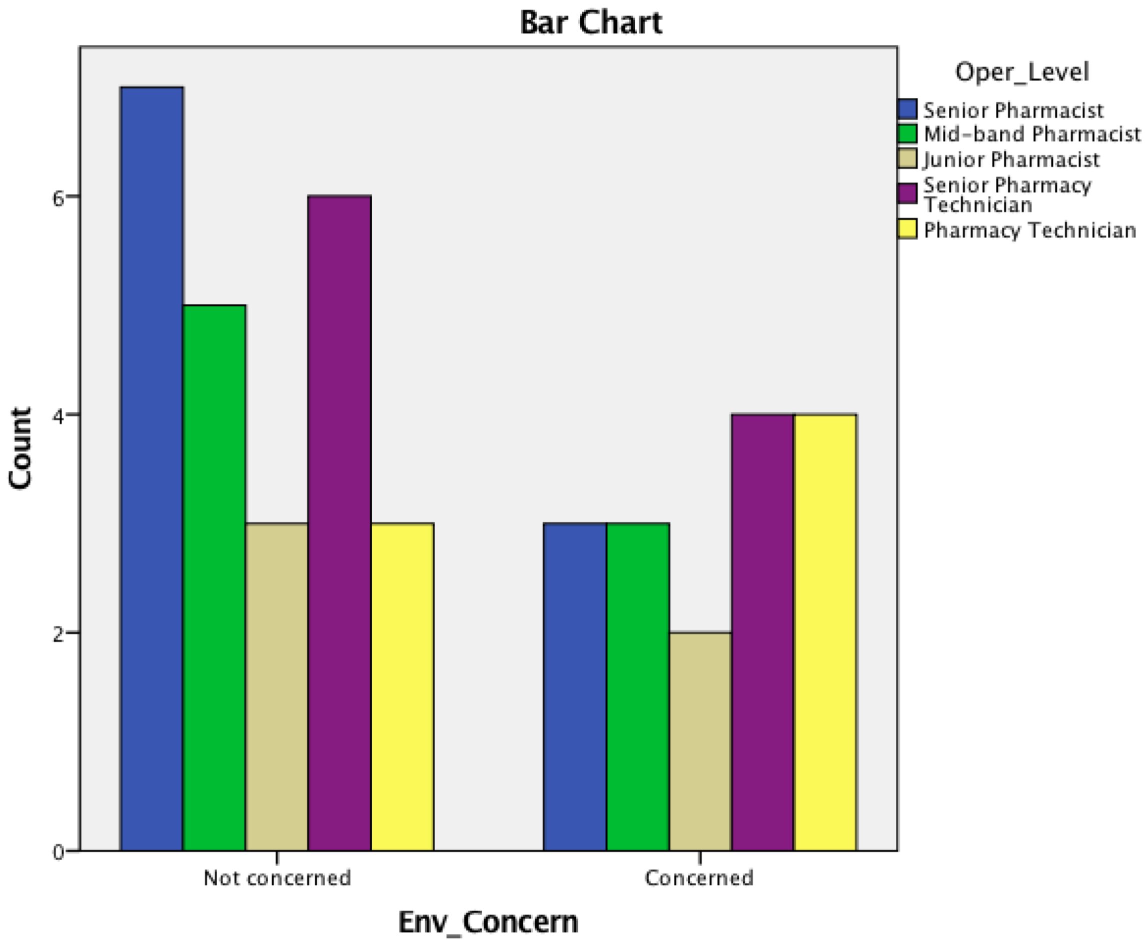

Statistical Testing

One of the assumptions of the Pearson's Chi-square test were not met – 80% of cells had cell count < 5. Therefore, some of the categories were merged.

Chi-Square Tests

	Value	df	Asymptotic Significance (2-sided)
Pearson Chi-Square	1.295[a]	4	.862
Likelihood Ratio	1.288	4	.863
Linear-by-Linear Association	1.004	1	.316
N of Valid Cases	40		

a. 8 cells (80.0%) have expected count less than 5. The minimum expected count is 2.00.

Env_Concern * OperationalLevel_2 Crosstabulation

Count

		OperationalLevel_2				Total
		.00	1.00	2.00	3.00	
Env_Concern	Not concerned	7	5	6	3	21
	Concerned	3	3	4	4	14
Total		10	8	10	7	35

Chi-Square Tests

	Value	df	Asymptotic Significance (2-sided)
Pearson Chi-Square	1.295[a]	3	.730
Likelihood Ratio	1.288	3	.732
Linear-by-Linear Association	1.104	1	.293
N of Valid Cases	35		

a. 6 cells (75.0%) have expected count less than 5. The minimum expected count is 2.80.

Merging two categories still produced 75% of cells below the expected cell count of 5. Merging further would lose the separation between senior and junior roles. Therefore, no further statistical testing was possible for this hypothesis.

3 – Summary and Interpretation of Analysis Results

Of the 40 participants, 24 (60%) expressed concern for the environment and 16 (40%) expressed no concern. The distribution of operational roles between the concerned and not concerned groups is depicted in the table below.

**Environmental Concern**	**Operational Level**					**Total**
	**Senior Pharmacist**	**Mid-Band Pharmacist**	**Junior Pharmacist**	**Senior Pharmacy Technician**	**Base-level Pharmacy Technician**	
**Concerned**	7	5	3	6	3	24
**Not Concerned**	3	3	2	4	4	16
**Total**	10	8	5	10	7	40

Statistical testing using a Pearson's chi-square test was undertaken but one of the test assumptions– no more than 20% of cell counts should be ≤ 5 – was not met. In the first run 80% of cells had cell count < 5. Therefore, two of the categories were merged. However, this still resulted in 75% of cells with a cell count < 5. Merging further would lose the separation between senior and junior roles. Therefore, no further statistical testing was possible for this hypothesis.

Research Question: Is there an association between UK participants' gender and their level of environmental concern?

1- Analytical Plan

Study Design: Observational – correlational – cross-sectional

Variables:

DV: Level of environmental concern – categorical - dichotomous

IV: Gender – categorical - dichotomous

Hypotheses:

H_0: There is no association between a participant's gender and their level of environmental concern

H_1: There is an association between a participant's gender and their level of environmental concern

Univariate Analysis:

a) DV: Level of environmental concern

Numerical summary: Frequencies (Count) and Proportion (Percentages)

Graphical summary: Bar chart

b) IV: Gender

Numerical summary: Frequencies (Count) and Proportion (Percentages)

Graphical summary: Bar graph

Bivariate Analysis:

Cross-tabs

Statistical Tests and Assumptions

Test: Fisher's Exact test

Assumptions for this test are:

- Observational units are independent – assume this is so because study is cross-sectional

Significance Levels

$p < 0.05$ will be used to indicate statistical significance.

2 – Output of Analysis

Univariate Analysis:

a) DV: Level of environmental concern

See above

b) IV: Gender

Numerical summary: Frequencies (Count) and Proportion (Percentages)

Graphical summary: Bar graph

Gender

		Frequency	Percent	Valid Percent	Cumulative Percent
Valid	Male	18	45.0	45.0	45.0
	Female	22	55.0	55.0	100.0
	Total	40	100.0	100.0	

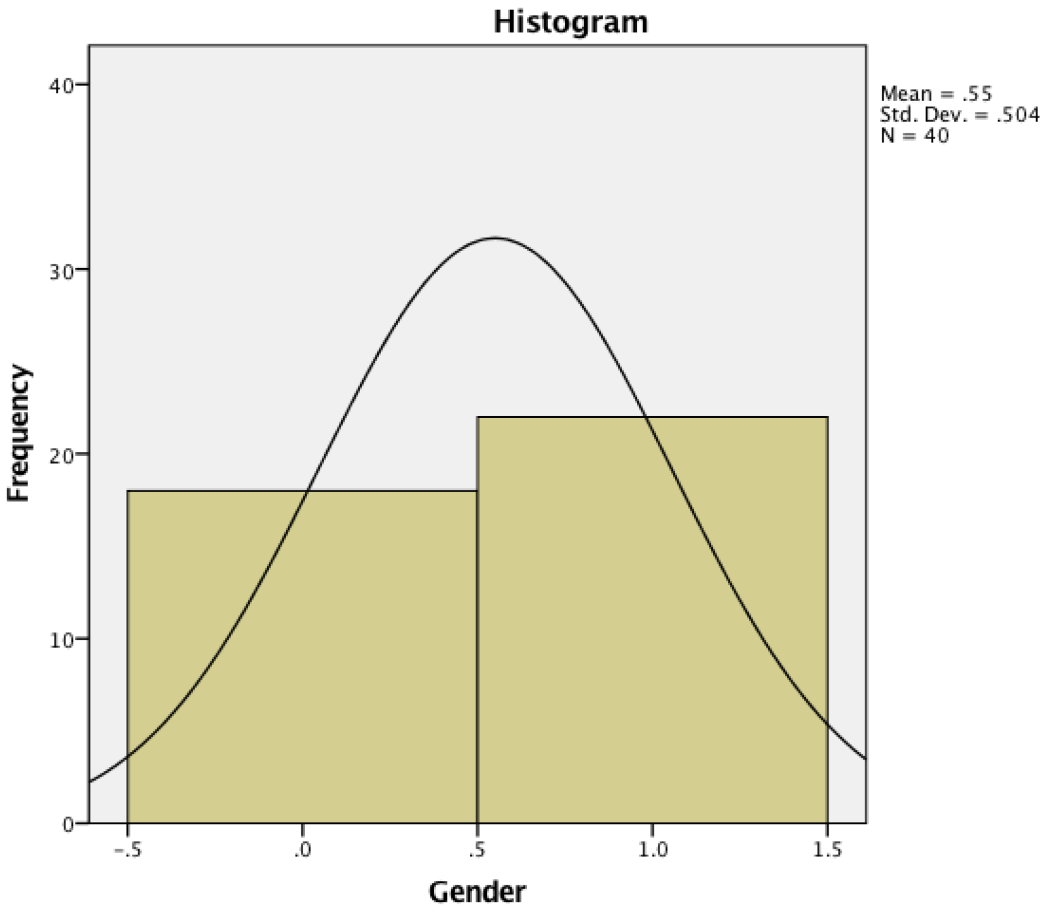

Bivariate Analysis

Env_Concern * Gender Crosstabulation

Count

		Gender		Total
		Male	Female	
Env_Concern	Not concerned	10	14	24
	Concerned	8	8	16
Total		18	22	40

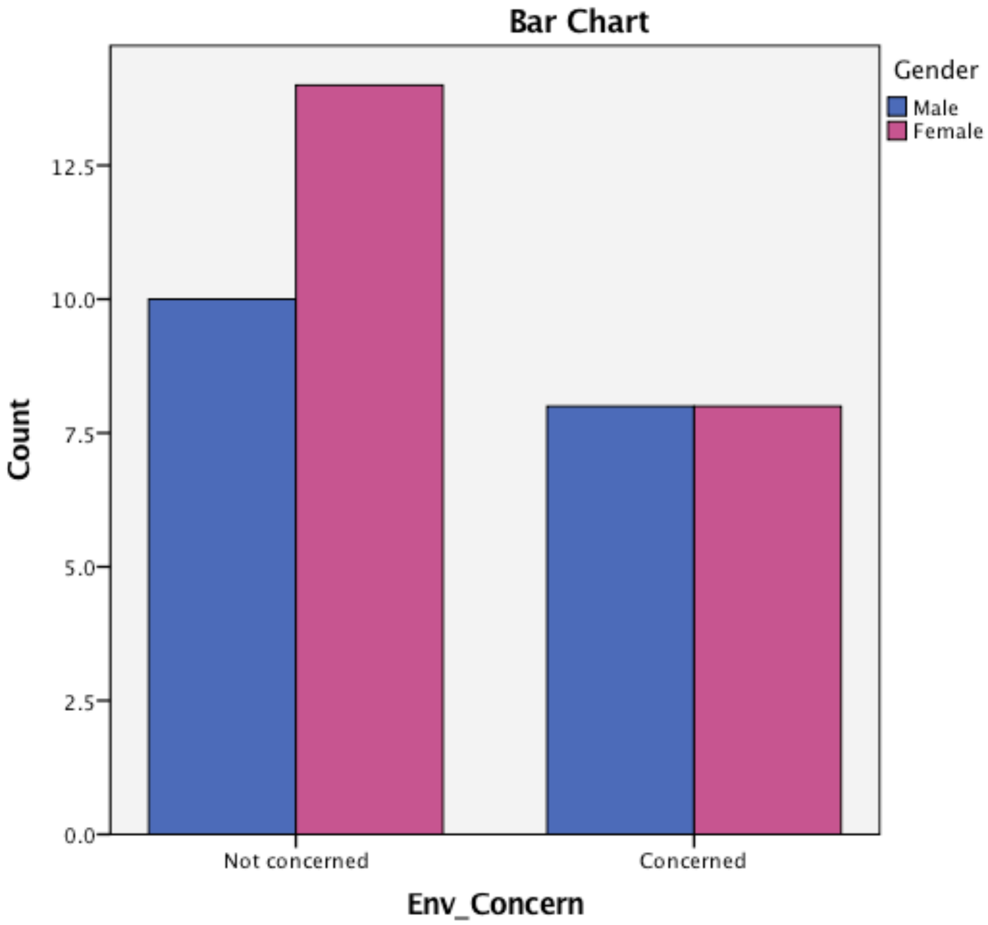

Statistical Testing

Chi-Square Tests

	Value	df	Asymptotic Significance (2-sided)	Exact Sig. (2-sided)	Exact Sig. (1-sided)
Pearson Chi-Square	.269[a]	1	.604		
Continuity Correction[b]	.038	1	.846		
Likelihood Ratio	.269	1	.604		
Fisher's Exact Test				.748	.422
Linear-by-Linear Association	.263	1	.608		
N of Valid Cases	40				

a. 0 cells (0.0%) have expected count less than 5. The minimum expected count is 7.20.

b. Computed only for a 2x2 table

There was no statistically significant difference in environmental concern between male and female participants (Fisher's Exact Test, p = 0.748).

3 – Summary and Interpretation of Analysis Results

Of the 40 participants, 18 (35%) were male and 22 (55%) were female. Of the 18 male participants, 10 (55.56%) were not concerned about the environment and 8 (44.44%) were concerned. Of the 22 female participants, 14 (63.64%) were not concerned about the environment whereas 8 (36.36%) were concerned. There was no statistically significant difference in environmental concern between male and female participants (Fisher's Exact Test, p = 0.748).

Research Question: Is there an association between UK participants' city and their level of environmental concern?

1- Analytical Plan

Study Design: Observational – correlational – cross-sectional

Variables:

DV: Level of environmental concern – categorical - dichotomous

IV: Gender – categorical - dichotomous

Hypotheses:

H_0: There is no association between a participant's gender and their level of environmental concern

H_1: There is an association between a participant's gender and their level of environmental concern

Univariate Analysis:

a) DV: Level of environmental concern

Numerical summary: Frequencies (Count) and Proportion (Percentages)

Graphical summary: Bar chart

b) IV: Region

Numerical summary: Frequencies (Count) and Proportion (Percentages)

Graphical summary: Bar graph

Bivariate Analysis:

Numerical: Cross-tabulation

Graphical: bar charts

Statistical Tests and Assumptions

Test: Pearson's chi-square test

Assumptions for this test are:

- Observational units are independent – assume this is so because study is cross-sectional
- Expected cell counts are adequate – all expected cell counts should be $\geq$ 1 and no more than 20% of cell counts should be $\leq$ 5

Significance Levels

$p < 0.05$ will be used to indicate statistical significance.

2 – Output of Analysis

Univariate Analysis:

a) DV: Level of environmental concern

See above

b) IV: City

Numerical summary: Frequencies (Count) and Proportion (Percentages)

Graphical summary: Bar graph

Statistics

City

N	Valid	40
	Missing	0

City

		Frequency	Percent	Valid Percent	Cumulative Percent
Valid	Cambridge	6	15.0	15.0	15.0
	Leeds	17	42.5	42.5	57.5
	Sheffield	9	22.5	22.5	80.0
	York	8	20.0	20.0	100.0
	Total	40	100.0	100.0	

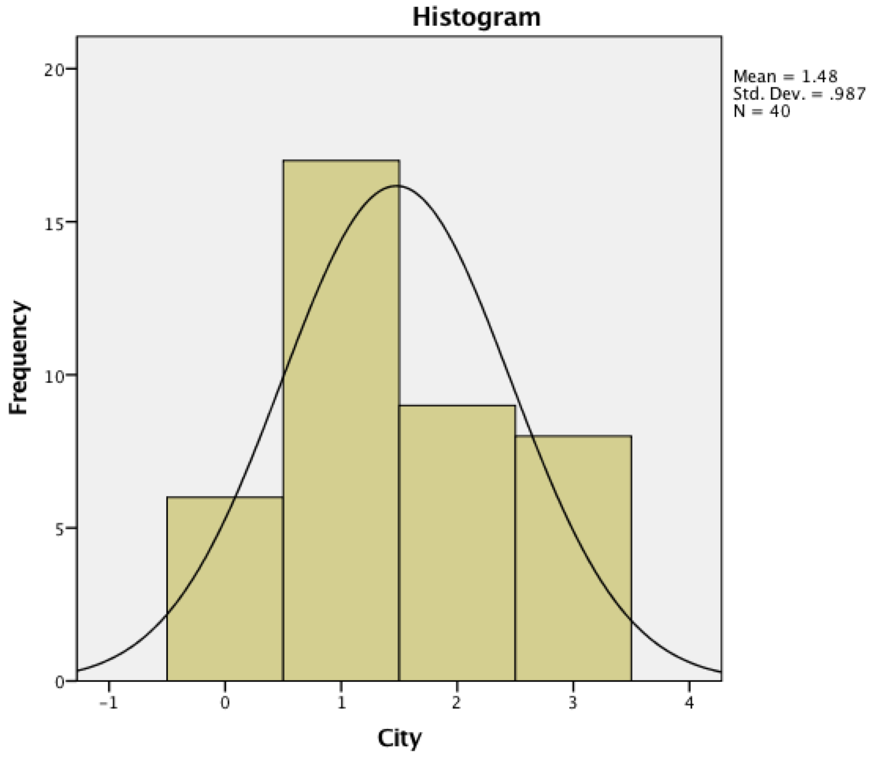

Bivariate Analysis

Env_Concern * City Crosstabulation

Count

		City				Total
		Cambridge	Leeds	Sheffield	York	
Env_Concern	Not concerned	3	10	7	4	24
	Concerned	3	7	2	4	16
Total		6	17	9	8	40

Statistical Testing

Chi-Square Tests

	Value	df	Asymptotic Significance (2-sided)
Pearson Chi-Square	1.778[a]	3	.620
Likelihood Ratio	1.863	3	.601
Linear-by-Linear Association	.039	1	.844
N of Valid Cases	40		

a. 5 cells (62.5%) have expected count less than 5. The minimum expected count is 2.40.

3 – Summary and Interpretation of Analysis Results

Of the 40 participants, six worked in Cambridge, 17 worked in Leeds, nine worked in Sheffield and eight worked in York. The distribution of concerned and not concerned participants across the cities is depicted in the table below.

**Environmental Concern**	**City**				**Total**
	Cambridge	Leeds	Sheffield	York	
**Concerned**	3	7	2	4	16
**Not Concerned**	3	10	7	4	24
**Total**	6	17	9	8	40

Statistical testing was undertaken using Pearson's chi-square test however one of the assumptions (no more than 20% of cell counts should be $\leq$ 5) was not met. Being different cities, these categories could not be merged to increase the cell counts and therefore statistical testing was not possible for this hypothesis.

Research Question: Is environmental attitude a predictor of level of environmental concern in Australian Participants?

1- Analytical Plan

Study Design: Observational – correlational – cross-sectional

Variables:

DV: Level of environmental concern (categorical – dichotomous)

IV: NEP score

Hypotheses:

H_0: There is no association between level of environmental concern and environmental attitude

H_1: There is an association between level of environmental concern and environmental attitude

Univariate Analysis:

a) DV: Level of environmental concern

Numerical summary: Frequencies (Count) and Proportion (Percentages)

Graphical summary: Bar chart

b) IV: Mean NEP Score

Numerical summary: Frequencies (Count) and Proportion (Percentages)

Graphical summary: Bar graph

Bivariate Analysis:

Numerical summary: Mean (sd) or Median (IQR) by group depending on normality of distribution

Graphical summary: Side-by-side box and whisker plot

Statistical Tests and Assumptions

Test: Logistic regression

Assumptions for this test are:

See over page:

Significance Levels

$p < 0.05$ will be used to indicate statistical significance.

Assumptions for Binary Logistic Regression

Binary logistic regression requires the dependent variable to be binary and ordinal logistic regression requires the dependent variable to be ordinal. Reducing an ordinal or even metric variable to dichotomous level loses a lot of information, which makes this test inferior compared to ordinal logistic regression in these cases.

Secondly, since logistic regression assumes that P(Y=1) is the probability of the event occurring, it is necessary that the dependent variable is coded accordingly. That is, for a binary regression, the factor level 1 of the dependent variable should represent the desired outcome.

Thirdly, the model should be fitted correctly. Neither over fitting nor under fitting should occur. That is only the meaningful variables should be included, but also all meaningful variables should be included. A good approach to ensure this is to use a stepwise method to estimate the logistic regression.

Fourthly, the error terms need to be independent. Logistic regression requires each observation to be independent. That is that the data-points should not be from any dependent samples design, e.g., before-after measurements, or matched pairings. Also the model should have little or no multicollinearity. That is that the independent variables should be independent from each other. However, there is the option to include interaction effects of categorical variables in the analysis and the model. If multicollinearity is present centering the variables might resolve the issue, i.e. deducting the mean of each variable. If this does not lower the multicollinearity, a factor analysis with orthogonally rotated factors should be done before the logistic regression is estimated.

Fifthly, logistic regression assumes linearity of independent variables and log odds. Whilst it does not require the dependent and independent variables to be related linearly, it requires that the independent variables are linearly related to the log odds. Otherwise the test underestimates the strength of the relationship and rejects the relationship too easily, that is being not significant (not rejecting the null hypothesis) where it should be significant. A solution to this problem is the categorization of the independent variables. That is transforming metric variables to ordinal level and then including them in the model. Another approach would be to use discriminant analysis, if the assumptions of homoscedasticity, multivariate normality, and absence of multicollinearity are met.

Lastly, it requires quite large sample sizes. Because maximum likelihood estimates are less powerful than ordinary least squares (e.g., simple linear regression, multiple linear regression); whilst OLS needs 5 cases per independent variable in the analysis, ML needs at least 10 cases per independent variable, some statisticians recommend at least 30 cases for each parameter to be estimated.

2 - Output of Analysis

Univariate Analysis:

a) DV: Level of environmental concern

Numerical summary: Frequencies (Count) and Proportion (Percentages)

Graphical summary: Bar chart

Frequencies

Statistics

EnvironmentalConcern

N	Valid	66
	Missing	0

EnvironmentalConcern

		Frequency	Percent	Valid Percent	Cumulative Percent
Valid	Concerned	38	57.6	57.6	57.6
	Not concerned	28	42.4	42.4	100.0
	Total	66	100.0	100.0	

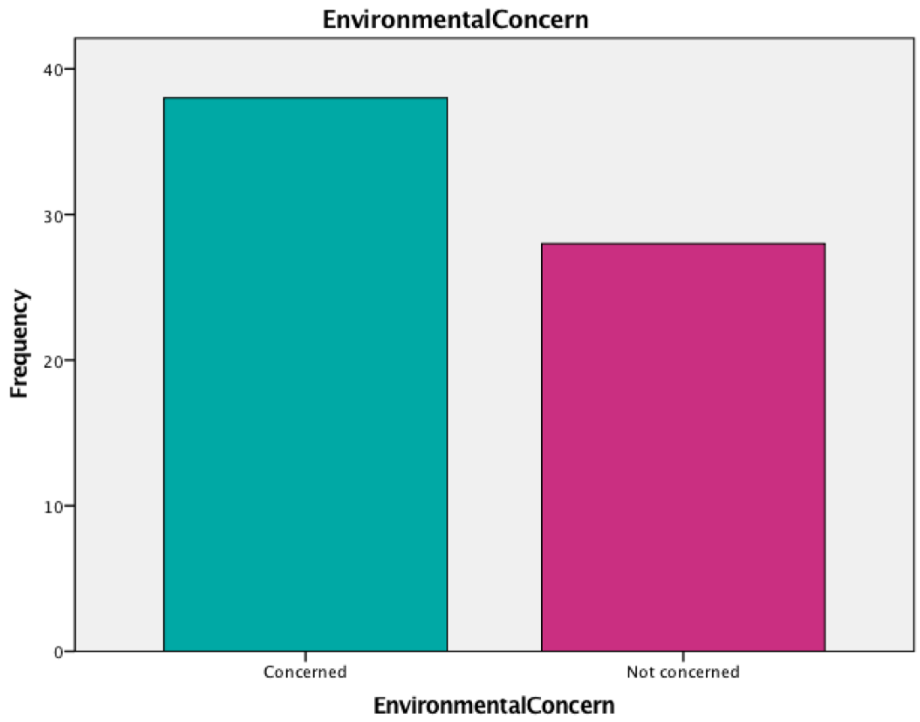

b) IV: NEP Score

See page 7

Bivariate Analysis:

Numerical summary: Mean (sd) or Median (IQR) by group depending on normality of distribution

Graphical summary: Side-by-side box and whisker plot

EnvironmentalConcern

Case Processing Summary

	EnvironmentalConcern	Cases					
		Valid		Missing		Total	
		N	Percent	N	Percent	N	Percent
Nep_Tot	Concerned	38	100.0%	0	0.0%	38	100.0%
	Not concerned	28	100.0%	0	0.0%	28	100.0%

Descriptives

	EnvironmentalConcern			Statistic	Std. Error
Nep_Tot	Concerned	Mean		56.08	1.141
		95% Confidence Interval for Mean	Lower Bound	53.77	
			Upper Bound	58.39	
		5% Trimmed Mean		56.07	
		Median		55.00	
		Variance		49.480	
		Std. Deviation		7.034	
		Minimum		38	
		Maximum		71	
		Range		33	
		Interquartile Range		7	
		Skewness		.093	.383
		Kurtosis		.399	.750
	Not concerned	Mean		52.54	1.188
		95% Confidence Interval for Mean	Lower Bound	50.10	
			Upper Bound	54.97	
		5% Trimmed Mean		52.23	
		Median		52.00	
		Variance		39.517	
		Std. Deviation		6.286	
		Minimum		40	
		Maximum		72	
		Range		32	
		Interquartile Range		6	
		Skewness		.894	.441
		Kurtosis		2.547	.858

Looking at normality of distributions:

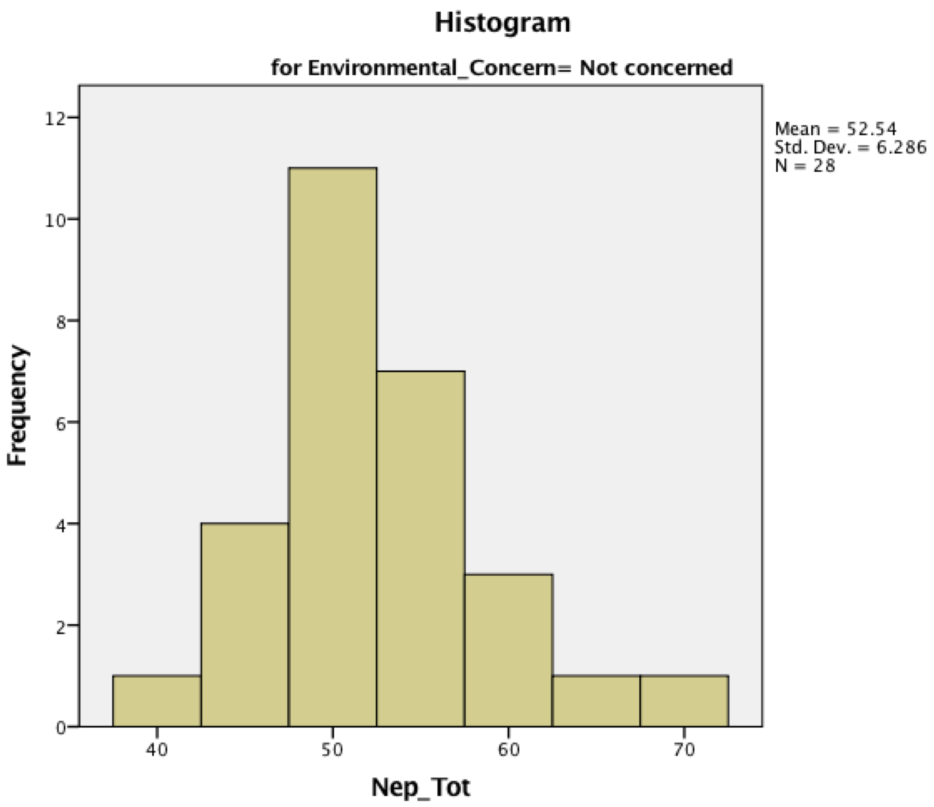
Histogram
for Environmental_Concern= Not concerned
Mean = 52.54
Std. Dev. = 6.286
N = 28
Frequency
0
2
4
6
8
10
12
40
50
60
70
Nep_Tot

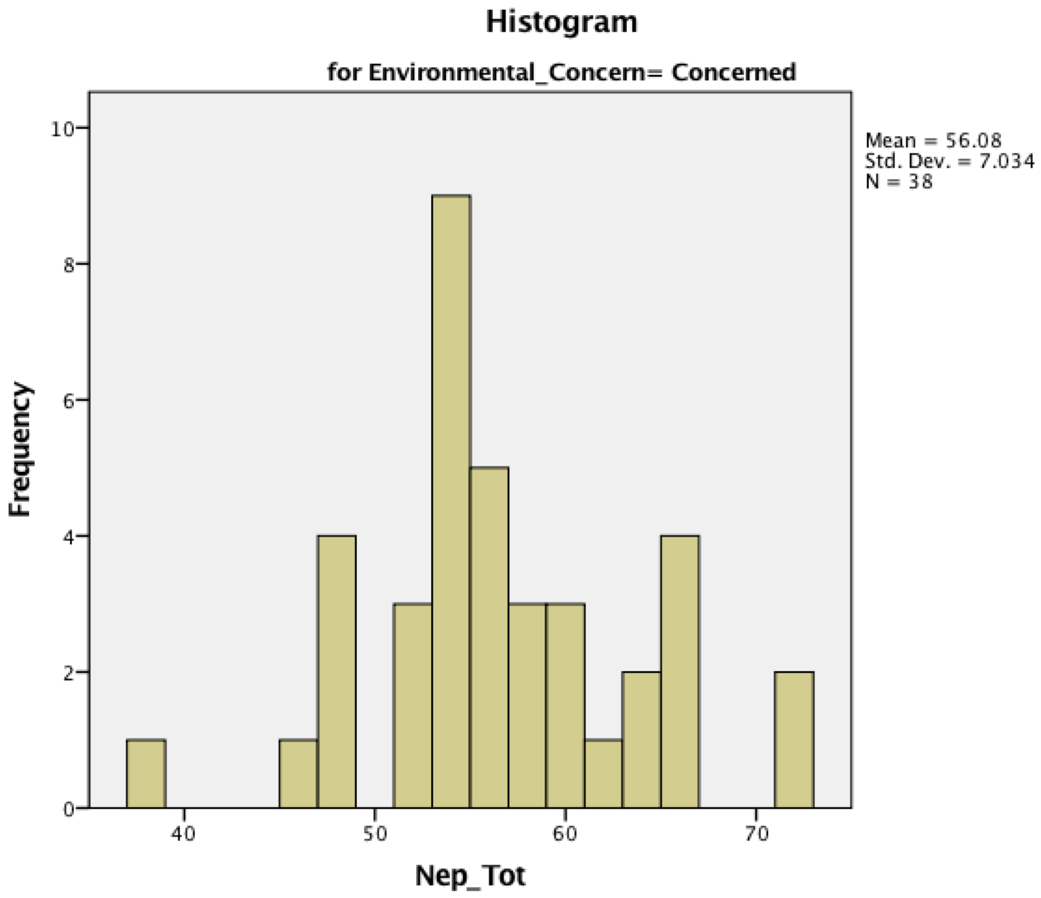
Histogram
for Environmental_Concern= Concerned
Mean = 56.08
Std. Dev. = 7.034
N = 38
Frequency
0
2
4
6
8
10
40
50
60
70
Nep_Tot

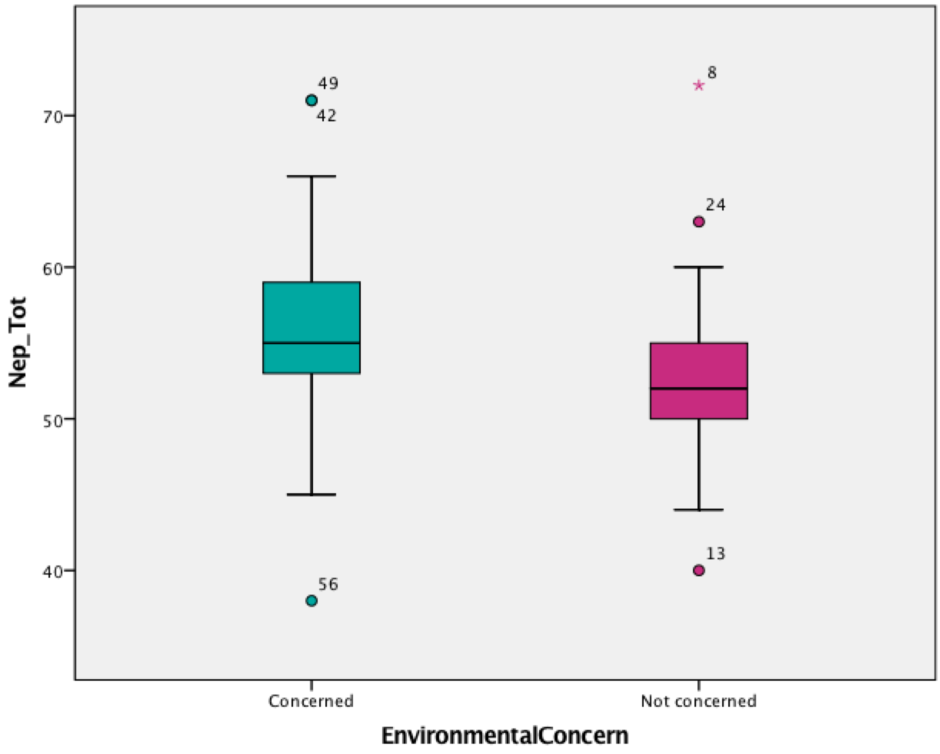

Statistical Tests and Assumptions

A binary logistic regression was performed.

Logistic Regression

Case Processing Summary

Unweighted Cases[a]		N	Percent
Selected Cases	Included in Analysis	66	100.0
	Missing Cases	0	.0
	Total	66	100.0
Unselected Cases		0	.0
Total		66	100.0

a. If weight is in effect, see classification table for the total number of cases.

Dependent Variable Encoding

Original Value	Internal Value
Concerned	0
Not concerned	1

Block 0: Beginning Block

Classification Table[a,b]

Observed			Predicted: EnvironmentalConcern – Concerned	Predicted: EnvironmentalConcern – Not concerned	Percentage Correct
Step 0	EnvironmentalConcern	Concerned	38	0	100.0
		Not concerned	28	0	.0
	Overall Percentage				57.6

a. Constant is included in the model.

b. The cut value is .500

Variables in the Equation

		B	S.E.	Wald	df	Sig.	Exp(B)
Step 0	Constant	-.305	.249	1.503	1	.220	.737

Variables not in the Equation

			Score	df	Sig.
Step 0	Variables	Nep_Tot	4.309	1	.038
	Overall Statistics		4.309	1	.038

Block 1: Method = Enter

Omnibus Tests of Model Coefficients

		Chi-square	df	Sig.
Step 1	Step	4.530	1	.033
	Block	4.530	1	.033
	Model	4.530	1	.033

Model Summary

Step	-2 Log likelihood	Cox & Snell R Square	Nagelkerke R Square
1	85.444[a]	.066	.089

a. Estimation terminated at iteration number 4 because parameter estimates changed by less than .001.

Hosmer and Lemeshow Test

Step	Chi-square	df	Sig.
1	10.848	7	.145

Contingency Table for Hosmer and Lemeshow Test

		EnvironmentalConcern = Concerned		EnvironmentalConcern = Not concerned		
		Observed	Expected	Observed	Expected	Total
Step 1	1	6	5.648	1	1.352	7
	2	6	5.640	2	2.360	8
	3	3	3.859	3	2.141	6
	4	5	3.626	1	2.374	6
	5	3	2.854	2	2.146	5
	6	6	4.953	3	4.047	9
	7	3	3.708	4	3.292	7
	8	0	3.459	7	3.541	7
	9	6	4.253	5	6.747	11

Classification Table[a]

			Predicted		
			EnvironmentalConcern		
Observed			Concerned	Not concerned	Percentage Correct
Step 1	EnvironmentalConcern	Concerned	32	6	84.2
		Not concerned	19	9	32.1
	Overall Percentage				62.1

a. The cut value is .500

Variables in the Equation

		B	S.E.	Wald	df	Sig.	Exp(B)	95% C.I.for EXP(B) Lower	95% C.I.for EXP(B) Upper
Step 1[a]	Nep_Tot	-.083	.042	3.982	1	.046	.920	.848	.999
	Constant	4.202	2.259	3.458	1	.063	66.794		

a. Variable(s) entered on step 1: Nep_Tot.

3 – Summary of Results

Looking at Omnibus Test of Model Coefficients, p = 0.033 which is statistically significant and therefore a participant's environmental attitude influences their level of environmental concern – report as (χ^2_1= 4.530, p = 0.033) which indicates that the model was able to distinguish between participants who were concerned and those who were not. The model explained between 6.6% (Cox and Snell R Square) and 8.9% (Nagelkerke R Square) of variance in environmental concern.

The Odd's Ratio = Exp(B) = 0.920

If value is > 1 then we would say that as the predictor variable increases (environmental attitude) then the odds of the outcome occurring (increase in level of environmental concern) will increase. Close enough to 1 to say that as environmental attitudinal score increases the odds of level of environmental concern rising increases by 1.

Research Question: Is environmental attitude a predictor of level of environmental concern in UK participants?

1- Analytical Plan

Study Design: Observational – correlational – cross-sectional

Variables:

DV: Level of environmental concern (categorical – dichotomous)

IV: NEP score

Hypotheses:

H_0: There is no association between level of environmental concern and environmental attitude

H_1: There is an association between level of environmental concern and environmental attitude

Univariate Analysis:

a) DV: Level of environmental concern

Numerical summary: Frequencies (Count) and Proportion (Percentages)

Graphical summary: Bar chart

b) IV: Mean NEP Score

Numerical summary: Frequencies (Count) and Proportion (Percentages)

Graphical summary: Bar chart

Bivariate Analysis:

Numerical summary: Mean (sd) or Median (IQR) by group depending on normality of distribution

Graphical summary: Side-by-side box and whisker plot

Statistical Tests and Assumptions

Test: Logistic regression

Assumptions for this test are: see over page

Significance Levels: $p < 0.05$ will be used to indicate statistical significance.

Assumptions for Binary Logistic Regression

Binary logistic regression requires the dependent variable to be binary and ordinal logistic regression requires the dependent variable to be ordinal. Reducing an ordinal or even metric variable to dichotomous level loses a lot of information, which makes this test inferior compared to ordinal logistic regression in these cases.

Secondly, since logistic regression assumes that P(Y=1) is the probability of the event occurring, it is necessary that the dependent variable is coded accordingly. That is, for a binary regression, the factor level 1 of the dependent variable should represent the desired outcome.

Thirdly, the model should be fitted correctly. Neither over fitting nor under fitting should occur. That is only the meaningful variables should be included, but also all meaningful variables should be included. A good approach to ensure this is to use a stepwise method to estimate the logistic regression.

Fourthly, the error terms need to be independent. Logistic regression requires each observation to be independent. That is that the data-points should not be from any dependent samples design, e.g., before-after measurements, or matched pairings. Also the model should have little or no multicollinearity. That is that the independent variables should be independent from each other. However, there is the option to include interaction effects of categorical variables in the analysis and the model. If multicollinearity is present centering the variables might resolve the issue, i.e. deducting the mean of each variable. If this does not lower the multicollinearity, a factor analysis with orthogonally rotated factors should be done before the logistic regression is estimated.

Fifthly, logistic regression assumes linearity of independent variables and log odds. Whilst it does not require the dependent and independent variables to be related linearly, it requires that the independent variables are linearly related to the log odds. Otherwise the test underestimates the strength of the relationship and rejects the relationship too easily, that is being not significant (not rejecting the null hypothesis) where it should be significant. A solution to this problem is the categorization of the independent variables. That is transforming metric variables to ordinal level and then including them in the model. Another approach would be to use discriminant analysis, if the assumptions of homoscedasticity, multivariate normality, and absence of multicollinearity are met.

Lastly, it requires quite large sample sizes. Because maximum likelihood estimates are less powerful than ordinary least squares (e.g., simple linear regression, multiple linear regression); whilst OLS needs 5 cases per independent variable in the analysis, ML needs at least 10 cases per independent variable, some statisticians recommend at least 30 cases for each parameter to be estimated.

2 - Output of Analysis

Univariate Analysis:

a) DV: Level of environmental concern

Numerical summary: Frequencies (Count) and Proportion (Percentages)

Graphical summary: Bar chart

Frequencies

Statistics

Env_Concern

N	Valid	40
	Missing	0

Env_Concern

		Frequency	Percent	Valid Percent	Cumulative Percent
Valid	Not concerned	24	60.0	60.0	60.0
	Concerned	16	40.0	40.0	100.0
	Total	40	100.0	100.0	

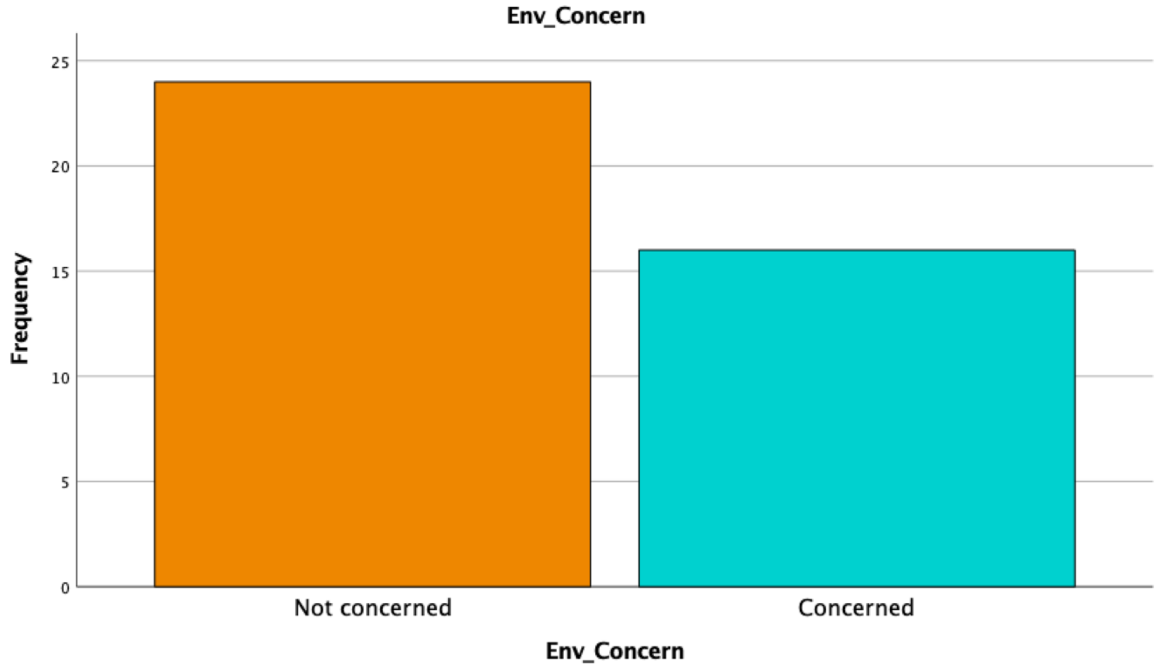

b) IV: NEP Score

Reliability

[DataSet1] /Users/judithsingleton/

Scale: NEP_Tot

Case Processing Summary

		N	%
Cases	Valid	40	100.0
	Excluded[a]	0	.0
	Total	40	100.0

a. Listwise deletion based on all variables in the procedure.

Reliability Statistics

Cronbach's Alpha	N of Items
.734	15

Frequencies

Statistics

NEP_Tot

N	Valid	40
	Missing	0
Mean		54.10
Median		55.00
Std. Deviation		6.042
Variance		36.503
Skewness		-.327
Std. Error of Skewness		.374
Kurtosis		-.267
Std. Error of Kurtosis		.733
Range		26

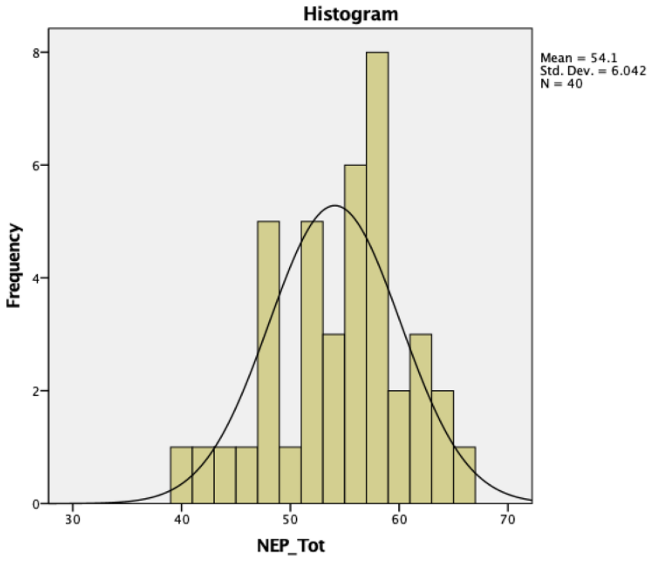

Bivariate Analysis:

Numerical summary: Mean (sd) or Median (IQR) by group depending on normality of distribution

Graphical summary: Side-by-side box and whisker plot

Env_Concern

Case Processing Summary

	Env_Concern	Valid N	Valid Percent	Missing N	Missing Percent	Total N	Total Percent
		Cases					
NEP_Tot	Not concerned	24	100.0%	0	0.0%	24	100.0%
	Concerned	16	100.0%	0	0.0%	16	100.0%

Descriptives

	Env_Concern			Statistic	Std. Error
NEP_Tot	Not concerned	Mean		53.04	1.133
		95% Confidence Interval for Mean	Lower Bound	50.70	
			Upper Bound	55.39	
		5% Trimmed Mean		53.19	
		Median		53.50	
		Variance		30.824	
		Std. Deviation		5.552	
		Minimum		40	
		Maximum		63	
		Range		23	
		Interquartile Range		9	
		Skewness		-.445	.472
		Kurtosis		-.063	.918
	Concerned	Mean		55.69	1.642
		95% Confidence Interval for Mean	Lower Bound	52.19	
			Upper Bound	59.19	
		5% Trimmed Mean		55.87	
		Median		57.00	
		Variance		43.163	
		Std. Deviation		6.570	
		Minimum		42	
		Maximum		66	
		Range		24	
		Interquartile Range		9	
		Skewness		-.520	.564
		Kurtosis		-.139	1.091

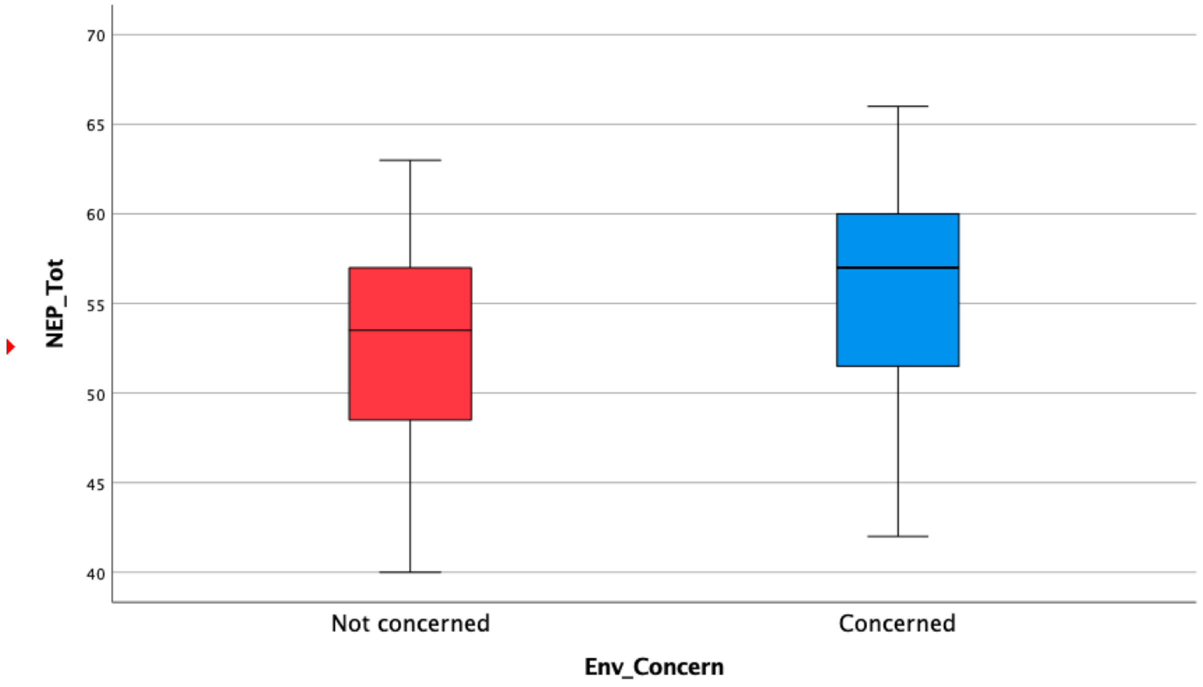

Looking at normality of distributions:

Proven normality – see previous tests.

Statistical Tests and Assumptions

A binary logistic regression was performed.

Logistic Regression

Case Processing Summary

Unweighted Cases[a]		N	Percent
Selected Cases	Included in Analysis	40	100.0
	Missing Cases	0	.0
	Total	40	100.0
Unselected Cases		0	.0
Total		40	100.0

a. If weight is in effect, see classification table for the total number of cases.

Dependent Variable Encoding

Original Value	Internal Value
Not concerned	0
Concerned	1

Block 0: Beginning Block

Classification Table[a,b]

Observed			Predicted: Env_Concern: Not concerned	Predicted: Env_Concern: Concerned	Percentage Correct
Step 0	Env_Concern	Not concerned	24	0	100.0
		Concerned	16	0	.0
	Overall Percentage				60.0

a. Constant is included in the model.

b. The cut value is .500

Variables in the Equation

		B	S.E.	Wald	df	Sig.	Exp(B)
Step 0	Constant	-.405	.323	1.578	1	.209	.667

Block 1: Method = Enter

Omnibus Tests of Model Coefficients

		Chi-square	df	Sig.
Step 1	Step	25.748	20	.174
	Block	25.748	20	.174
	Model	25.748	20	.174

Model Summary

Step	-2 Log likelihood	Cox & Snell R Square	Nagelkerke R Square
1	28.093[a]	.475	.642

a. Estimation terminated at iteration number 20 because maximum iterations has been reached. Final solution cannot be found.

Hosmer and Lemeshow Test

Step	Chi-square	df	Sig.
1	.000	4	1.000

3 – Summary of Results

Looking at Omnibus Test of Model Coefficients, p = 0.174 which is the 'goodness of fit' test for our model and indicates that our model is not a good fit. Wald test, p=0.209 indicating that NEP score (environmental attitude) did not predict level of environmental concern in participants.
