# Supplementary material for: Do legislated carbon reduction targets influence pro-environmental behaviours in public hospital pharmacy departments? Using mixed methods to compare Australia and the UK
Source: PLoS One. 2021 Aug 18;16(8):e0255445. doi: 10.1371/journal.pone.0255445 (PMC8372918; doi:10.1371/journal.pone.0255445)
Supplement: S6 File — (DOCX) [file pone.0255445.s014.docx]

| **VARIABLE NAME** | **DESCRIPTION** | **CODING** |
| --- | --- | --- |
| ID | Unique participant ID | Numeric value 999=missing data |
| Country | Country where participant worked | 0=Australia  1=UK  999=missing |
| Gender | Gender of participants | 0=male  1=female  *‘other’ was also an option; no participants selected this option so variable was computed as dichotomous |
| Role_Binary | Role of participant | 0= pharmacist  1=pharmacy technician  999=missing data |
| NEP_Sc_Cat | Environmental Attitude Category | 0=<39 Anti-environmental  1=39-58 Mid-environmental  2=>58 Pro-environmental  999=missing data |
| NEP_Tot | Participants’ environmental attitude score | Numeric value  999-missing data |
| Env_Concern | Participants’ level of environmental concern | 0=not concerned  1=concerned  999=missing data |
| Self_Concord | Green goals are self-concordant goals | 0=no  1=yes  999=missing data |
| Oper_Level | Participants’ operational level | 0=senior pharmacist  1=mid-band pharmacist  2=junior pharmacist  3=senior pharmacy technician  4=pharmacy technician  5=junior pharmacy technician  999=missing data |
| Q1 | NEP Scale Question 1 | Numeric Value  999=missing data |
| Q2 | NEP Scale Question 2 | Numeric Value  999=missing data |
| Q3 | NEP Scale Question 3 | Numeric Value  999=missing data |
| Q4 | NEP Scale Question 4 | Numeric Value  999=missing data |
| Q5 | NEP Scale Question 5 | Numeric Value  999=missing data |
| Q6 | NEP Scale Question 6 | Numeric Value  999=missing data |
| Q7 | NEP Scale Question 7 | Numeric Value  999=missing data |
| Q8 | NEP Scale Question 8 | Numeric Value  999=missing data |
| Q9 | NEP Scale Question 9 | Numeric Value  999=missing data |
| Q10 | NEP Scale Question 10 | Numeric Value  999=missing data |
| Q11 | NEP Scale Question 11 | Numeric Value  999=missing data |
| Q12 | NEP Scale Question 12 | Numeric Value  999=missing data |
| Q13 | NEP Scale Question 13 | Numeric Value  999=missing data |
| Q14 | NEP Scale Question 14 | Numeric Value  999=missing data |
| Q15 | NEP Scale Question 15 | Numeric Value  999=missing data |
